# Dinitrogen Reduction and Functionalization by a Siloxide Supported Thulium‐Potassium Complex for the Formation of Ammonia or Hydrazine Derivatives

**DOI:** 10.1002/anie.202414051

**Published:** 2024-11-09

**Authors:** R. A. Keerthi Shivaraam, Thayalan Rajeshkumar, Rosario Scopelliti, Ivica Z̆ivković, Laurent Maron, Marinella Mazzanti

**Affiliations:** ^1^ Group of Coordination Chemistry Institut des Sciences et Ingénierie Chimiques Ecole Polytechnique Fédérale de Lausanne (EPFL) 1015 Lausanne Switzerland; ^2^ Laboratoire de Physique et Chimie des Nano-objets Institut National des Sciences Appliquées 31077 Toulouse, Cedex 4 France; ^3^ X-Ray Diffraction and Surface Analytics Platform Institute of Chemical Sciences and Engineering (ISIC) École Polytechnique Fédérale de Lausanne (EPFL) 1015 Lausanne Switzerland; ^4^ Laboratory for Quantum Magnetism Institute of Physics École Polytechnique Fédérale de Lausanne (EPFL) 1015 Lausanne Switzerland

**Keywords:** thulium, dinitrogen complexes, lanthanides, nitrogen functionalization, siloxide ligands

## Abstract

The dinitrogen (N_2_) chemistry of lanthanides remains less developed compared to the d‐block metals and lanthanide‐promoted N_2_ functionalization chemistry in well‐defined lanthanide complexes remains elusive. Here we report the synthesis and characterization (SQUID, EPR, DFT, X‐Ray) of the siloxide supported heterobimetallic (Tm/K) complexes [{KTm(OSi(O^*t*^Bu)_3_)_3_}_2_(*μ*‐*η* ^2^ : *η* ^2^‐N_2_)] (**1**) and [K_3_{Tm(OSi(O^*t*^Bu)_3_)_3_}_2_(*μ*‐*η* ^2^ : *η* ^2^‐N_2_)] (**2**). Complex **2** provides a rare example of a metal complex of the triply reduced N_2_^3−^ radical. The structure of **2** differs from the few previously reported N_2_^3−^ complexes as it presents two Tm and three K cations binding the N_2_^3−^ radical, facilitating N_2_ functionalization. Notably, the K_3_Tm_2_‐bound N_2_^3−^ moiety reacts with excess H^+^ to form NH_4_Cl in 18 % yield, and with MeOTf at room temperature to yield the dimethyl hydrazido complex [K_2_{Tm(OSi(O^*t*^Bu)_3_)_3_}_2_(*μ*‐(CH_3_)NN(CH_3_))] (**3**). Protonolysis of **3** yields MeHN−NMeH ⋅ 2HCl in 18 % yield.

## Graphical abstract

Siloxide ligands promote dinitrogen (N_2_) reduction by Tm(II) ions yielding an N_2_^2−^ bridged complex that can be further reduced to yield a complex showing a rare triply reduced N_2_^3−^ radical bound by two Tm and three K cations, that facilitate N_2_ functionalization by electrophiles (H^+^, Me^+^) and N−C bond formation to yield an Me_2_N_2_^2−^ complex.

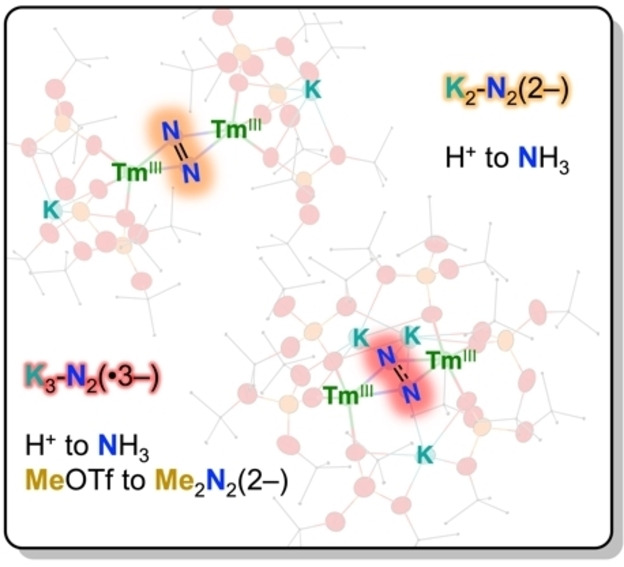

The binding, reduction, and functionalization of dinitrogen by metal centers continue to attract many studies because N_2_ is a potential abundant source of nitrogen for the synthesis of ammonia and higher value nitrogen‐containing products. However, metal‐promoted N_2_ functionalization still poses a challenge in chemistry[^1^, ^2^, ^3^, ^4^, ^5^, ^6^] and in particular examples of conversion of N_2_ to hydrazine derivatives containing N−C bonds remain rare[^2^, ^3^] with only two examples mediated by rare earth metals.[^7^, ^8^]

Several examples of dinitrogen functionalization by H^+^, H_2_, CO, CO_2_ and chlorosilanes promoted by uranium complexes were reported recently[^9^, ^10^, ^11^, ^12^] but the dinitrogen chemistry of molecular compounds of the f block,[^13^, ^14^] remains significantly less developed compared to d‐block metals, although their unique electronic properties[^15^, ^16^] can lead to the stabilization of unusual species and reactivity.

Notably, complexes containing the triply reduced N_2_^3−^ radical were first isolated for the dysprosium (**A**) ion by Evans and co‐workers, and led to the rational synthesis of N_2_^3−^ complexes of lanthanides (**B**) (Figure 1) that showed exceptional magnetic properties.[^17^, ^18^, ^19^, ^20^]

**Figure 1:**
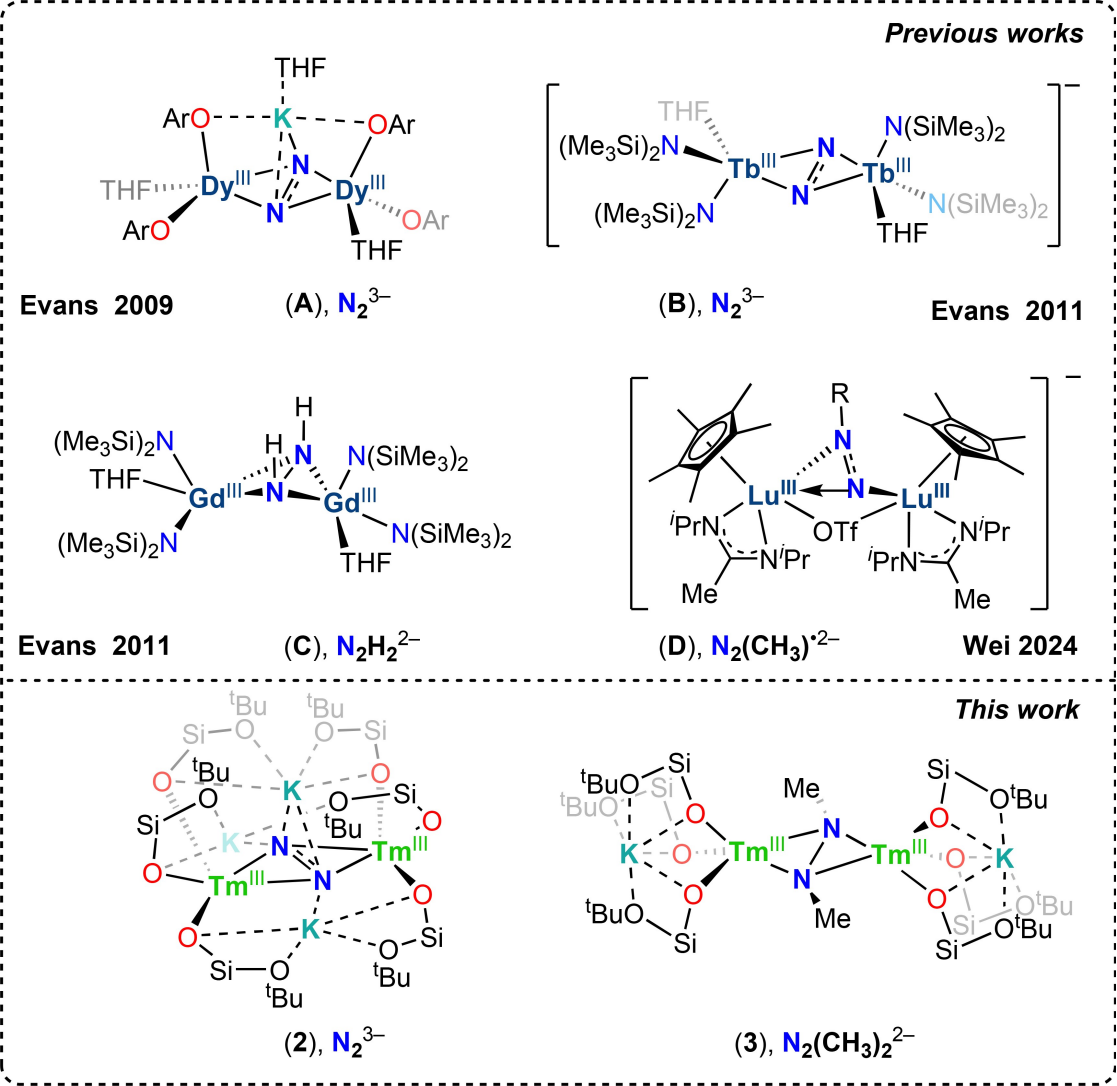
Selected lanthanide complexes of N_2_ and of functionalized N_2_^3−^ (above). N_2_^3−^ and hydrazido lanthanide complexes from this work (below) (only the −O^t^Bu groups of OSi(O^t^Bu)_3_ that are metal bound are shown).

However, N_2_^3−^ complexes remain rare and limited to lanthanides, Sc and Y supported by amides, aryloxides or cyclopentadienyl ligands. Moreover, functionalization of bound dinitrogen in isolated lanthanide complexes remains elusive with only two examples (**C** and **D**) (Figure 1) reported so far.[^8^, ^21^]

Lanthanide dinitrogen complexes containing the side‐on N_2_^2−^ bridging moiety are known for several supporting ligands[^22^, ^23^, ^24^, ^25^, ^26^, ^27^, ^28^, ^29^, ^30^] and were reported to act as a two‐electron reservoir for the reduction of a broad range molecules such as bipyridine,^[31]^ CO, CO_2_, azobenzene, anthracene, cyclooctatetraene or phenazine, but this reactivity resulted in the release of N_2_.[^32^, ^33^] Moreover, despite the potential interest of N_2_^3−^ species for accessing new functionalization pathways,^[34]^ their reactivity remains poorly studied.[^35^, ^36^] In 2011 Evans and co‐workers reported the reaction of the N_2_^3−^ complex {[(Me_3_Si)_2_N]_2_(THF)Gd}_2_(*μ*‐*η*_2_ : *η*_2_‐N_2_)}^−^ to yield the first (N_2_H_2_)^2−^ lanthanide complex derived from N_2_ ^[21]^ and observed the same reactivity for the analogous of N_2_^3−^‐yttrium(III) complex. In 2019, Xi and co‐workers^[8]^ showed that the N_2_^3−^ moiety bridging two scandium(III) centers in the [{(C_5_Me_5_){^*n*^BuC(N^*i*^Pr)_2_)}Sc}_2_(*μ*‐*η*_2_ : *η*_2_‐N_2_)] complex could be functionalized with MeOTf at room temperature to yield a dimethylhydrazine derivative. However, Wei and co‐workers^[8]^ very recently showed that the same reactivity could not be extended to the analogous complex of the lanthanide ion lutetium, but a unique monomethylated (NN−Me)⋅^2−^ product (**D**) could be instead isolated at very low temperature.

Here we report a new trianionic N_2_^3−^ complex of thulium supported by tris‐tertbutoxysiloxide ligands which was prepared by the reduction of the dianionic N_2_^2−^ analogue. We show that in this complex the bridging triply reduced dinitrogen is sufficiently activated to allow its functionalization by electrophiles at room temperature leading to the third example of direct N−C formation from N_2_ promoted by a rare‐ earth metal.

Our group[^37^, ^38^, ^39^, ^40^] previously reported that the HOSi(O^*t*^Bu)_3_ ligand can be used to synthesize complexes of Sm and Yb in the +2 oxidation state by protonolysis of the [Ln((Me_3_Si)_2_N)_2_] (Ln=Sm and Yb) precursors. The isolated [Ln_2_((O^*t*^Bu)_3_SiO)_4_] (Ln=Sm and Yb) complexes showed high reactivity towards the reduction of arenes^[38]^ and small molecules such as CO_2_ and CS_2,_[^39^, ^40^] but did not react with N_2_ (1 atm) in THF, Et_2_O or n*‐*hexane. Therefore we explored the possibility of accessing analogous siloxide complexes of the more reducing Tm ion[^24^, ^41^, ^42^, ^43^] (*E*_1/2_=−2.3 V for Tm versus −1.5 V for Sm).^[44]^

The reaction of [Tm((Me_3_Si)_2_N)_2_] generated in situ, with HOSi(O^*t*^Bu)_3_ only resulted in Tm(III) species probably due to the high reactivity of the Tm(II) precursor in common organic solvents.^[24]^ In contrast, upon reacting TmI_2_ and 2.0 equiv. of KOSi(O^*t*^Bu)_3_ in Et_2_O at room temperature under nitrogen for 12 days, the ^1^H NMR spectrum of the resulting yellow‐orange supernatant (in *d_12_*‐cyclohexane, Figure S1) showed the presence of a major species at 16.91 ppm identified as the complex [{KTm(OSi(O^*t*^Bu)_3_)_3_}_2_ (*μ*‐*η* ^2^ : *η* ^2^‐N_2_)] (**1**) that could be isolated as a crystalline solid in 18 % yield from a concentrated hexane solution at −40 °C (Scheme 1 and Figure 2a)). Attempts to increase the reaction yield by reacting TmI_2_ with 3.0 equiv. of KOSi(O^*t*^Bu)_3_ under N_2_ in Et_2_O led to the formation of **1** but also of additional multiple species that could not be identified (refer to Supporting Information, Figure S6). The cleaner formation of complex **1** when using a 1 : 2 Tm : ligand ratio, suggests that the formation of **1** involves a non‐straightforward mechanism rather than just preferential formation of **1**.

**Scheme 1:**
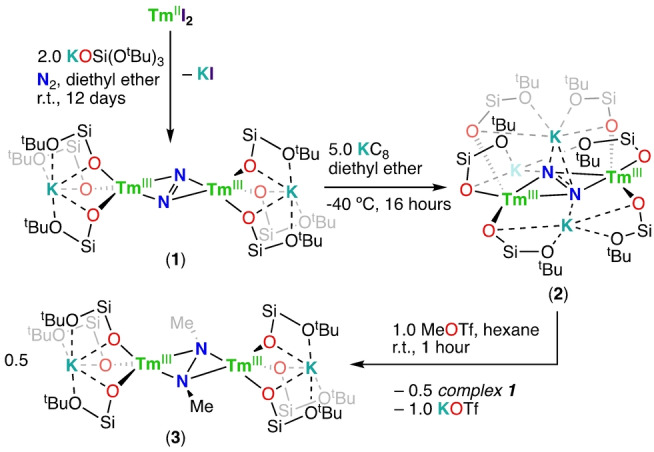
Synthesis of complexes **1**, **2** and **3** (in all complexes, only the −O^t^Bu groups of −OSi(O^t^Bu)_3_ that are metal bound are shown).

**Figure 2:**
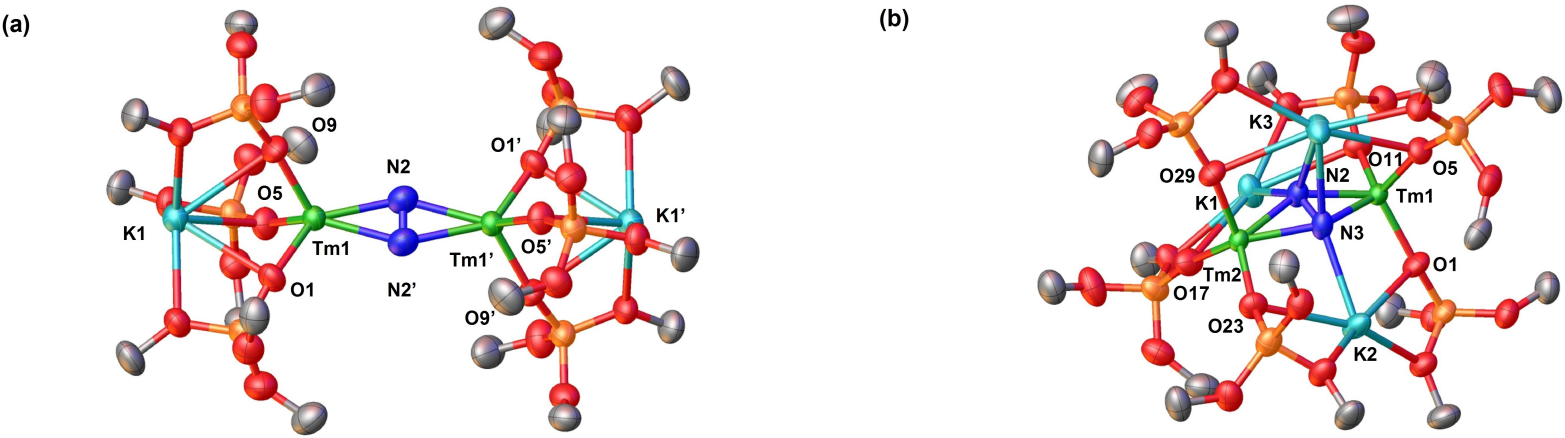
Molecular structures of complexes (a) **1** and (b) **2** with thermal ellipsoids drawn at the 50 % probability level. Methyl groups, hydrogen atoms and disordered positions have been omitted for clarity.

Complex **1** is stable for at least a month in the solid state at −40 °C, whilst in the solution state at room temperature significant decomposition could be observed immediately (in *d_8_*‐THF) or after 7 days (in *d_12_*‐cyclohexane) (Figure S15 and Figure S14 respectively). The X‐Ray determined solid‐state structure^[45]^ of **1** (Figure 2a) showed the presence of a dinuclear complex where each Tm ion is bound by three anionic siloxide ligands and a side‐on bridging N_2_ moiety. Two potassium cations are bound within the inner coordination sphere of the complex by the six siloxide ligands. The Tm−O_siloxide_ bond lengths (2.102(2)–2.113(2) Å) are comparable with those found for the [Tm^III^(OSi(O^*t*^Bu)_3_)_2_(*μ*‐OSi(O^*t*^Bu)_3_)]_2_ (**4**) (Tm1−O_siloxide_=2.076(7) Å, 2.088(6) Å) complex independently prepared by reacting [Tm{N(SiMe_3_)_2_}_3_] with 3.0 equiv. HOSi(O^*t*^Bu)_3_ in hexane (refer Supporting Information). The N−N bond length (1.19(4) Å) in **1** compares well with the values (in the range 1.043(14) Å to 1.193(9) Å) found in the few dianionic side‐on diazenido (N_2_^2−^) complexes {[((Me_3_Si)_2_N)_3_Ln]_2_[*μ*‐*η* ^2^ : *η* ^2^‐N_2_]}^2−^ featuring three ligands per metal center reported recently[^46^, ^47^] for Nd, Gd and Dy. Complex **1** features a rare example of a dianionic side‐on diazenido bridged lanthanide complex supported by three anionic ancillary ligands at each metal center and the first one identified for the late lanthanide ion thulium. Considering that in contrast to complex **1**, all previously reported {[((Me_3_Si)_2_N)_3_Ln]_2_[*μ*‐*η* ^2^ : *η* ^2^‐N_2_]}^2−^ complexes were only isolated in the presence of 18‐crown‐6 crown ether or 2.2.2‐cryptand, resulting in the presence of outer‐sphere cations,[^46^, ^47^] we investigated the effect of removing the inner sphere cation from complex **1** on the structure.

The addition of a solution of 2.2.2‐cryptand (2.0 equiv.) in *d_8_*‐THF to a solution of **1** in *d_8_*‐THF resulted in the appearance of new resonances between 2.50 ppm and 4.50 ppm in the ^1^H NMR spectrum (Figure S5), signifying that the potassium ions in **1** remain bound in the inner coordination sphere upon dissolution in THF and can be removed upon the addition of 2.2.2‐cryptand. Yellow crystals of [K(crypt)]_2_[{Tm(OSi(O^*t*^Bu)_3_)_3_}_2_(*μ*‐*η* ^2^ : *η* ^2^‐N_2_)] **1‐crypt** (crypt=2.2.2‐cryptand) could be grown from a concentrated solution of **1‐crypt** in diethyl ether at −40 °C (refer to Supporting Information, Figure S45). The N−N bond length of 1.271(6) Å in **1‐crypt** is longer than that found for complex **1**, suggesting a greater degree of dinitrogen reduction in the absence of inner‐sphere alkali metal ions which can be related to the increased electron donation from the siloxide ligands to the Tm center.

Complex **1** can be reduced with 5.0 equiv. of KC_8_ in Et_2_O at −40 °C to yield the dinitrogen complex [K_3_{Tm(OSi(O^*t*^Bu)_3_)_3_}_2_(*μ*‐*η* ^2^ : *η* ^2^‐N_2_)] (**2**) that could be isolated in 22 % yield from toluene at −40 °C (Scheme 1 and Figure 2b)). The ^1^H NMR spectrum (Figure S30) of isolated **2** in *d_12_*‐cyclohexane features only one signal at 5.20 ppm. The addition of a solution of 2.2.2‐cryptand (3.0 equiv.) in *d_8_*‐THF to a solution of **2** in *d_8_*‐THF results in significant changes in the ^1^H NMR spectrum (Figure S34) suggesting that the potassium cations in **2** remain bound in the inner coordination sphere in THF solution and can be removed by addition of 2.2.2‐cryptand. The X‐Ray solid‐state structure of **2** (Figure 2b) features a dinuclear thulium complex with three anionic ligands per thulium center and a triply reduced dinitrogen moiety bridging the two metals in side‐on manner. In contrast to **1**, the three siloxide‐bound potassium ions are within the central core in **2**, thereby stabilizing the triply reduced dinitrogen moiety through electrostatic interactions. Complex **2** is the first reported Tm(III) complex of triply reduced N_2_ and the first example of a trianionic N_2_^3−^ bridged complex wherein each metal center is supported by three anionic ligands. The N−N bond length value of 1.22(6) Å in **2** is longer compared to that of **1** (1.19(4) Å) suggesting a greater degree of dinitrogen reduction. The measured N−N bond length values in **2** are shorter compared to N_2_^3−^ bridged lanthanide complexes reported in literature (values 1.362(9) Å–1.402(7) Å) for dianionic complexes presenting only two ancillary ligands per metal center.[^18^, ^19^, ^20^, ^48^] The measured Raman spectra of **1** and **2** are very similar except for the presence in **2** of the vibrational frequency assigned to the N−N stretching mode found at 999.6 cm^−1^ (Figure S68), a value close to those found in previously reported Ln_2_−N_2_^3−^ complexes (989 cm^−1^).^[48]^

Magnetic susceptibility data (refer Supporting Information section E) were collected for complexes **1** and **2** in the range of 2 K to 300 K, under an applied DC field of 1 T (Figure S50–S61). Complex **1** has a χT value of 8.4 emu K mol^−1^ per ion at 300 K (16.8 emu K mol^−1^ per complex) that decreases rapidly with decreasing temperature, reaching 1.6 emu K mol^−1^ per ion at 2 K (3.2 emu K mol^−1^ per complex), in good agreement with the presence of two non‐interacting free 4f^12^ ions.^[49]^ Complex **2** shows a significantly different behavior with temperature consistent with a delocalized bonding description. The χT value for the complex **2** of 10.7 emu K mol^−1^ at 300 K is significantly lower than the value of 15.5 emu K mol^−1^ per complex expected for two magnetically isolated Tm(III) ions and a single radical S=1/2 N_2_^3−^ unit in agreement with the delocalization of the electron over the N_2_ and the two metal centers observed by computational studies, but could also suggest the presence of antiferromagnetic coupling. A similar behavior was reported for other N_2_^3−^ bridged lanthanide complexes.^[20]^

DFT calculations (B3PW91) were carried out on complexes **1** and **2** including dispersion corrections. In both complexes the Tm−O distances are well reproduced (2.08–2.11 Å in **1** and 2.12–2.14 Å in **2**). The Tm−N distances are in the 2.27–2.28 Å range for **1** and 2.18–2.20 Å for **2**. The N−N bond lengths are also correctly described (1.19 Å in **1** and 1.22 Å in **2**). The bonding was thus analyzed using Natural Bonding Orbital (NBO) analysis. At the NBO, the N_2_ moiety in **1** displays a double bond character (Table S6) and the associated N−N Wiberg Bond Index (WBI) is 1.95 in line with a double bond and therefore a N_2_^2−^ ligand. The latter is corroborated by the nature the HOMO (doubly occupied), that implies the in‐plane N−N π* (Figure S69) and well as the depiction of the unpaired spin density plot (Figure 3), that is only located at the Tm centers. At the second order donor‐acceptor NBO we observe donation from the siloxide ligand to the Tm center but also to the K ion resulting in a low degree of N_2_ reduction compared to the analogous K‐cryptand complex **1‐crypt** where the increased electron density at the Tm center results in a longer N−N bond.

**Figure 3:**
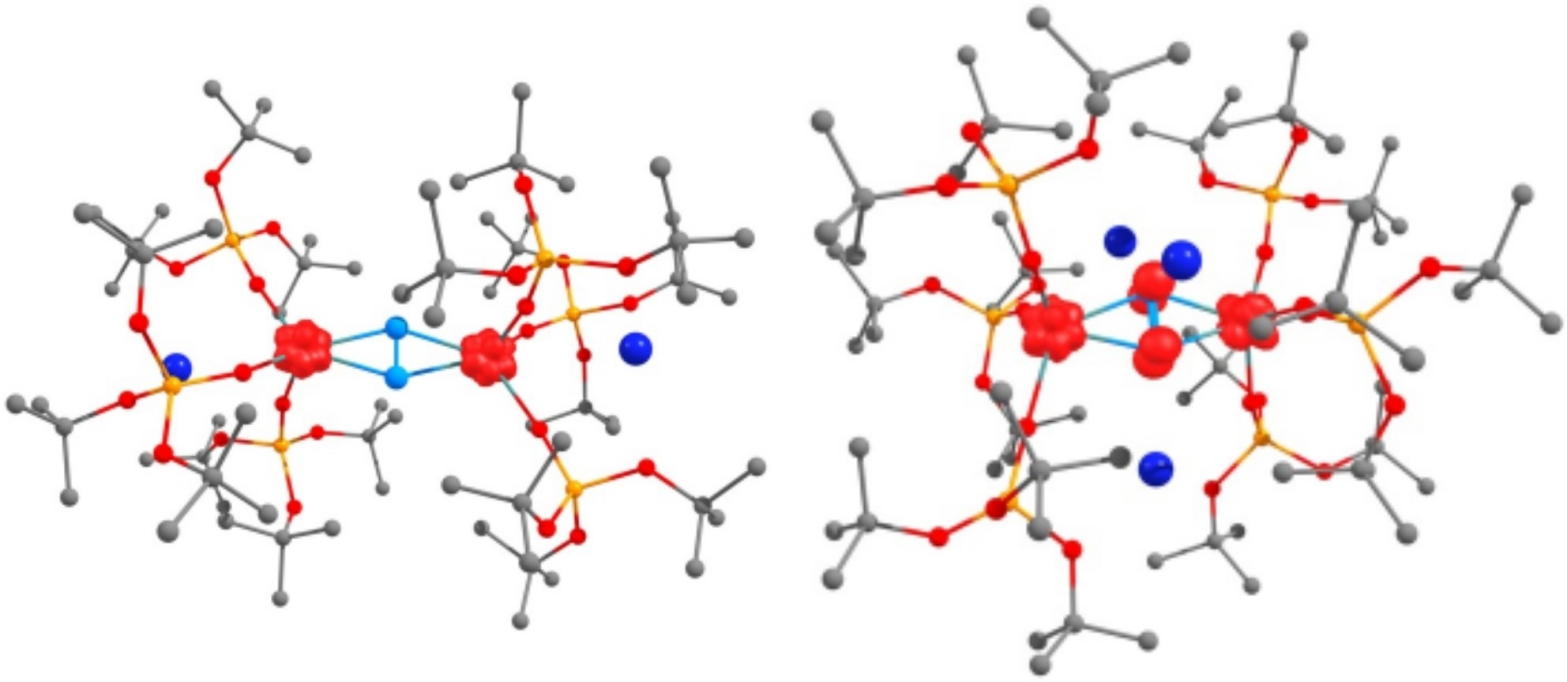
Unpaired spin density plot of complex **1** (left) and complex **2** (right). The isocontour value is set to default (0.03).

At the NBO level, four Tm−N bonds are found in **2** which are strongly polarized towards N. These four σ bonds involve a *d* orbital at Tm (95 %) and sp orbitals on N. This is in line with an almost full disruption of the π system of N_2_. The unpaired spin density plot (Figure 3) indicates some unpaired density at the N_2_ moiety so that one can safely conclude on the presence of a N_2_^3−^ ligand. EPR and magnetic data are consistent with the presence of a N_2_^3−^ and Tm(III) ions (refer to Supporting Information). The MO (Figure S70) also indicates that the two π* are involved in the SOMO (singly occupied) and HOMO−1 (doubly occupied) in line with almost full disruption of the N_2_ π system. At the second order NBO, it is interesting to note that the Tm−N σ bonds are delocalized toward the potassium cation and at the same time some delocalization from the oxygen of the siloxide ligand into the Tm−N σ* is observed. This leads to a reduction of the Tm−N interaction in favor of the N−N one, explaining the relatively short N−N distance found for the N_2_^3−^ ligand.

With complexes **1** and **2** in hand their reactivity towards electrophiles was investigated. Addition of excess HCl (2 M in diethyl ether) to isolated complexes **1** and **2** led to the formation of NH_4_Cl in 17 % and 18 % respectively (confirmed by ^1^ H NMR spectroscopy of the resulting reaction mixture in *d*_6‐_DMSO with dimethylsulfone as the internal standard) (Figure S16 and Figure S36 respectively). A similar yield in ^15^NH_4_Cl (19 %) was obtained for the ^15^N_2_ labeled complex **1** prepared in situ. Ammonia formation upon protonation was not reported so far for dinitrogen complexes of lanthanides in the absence of external reducing agents and indicates a considerable degree of activation of N_2_ in these complexes. The reaction of **2** with 1.0 equiv. of MeOTf in hexane led to an immediate color change from orange to lime yellow. Analysis of the ^1^H NMR spectrum (in *d_12_*‐cyclohexane, Figure S38) of the crude reaction mixture revealed the complete consumption of **2** and the formation of complex **1** and new resonances at 13.26 ppm and 4.47 ppm which have been assigned to the dimethylhydrazine complex [K_2_{Tm(OSi(O^*t*^Bu)_3_)_3_}_2_(*μ*‐(CH_3_)NN(CH_3_))] (**3**) (Scheme 1 and Figure 4). Colorless crystals of **3** suitable for X‐ray diffraction could be obtained from a concentrated reaction mixture in toluene at −40 °C. Bulk isolation of complex **3** could not be achieved due to the co‐crystallization of **1** along with complex **3** irrespective of the solvent utilized. Protonolysis of the reaction mixture (obtained after reacting **2** with 1.0 equiv. MeOTf) with excess of HCl resulted in the formation of MeHN−NMeH ⋅ 2HCl as confirmed by ^1^H NMR spectroscopy (dimethylsulfone as external standard, refer Supporting Information), with a yield of 18 % (Figures S43). The formation of complexes **3** and **1** is interpreted in terms of a formal disproportionation of a putative N_2_(CH_3_)⋅^2−^ radical intermediate to yield N_2_^2−^ and N_2_(CH_3_)_2_^2−^. This hypothesis is corroborated by the very recent report of a lutetium complex of the monomethylated hydrazine radical (**D**) (Figure 1) isolated from the low temperature reaction of N_2_^3−^‐Lu complex with MeOTf.^[8]^ However, only the dimethylated hydrazine complex could be isolated from the reaction of **2** with 1.0 equiv. MeOTf even at low temperature. The N−N bond length of 1.38(5) Å in **3** is longer than that found in complexes **1** and **2**, and compares well with that found in previously reported dinitrogen derived hydrazido complexes.[^8^, ^50^, ^51^, ^52^, ^53^] The formation of complex **3** (or of dimethylhydrazine after acid quenching) was not observed when MeOTf (1.0 equiv.) was reacted with **2** in hexane after addition of 2.2.2‐cryptand, indicating that N_2_ functionalization is suppressed by the removal of the potassium ions (refer to Supporting Information).

**Figure 4:**
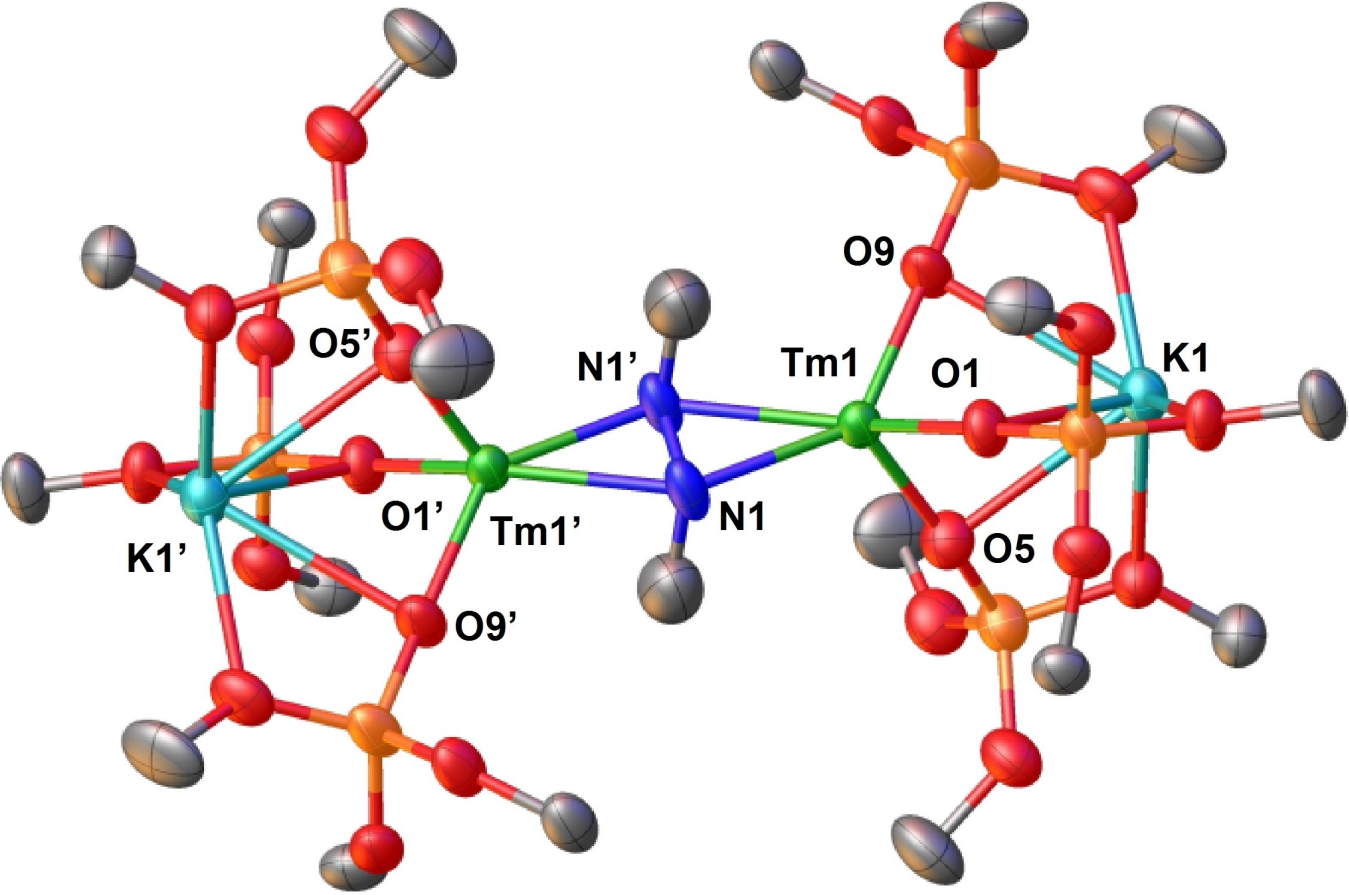
Molecular structure of complex **3** with thermal ellipsoids drawn at the 50 % probability level. Methyl groups and hydrogen atoms have been omitted for clarity.

In summary we have isolated and characterized N_2_^2−^ and N_2_^3−^ bridged dinuclear complexes of Tm(III) supported by siloxide ligands which present two and three alkali ions respectively, bound in the inner coordination sphere. Structural and DFT computational studies performed on complexes **1** and **2** are in agreement with the presence of doubly and triply reduced dinitrogen respectively and Tm(III) ions, and suggest that activation of dinitrogen is tuned by the presence of alkali ions. The six siloxide ligands stabilize the binding of three alkali ions to the N_2_^3−^ core and promote the functionalization of the bound dinitrogen with electrophiles such as MeOTf and H^+^ yielding a dimethyl hydrazine complex and ammonia respectively. This reactivity presents the second example of N−C dinitrogen functionalization promoted by a lanthanide complex and a rare example of metal mediated conversion of N_2_ to a hydrazine derivative.

## Conflict of Interests

The authors declare no conflict of interest.

## Supporting information

As a service to our authors and readers, this journal provides supporting information supplied by the authors. Such materials are peer reviewed and may be re‐organized for online delivery, but are not copy‐edited or typeset. Technical support issues arising from supporting information (other than missing files) should be addressed to the authors.

Supporting Information

## Data Availability

Synthetic details, analytical data including depictions of all spectra and coordinate data of all computationally optimised species, are documented in the Supplementary Information. Crystallographic data is made available via the CCDC. The data that support the findings of this study are openly available in the Zenodo repository at https://doi.org/10.5281/zenodo.14046745.
