# Supplementary material for: Dinitrogen Reduction and Functionalization by a Siloxide Supported Thulium‐Potassium Complex for the Formation of Ammonia or Hydrazine Derivatives
Source: Angew Chem Int Ed Engl. 2024 Nov 9;64(1):e202414051. doi: 10.1002/anie.202414051 (PMC11701356; doi:10.1002/anie.202414051)
Supplement: Supplementary file 1 — Supporting Information [file ANIE-64-e202414051-s001.pdf]

Angewandte Chemie
Eine Zeitschrift der Gesellschaft Deutscher Chemiker

Supporting Information

Dinitrogen Reduction and Functionalization by a Siloxide Supported Thulium-Potassium Complex for the Formation of Ammonia or Hydrazine Derivatives

*R. A. K. Shivaraam, T. Rajeshkumar, R. Scopelliti, I. Z|uivković, L. Maron, M. Mazzanti**

Supporting Information
©Wiley-VCH 2021
69451 Weinheim, Germany

Dinitrogen Reduction and Functionalization by a Siloxide Supported Thulium-Potassium Complex

R. A. Keerthi Shivaraam,[a] Thayalan Rajeshkumar,[b] Rosario Scopelliti,[c] Ivica Zivkovic,[d] Laurent Maron[b] and Marinella Mazzanti. *[a]

Abstract: The dinitrogen (N_2) chemistry of lanthanides remains less developed compared to the d-block metals and lanthanide-promoted N_2 functionalization chemistry in well-defined lanthanide complexes remains elusive. Here we report the synthesis and characterization (SQUID, EPR, DFT, X-Ray) of the siloxide supported heterobimetallic (Tm/K) complexes [{KTm(OSi(O^{t}Bu)$_3$)$_3$}$_2$(μ-η^2:η^2-N_2)] (**1**) and [K_3{Tm(OSi(O^{t}Bu)$_3$)$_3$}$_2$(μ-η^2:η^2-N_2)] (**2**). Complex **2** provides a rare example of a metal complex of the triply reduced N_2^{3-} radical. The structure of **2** differs from the few previously reported N_2^{3-} complexes as it presents two Tm and three K cations binding the N_2^{3-} radical, facilitating N_2 functionalization. Notably, the K_3Tm_2-bound N_2^{3-} moiety reacts with excess H^+ to form NH_4Cl in 18% yield, and with MeOTf at room temperature to yield the dimethyl hydrazido complex [K_2{Tm(OSi(O^{t}Bu)$_3$)$_3$}$_2$(μ-(CH_3)NN(CH_3))] (**3**). Protonolysis of **3** yields MeHN-NMeH·2HCl in 18 % yield.

DOI: 10.1002/anie.2021XXXXX

Table of Contents

A. Experimental Procedures
B. NMR Spectroscopic Data
C. X-Ray Crystal Structure Determination Details
D. EPR Spectroscopic Data
E. SQUID Magnetometry Data
F. UV-Vis/NIR Spectra
G. IR Spectra
H. Raman Spectra
I. Computational Details
J. References

A. Experimental Procedures

General Considerations. Unless otherwise noted, all manipulations were carried out at ambient temperature under a dinitrogen atmosphere using Schlenk techniques and an MBraun glovebox equipped with a purifier unit. The water and oxygen levels were always kept at less than 0.1 ppm. Glassware was dried overnight at 140 °C before use. Syntheses were performed using glass coated stirrer bars.

NMR experiments were carried out using NMR tubes adapted with J-Young valves. NMR spectra were recorded on a Bruker 400 MHz or 600 MHz spectrometers. NMR chemical shifts are reported in ppm with solvent as internal reference Elemental analyses were performed under nitrogen using a Thermo Scientific Flash 2000 Organic Elemental Analyzer at the Institute of Chemistry and Chemical Engineering at EPFL.

EPR analyses were performed on a Bruker Elexsys E500 spectrometer working at 9.4 GHz frequency with an Oxford ESR900 cryostat for 4-300 K operation.

Magnetic measurements were performed using a Quantum Design MPMS3 superconducting quantum interference device (SQUID) magnetometer in a temperature range 2-300 K. The microcrystalline sample was restrained with eicosane and enclosed in a quartz capsule packed under dinitrogen and placed inside a plastic straw. The measurements were performed with applied magnetic field of 1 T in the zero-field cooled (ZFC) regime. Diamagnetic corrections were applied using Pascal's constants.[1] The magnetic moment per complex was calculated using the following formula:

$$\mu_{eff} = \sqrt{8\chi_{corr}T}$$

where $\chi_{corr} = \chi_{measured} - \chi_{dia}$, χ_{dia} was calculated using Pascal's constants.[1]

UV/Vis/NIR spectra were recorded as solutions in diethyl ether using 1 mm cuvettes equipped with a J-Young valve and a Perkin Elmer 950 spectrometer.

FT-IR spectra were recorded with a Perkin Elmer 1600 Series FTIR spectrophotometer flushed with dinitrogen.

Raman spectra were recorded with an XploRA PLUS Raman microscope using using a 532 nm laser. Crystalline samples were loaded into quartz capillaries (OD = 1.5 mm, L = 80 mm, wall thickness 0.01 mm) and sealed under inert atmosphere of dinitrogen.

Starting materials. Unless otherwise noted, reagents were purchased from commercial suppliers and used without further purification. Tris(*tert*-butoxy)silanol was purified by sublimation. TmI_2 was purchased from either Alfa Chemistry or Chemicals 101 Corp. 18-crown-6 ether and 2.2.2-cryptand were dried under vacuum at room temperature for one week prior to use. N,N'-dimethylhydrazine dihydrochloride was purchased from Aldrich as a reference material. Anhydrous solvents were purchased from Aldrich and further distilled from K/benzophenone (THF, toluene, diethyl ether), sodium sand/benzophenone (*n*-hexane). Deuterated solvents for NMR spectroscopy (d_8-THF) were purchased from Cortecnet, freeze-degassed and distilled over K/benzophenone. d_6-DMSO and d_{12}-cyclohexane were freeze-degassed and dried over 3 Å molecular sieves for several days. $^{15}N_2$ (98% ^{15}N) was purchased from Cortecnet and transferred to a flask equipped with a J. Young valve and activated 3 Å molecular sieves prior to use. $[Tm\{N(SiMe_3)_2\}_3]$, $KOSi(O^tBu)_3$ and KC_8 were synthesized according to literature procedures.[2–4] $[Tm(OSi(O^tBu)_3)_2(\mu\text{-}OSi(O^tBu)_3)]_2$ (**4**) was synthesized using a modified procedure based on the syntheses previously reported for analogous $[Ln(OSi(O^tBu)_3)_2(\mu\text{-}OSi(O^tBu)_3)]_2$ complexes of other lanthanides(Ln = Ce, La, Nd, Yb, Lu and Sm).[5–7]

Synthesis of [{KTm(OSi(O^{t}Bu)$_3$)$_3$}$_2$(μ-η^2:η^2-N$_2$)].(hexane)$_2$ (1)

A colorless solution of KOSi(O^{t}Bu)$_3$ (731.7 mg, 2.42 mmol, 2.0 equiv.) in Et_2O (10 mL) at r.t. was added to a stirring brown suspension of TmI_2 (511.1 mg, 1.21 mmol, 1.0 equiv.) in Et_2O (10 mL) at r.t. No observable colour change was perceived during the addition. The reaction mixture was stirred vigorously at r.t. for 12 days resulting in a yellow-orange supernatant and a white/greyish white precipitate (*NOTE*: The headspace of the reaction vessel was evacuated and backfilled with dinitrogen from the glovebox atmosphere three times every day during the 12-day reaction period). A red-brown precipitate was observed after the first day of the reaction, which was slowly consumed during the 12-day reaction period to yield the final yellow-orange suspension. The volatiles were then evaporated under reduced pressure and the resulting residue was suspended in hexane (10 mL) and filtered over a 0.2 µm porosity filter to remove KI. The yellow-orange filtrate obtained was concentrated to 2 mL and placed at -40 °C for two days. The yellow microcrystalline solid formed was filtered over a prechilled (-40 °C) porosity 4 glass filter frit, affording **1** as a yellow microcrystalline solid (101.7 mg, 0.05 mmol) (*NOTE*: Crystals of **1** dissolve easily if the temperature is not kept low during the work up). A second crop (37.3 mg, 0.02 mmol) could be obtained by further concentration of the mother liquor to 1 mL. The combined yield of both the crops is 18% (with respect to the ligand i.e. yield calculated with respect to the stoichiometry used under the assumption that all the siloxide ligand was used to form the isolated complex). Yellow crystals of **1** suitable for X-Ray diffraction were grown from a concentrated solution of **1** in hexane at -40 °C. ^{1}H NMR (400 MHz, d_{12}-cyclohexane, 298 K) δ= 16.91 (s, -(O^{t}Bu)) (**Figure S3**). Elemental analysis calcd (%) for [{KTm(OSi(O^{t}Bu)$_3$)$_3$}$_2$(μ-η^2:η^2-N$_2$)] (2024.65 g mol^{-1}): $C_{72}H_{162}N_2O_{24}Si_6K_2Tm_2$: C 42.71, H 8.07, N 1.38; found: C 42.83, H 8.11, N 0.48 (lower values for nitrogen could be attributed to partial dinitrogen loss during combustion despite multiple attempts). Complex **1** is stable for at least a month at -40 °C in the solid state stored under N_2. Complex **1** is stable for at least one day in the solution state (cyclohexane) at r.t., and significant decomposition could be observed after one week (**Figure S14**). *NOTE*: At first we pursued the reaction of TmI_2 and 2.0 equiv. of KOSi(O^{t}Bu)$_3$ in THF under a dinitrogen atmosphere. Irrespective of the temperature at which the reaction was performed (-80 °C, -40 °C) the ^{1}H NMR spectrum of the resulting reaction mixture showed multiple species which could not be isolated (*vide infra*, **Figure S10** and **Figure S11**). Performing the salt metathesis reaction under argon in diethyl ether/hexane also led to the formation of a red brown precipitate after one day, which immediately forms a green oil upon extraction into a polar solvent such as THF. The long reaction time (i.e. twelve days) required to obtain **1** could be explained due to the heterogeneous nature of the reaction, as a more diluted smaller scale reaction is complete within six days as confirmed by analysis of the reaction mixture by ^{1}H NMR spectroscopy in d_{12}-cyclohexane (*vide infra*, **Figure S2**b)). Examining the reaction mixture by ^{1}H NMR spectroscopy after just one day (*vide infra*, **Figure S2**a))revealed a mixture of **1** at 16.91 ppm and an intermediate species at 5.19 ppm which is formed initially, however is consumed by the end of the reaction period. Performing the salt metathesis reaction of TmI_2 with 2.0 equiv. of KOSi(O^{t}Bu)$_3$ under argon in diethyl ether led to the formation of a red brown precipitate after one day, which when exposed to N_2 for six days converts into complex **1** and other unidentified species (*vide infra*, **Figure S7**). Although the composition/identity of the species obtained from the reaction performed under argon could not be determined due to the presence of multiple species and decomposition products, its ability to react with N_2 supports the hypothesis of the intermediate red brown precipitate being a thulium(II) species responsible in the formation of **1**.

Attempts to prepare **1** by the alternative route developed by Evans and coworkers[8] for the synthesis of the [K(crypt)]$_2$[{(NR$_2$)$_3$Dy}$_2$(μ-η^2:η^2-N$_2$)] complex, performing the reduction of the trivalent *tris*-ligand (i.e. three ligands per metal) complex [Tm^{III}(OSi(O^{t}Bu)$_3$)$_2$(μ-OSi(O^{t}Bu)$_3$)]$_2$ with KC_8 under nitrogen in the presence or absence of encapsulating agents such 18-crown-6 only led to the isolation of Tm(III) scrambling products (*vide infra*).

The low yield of the salt metathesis route is likely due to both the high solubility of the complex **1** and its partial decomposition over the long time required to complete the heterogeneous reaction that results in the solubilization under N_2 of an intermediate red-brown solid that forms soon after mixing the reagents. Unfortunately, the identity of this red brown species remains unknown and could not be characterized by ^{1}H NMR spectroscopy or X-ray crystallography due to its limited solubility in the solvents utilized (i.e. diethyl ether, toluene, cyclohexane and hexane).

Synthesis of [K$_3${Tm(OSi(O^{t}Bu)$_3$)$_3$}$_2$(μ-η^2:η^2-N$_2$)] (2)

A yellow solution of **1** (95.5 mg, 0.04 mmol, 1.0 equiv.) in Et_2O (5.0 mL) at -40 °C was added to solid KC_8 (29.4 mg, 0.20 mmol, 5.0 equiv.) at -40 °C. No observable colour change was perceived during the addition. The reaction mixture was stirred at -40 °C overnight (16 h) affording an orange supernatant along with the presence of a black precipitate (i.e. graphite) and excess bronze KC_8. The reaction mixture was filtered over a 0.2 µm porosity filter to obtain an orange filtrate. The volatiles were evaporated under reduced pressure and the resulting orange residue was suspended in cold (-40 °C) toluene (1 mL), filtered over a 0.2 µm porosity filter and placed at -40 °C for two days. The orange microcrystalline solid formed was filtered over a prechilled (-40 °C) porosity 4 glass filter frit and the solid was dried under vacuum for five mins, affording **2** as an orange microcrystalline solid (17.4 mg, 0.008 mmol). A second crop (2.2 mg, 0.001 mmol) could be obtained by further concentration of the mother liquor to 0.5 mL. The combined yield of both the crops is 22%. Orange crystals of **2. (hexane)$_{1.5}$** suitable for X-Ray diffraction were grown from a concentrated solution of **2** in hexane at -40 °C. ^{1}H NMR (400 MHz, d_{12}-cyclohexane, 298 K) δ= 5.20 (s, -(O^{t}Bu)) (**Figure S30**). ^{1}H NMR (400 MHz, d_8-THF, 233 K) δ= 12.76 (s, -(O^{t}Bu)) (**Figure S33**). Elemental analysis calcd (%) for [K$_3${Tm(OSi(O^{t}Bu)$_3$)$_3$}$_2$(μ-η^2:η^2-N$_2$)] (2063.75 g mol^{-1}): $C_{72}H_{162}N_2O_{24}Si_6K_3Tm_2$: C 41.90, H 7.91, N 1.36; found: C 41.60, H 7.91, N 0.58 (lower values for nitrogen could be attributed to partial dinitrogen loss during combustion despite multiple attempts). Complex **2** is stable for at least a month at -40 °C in the solid state stored under dinitrogen. Complex **2** is stable for at least three days in the solution-state (in d_{12}-cyclohexane) and continues to persist after 17 days in solution

(d_{12}-cyclohexane) (**Figure S31**). Contrary to **1** (**Figure S15**), complex **2** is stable upon dissolution in THF (**Figure S32**). The relatively low yield obtained for **2** is attributed to its very high solubility (both hexane and toluene). (*NOTE*: The synthesis of complex **2** requires 5.0 equiv. of KC_8 in the aforementioned timeframe i.e. 16 h, as when 1.2 equiv. of KC_8 is used instead the starting material complex **1** is still observed when all other parameters are kept the same (refer **Figure S29**))
The addition of a solution of 2.2.2-cryptand (3.0 equiv.) in d_8-THF to a solution of **2** in d_8-THF results in significant changes in the ^{1}H NMR spectrum (from one signal at 12.76 ppm corresponding to the siloxide ligands before addition to three signals at 5.22 ppm, 4.20 ppm and 3.94 ppm after addition) suggesting that the potassium cations remain bound in the inner coordination sphere in THF solution and can be removed by addition of 2.2.2-cryptand even in the case of complex **2** (**Figure S34**).

Synthesis of [Tm(OSi(O^{t}Bu)$_3$)$_2$(*μ*-OSi(O^{t}Bu)$_3$)]$_2$ (4)

A colorless solution of HOSi(O^{t}Bu)$_3$ (181.2 mg, 0.69 mmol, 3.0 equiv.) in hexane (1 mL) at r.t. was added to a stirring colorless solution of [Tm{N(SiMe$_3$)$_2$}$_3$] (148.5 mg, 0.23 mmol, 1.0 equiv.) in hexane (4 mL) at r.t. No observable colour change was perceived during the addition. The reaction mixture was stirred at r.t. overnight (14 h) resulting in a white suspension. The white solid formed was filtered over porosity 4 glass filter frit and dried under vacuum, affording **4** as a white powder (150.9 mg, 0.08 mmol, 69% yield). Colorless crystals of **4** suitable for X-Ray diffraction were grown from a concentrated mother liquor in hexane at -40 °C after collecting the first crop of **4**. ^{1}H NMR (400 MHz, d_8-THF, 193 K) δ 10.76 (br s, -(O^{t}Bu)), -66.12 (s, -(O^{t}Bu)) (**Figure S18**). ^{1}H NMR (400 MHz, d_8-THF, 233 K) δ 4.60 (br s, -(O^{t}Bu)), 0.91 (br s, -(O^{t}Bu)), -23.35 (br s, -(O^{t}Bu)), -52.33 (br s, -(O^{t}Bu))(**Figure S19**). Elemental analysis calcd (%) for [Tm(OSi(O^{t}Bu)$_3$)$_2$(*μ*-OSi(O^{t}Bu)$_3$)]$_2$ (1918.44 g mol^{-1}): $C_{72}H_{162}O_{24}Si_6Tm_2$: C 45.08, H 8.51; found: C 44.96, H 8.60.

Reaction of complex 2 with methyl triflate to yield [K$_2${Tm(OSi(O^{t}Bu)$_3$)$_3$}$_2$(*μ*-(CH$_3$)NN(CH$_3$))] (3) and complex 1

Complex **2** was prepared *in situ* by reduction of **1** (102.0 mg, 0.05 mmol, 1.0 equiv.) in Et_2O (6 mL) at -40 °C with KC_8 (31.4 mg, 0.23 mmol, 5.0 equiv.) for 16 h. The reaction mixture was filtered over a 0.2 µm porosity filter to obtain an orange filtrate. The volatiles were evaporated under reduced pressure and the resulting orange residue obtained was redissolved in hexane (4.5 mL) and colorless methyl triflate (5.1 µL, 0.05 mmol, 1.0 equiv.) was added at r.t. whilst stirring. No observable colour change was perceived during the addition. The reaction mixture was stirred at r.t. for 1 h following which a yellow/lime supernatant was observed along with the formation of a white precipitate. The reaction mixture was then filtered over a 0.2 µm porosity filter and the yellow/lime filtrate obtained was concentrated to 0.4 mL and placed at -40 °C for two days. The yellow microcrystalline solid formed (15.0 mg) was filtered over a prechilled (-40 °C) porosity 4 glass filter frit, and the solid obtained was dried under vacuum for 5 mins. ^{1}H NMR analysis of the yellow microcrystalline solid isolated revealed the presence of complex **1** and resonances at 13.45 ppm and 4.53 ppm assigned to complex **3** (**Figure S39**). Colorless crystals of **3**.(toluene)$_{0.8}$ suitable for X-Ray diffraction were grown from a concentrated reaction mixture in toluene at -40 °C. (*NOTE*: The similar solubility of complexes **1** and **3** in hexane or toluene prevented their separation as all attempts to isolate **3** also resulted in the co-crystallization of **1**)
Hydrolysis with excess HCl (2 M in Et_2O) allowed to confirm the presence of the dimethylated hydrazine product (*vide infra*).

Reaction of complex 2 with methyl triflate at -90 °C

Complex **2** was prepared *in situ* by reduction of **1** (27.0 mg, 0.01 mmol, 1.0 equiv.) in Et_2O (1.5 mL) at -40 °C with KC_8 (8.3 mg, 0.06 mmol, 5.0 equiv.) for 16 h. The reaction mixture was filtered over a 0.2 µm porosity filter to obtain an orange filtrate. The volatiles were evaporated under reduced pressure and the resulting orange residue obtained was redissolved in hexane (0.5 mL) and cooled to -90 °C, following which a colorless solution of methyl triflate (1.3 µL, 0.01 mmol, 1.0 equiv.) in hexane (0.5 mL) at -90 °C was added. No observable colour change was perceived during the addition. The reaction mixture was stirred at -90 °C for 5 h resulting in a yellow supernatant and a white precipitate. The reaction mixture was filtered over a prechilled (-90 °C) porosity 4 glass filter frit to obtain a yellow solution. Colorless crystals of the dimethylhydrazine complex **3** were grown by placing the yellow solution obtained at -40 °C for two days, signifying the occurrence of dimethylation even at low temperatures.

Addition of 2.2.2-cryptand (3.0 equiv.) to complex 2 followed by addition of methyl triflate

Complex **2** was prepared *in situ* by reduction of **1** (12.5 mg, 0.006 mmol, 1.0 equiv.) in Et_2O (0.5 mL) at -40 °C with KC_8 (3.9 mg, 0.03 mmol, 5.0 equiv.) for 16 h. The reaction mixture was filtered over a 0.2 µm porosity filter to obtain an orange filtrate. To the obtained orange filtrate at -40 °C, a colorless solution of 2.2.2-cryptand (6.4 mg, 0.017 mmol, 3.0 equiv.) in Et_2O (0.5 mL) at -40 °C was added resulting in a red orange solution. The volatiles were evaporated under reduced pressure and the resulting orange residue was suspended in hexane (0.8 mL), following which methyl triflate (0.6 µL, 0.006 mmol, 1.0 equiv.) was added at r.t.. A white precipitate was observed a few mins after the addition whilst the orange residue was still observed. The reaction mixture was stirred at r.t. for one day resulting in a very pale green supernatant and a white precipitate. The volatiles were evaporated from the reaction mixture under reduced pressure, and cold (-80 °C) colorless solution of 1 M HCl in Et_2O (5.7 mL) was added at -80 °C. A colorless supernatant was observed along with the formation of an off-white precipitate during the addition. The reaction mixture was brought to r.t. and stirred at r.t. overnight (18 h). The volatiles were evaporated from the reaction mixture under reduced pressure and the white precipitate obtained was dried under vacuum for 1 h. The reaction mixture was suspended in D_2O and stirred at r.t. for five minutes to extract all the soluble

material, following which the reaction mixture was filtered over a 0.2 µm porosity filter to obtain a colorless filtrate. ^{1}H NMR analysis of the colorless filtrate revealed the presence of 2.2.2-cryptand and no discernible formation of methylated products (**Figure S35**).

Reaction of TmI$_2$ and KOSi(O^{t}Bu)$_3$ (2.0 equiv.) on a dilute small scale under dinitrogen

A colorless solution of KOSi(O^{t}Bu)$_3$ (7.2 mg, 0.02 mmol, 2.0 equiv.) in Et$_2$O (0.3 mL) at r.t. was added to a stirring brown suspension of TmI$_2$ (5.0 mg, 0.01 mmol, 1.0 equiv.) in Et$_2$O (0.3 mL) at r.t. No observable colour change was perceived during the addition. The reaction mixture was stirred vigorously at r.t. for 6 days resulting in a pale-yellow supernatant and a white/greyish white precipitate. A red-brown precipitate was observed after the first day of the reaction, which was slowly consumed during the course of the 6-day reaction period. Analysis of the ^{1}H NMR spectrum (in *d$_{12}$*-cyclohexane after the removal of volatiles) of the crude reaction mixture at the end of the 6-day reaction period revealed the formation of **1** (**Figure S2**b)) and showed that at this concentration the reaction takes 6 days for completion.
This experiment suggests that the long reaction time required for large scale synthesis is due to the heterogeneous nature of the reaction.

Reaction of TmI$_2$ and KOSi(O^{t}Bu)$_3$ (2.0 equiv.) conducted for one day on a dilute small scale under dinitrogen

A colorless solution of KOSi(O^{t}Bu)$_3$ (7.9 mg, 0.03 mmol, 2.0 equiv.) in Et$_2$O (0.3 mL) at r.t. was added to a stirring brown suspension of TmI$_2$ (5.5 mg, 0.01 mmol, 1.0 equiv.) in Et$_2$O (0.3 mL) at r.t. No observable colour change was perceived during the addition. The reaction mixture was stirred vigorously at r.t. for 1 day resulting in a pale-yellow supernatant, a white/greyish white precipitate and a red brown precipitate. Analysis of the ^{1}H NMR spectrum (in *d$_{12}$*-cyclohexane after the removal of volatiles) of the crude reaction mixture revealed the presence of **1** and an intermediate species at 5.19 ppm which is eventually consumed when the reaction is taken to completion (i.e. 6 days on a dilute small scale or 12 days on a preparative scale synthesis) (**Figure S2**a)).The same intermediate species was observed when low reaction times were used in the preparative scale syntheses.

Reaction of TmI$_2$ and KOSi(O^{t}Bu)$_3$ (3.0 equiv.) on a dilute small scale under dinitrogen

A colorless solution of KOSi(O^{t}Bu)$_3$ (11.8 mg, 0.04 mmol, 3.0 equiv.) in Et$_2$O (0.3 mL) at r.t. was added to a stirring brown suspension of TmI$_2$ (5.5 mg, 0.01 mmol, 1.0 equiv.) in Et$_2$O (0.3 mL) at r.t. No observable colour change was perceived during the addition. The reaction mixture was stirred vigorously at r.t. for 6 days resulting in a pale-yellow supernatant and a white precipitate. Analysis of the ^{1}H NMR spectrum (in *d$_{12}$*-cyclohexane after the removal of volatiles) of the crude reaction mixture at the end of the six-day reaction period revealed the formation of **1**, and unidentified species at 5.19 ppm and 34.74 (**Figure S6**).

Reaction of TmI$_2$ and KOSi(O^{t}Bu)$_3$ (2.0 equiv.) on a dilute small scale under argon

A colorless solution of KOSi(O^{t}Bu)$_3$ (7.7 mg, 0.03 mmol, 2.0 equiv.) in Et$_2$O (0.3 mL) at r.t. was added to a stirring brown suspension of TmI$_2$ (5.4 mg, 0.01 mmol, 1.0 equiv.) in Et$_2$O (0.3 mL) at r.t. No observable colour change was perceived during the addition. The reaction mixture was stirred vigorously at r.t. for 6 days resulting in a pale lime-yellow supernatant and a brownish-black precipitate. A red-brown precipitate was observed after the first day of the reaction, which was slowly consumed during the course of the 6-day reaction period. Analysis of the ^{1}H NMR spectrum (in *d$_{12}$*-cyclohexane after the removal of volatiles) of the crude reaction mixture at the end of the 6-day reaction period revealed the presence of several unidentified resonances (**Figure S8**).

Reaction of TmI$_2$ and KOSi(O^{t}Bu)$_3$ (2.0 equiv.) on a dilute small scale under argon for one day followed by stirring of the resultant reaction mixture under dinitrogen for six days

A colorless solution of KOSi(O^{t}Bu)$_3$ (8.0 mg, 0.03 mmol, 2.0 equiv.) in Et$_2$O (0.3 mL) at r.t. was added to a stirring brown suspension of TmI$_2$ (5.6 mg, 0.01 mmol, 1.0 equiv.) in Et$_2$O (0.3 mL) at r.t under argon. No observable colour change was perceived during the addition. The reaction mixture was stirred vigorously at r.t. for 1 day resulting in a pale-yellow orange supernatant, a red brown precipitate and a small amount of white precipitate. The volatiles were evaporated under vacuum, the residue was suspended in Et$_2$O (0.6 mL) and stirred vigorously at r.t. for 6 days under dinitrogen resulting in a yellow orange supernatant and a black precipitate. Analysis of the ^{1}H NMR spectrum (in *d$_{12}$*-cyclohexane after the removal of volatiles) of the crude reaction mixture at the end of the 6-day reaction period revealed the presence of **1** and unidentified species at 34.74 ppm and 7.15 ppm (**Figure S7**).

Reaction of TmI$_2$ and KOSi(O^{t}Bu)$_3$ (2.0 equiv.) in hexane under argon

A colorless solution of KOSi(O^{t}Bu)$_3$ (11.5 mg, 0.04 mmol, 2.0 equiv.) in hexane (0.6 mL) at -40 °C was added to solid TmI$_2$ (8.0 mg, 0.02 mmol, 1.0 equiv.) at -40 °C under argon. No observable colour change was perceived during the addition. The reaction mixture was stirred vigorously at -40 °C overnight (16 h) resulting in a red brown precipitate and a colorless supernatant. The volatiles were evaporated under vacuum and the reaction mixture was cooled to -80 °C. The reaction mixture at -80 °C was suspended in cold (-80 °C) *d*$_8$-THF (0.5 mL). A purple suspension formed immediately upon suspension in *d*$_8$-THF which turned to a green oil within

approximately ten seconds and a very pale green supernatant. Analysis of the ^{1}H NMR spectrum (in d_8-THF at -80 °C) of the reaction mixture revealed the presence of resonances belonging to unidentified species (**Figure S9**). Any attempts to isolate and crystallize the intermediate product formed under argon failed.

Reaction of TmI_2 and $KOSi(O^tBu)_3$ (3.0 equiv.) in hexane under dinitrogen

A colorless solution of $KOSi(O^tBu)_3$ (11.8 mg, 0.04 mmol, 3.0 equiv.) in hexane (0.6 mL) at -40 °C was added to solid TmI_2 (5.5 mg, 0.01 mmol, 1.0 equiv.) at -40 °C under dinitrogen. No observable colour change was perceived during the addition. The reaction mixture was stirred vigorously at -40 °C overnight (16 h) resulting in a red brown precipitate and a colorless supernatant. The volatiles were evaporated under vacuum and the reaction mixture was cooled to -80 °C. The reaction mixture at -80 °C was suspended in cold (-80 °C) d_8-THF (0.5 mL). A green solution formed immediately upon suspension in d_8-THF which turned to an orange suspension (i.e. the supernatant is orange) with concomitant formation of a white precipitate after stirring at -80 °C for one minute. Analysis of the ^{1}H NMR spectrum (in d_8-THF at -80 °C and -40 °C) of the reaction mixture revealed the presence of several resonances and free $KOSi(O^tBu)_3$ (**Figure S12** and **Figure S13**).

Reaction of TmI_2 and $KOSi(O^tBu)_3$ (2.0 equiv.) in d_8-THF under dinitrogen at -80 °C

A colorless solution of $KOSi(O^tBu)_3$ (8.6 mg, 0.03 mmol, 2.0 equiv.) in d_8-THF (0.4 mL) at -80 °C was added to a green solution of TmI_2 (6.0 mg, 0.01 mmol, 1.0 equiv.) in d_8-THF (0.1 mL) at -80 °C. The reaction mixture turned to an orange suspension (i.e. the supernatant is orange) during the addition with concomitant formation of a green-brown precipitate and a white precipitate. The reaction mixture was stirred at -80 °C for 3 h resulting in an orange supernatant and the continued presence of a green-brown precipitate along with a white precipitate. Analysis of the ^{1}H NMR spectrum (in d_8-THF at -80 °C and at -40 °C) revealed the presence of many resonances which were difficult to assign (**Figure S10** and **Figure S11**). Crystals of **1** and **2** could be isolated from hexane solution but due to the multiple products the complexes could not be isolated in reasonable amounts from this reaction.

Reaction of 1 with excess HCl

A cold (-80 °C) colorless solution of 2 M HCl in Et_2O (1.5 mL) was added to isolated **1** (7.4 mg, 0.003 mmol, 1.0 equiv.) at -80 °C. A pale yellow suspension was observed during the addition. The reaction mixture was brought to -40 °C and placed at -40 °C for 30 mins, following which the sample was placed at r.t. overnight (20 h). The volatiles were removed under vacuum and the sample dried under vacuum for 3 h. ^{1}H NMR spectrum of the resulting sample in d_6-DMSO with dimethylsulfone as the internal standard revealed the formation of NH_4Cl in 17% yield (0.33 equiv. of NH_4Cl per complex) (**Figure S16**).

Reaction of TmI_2 and $KOSi(O^tBu)_3$ (2.0 equiv.) under $^{15}N_2$ followed by quenching of the crude reaction mixture with excess HCl

A colorless solution of $KOSi(O^tBu)_3$ (49.7 mg, 0.16 mmol, 2.0 equiv.) in Et_2O (3.0 mL) at r.t. was added to a brown suspension of TmI_2 (34.7 mg, 0.08 mmol, 1.0 equiv.) in Et_2O (3.0 mL) at r.t. under argon. The reaction mixture was immediately brought outside the glovebox, attached to a Schlenk line, freeze/degassed (x3) and $^{15}N_2$ (700 mbar) was added whilst the reaction mixture was frozen. The resulting reaction mixture was stirred vigorously at r.t. for 12 days resulting in a pale-yellow supernatant and a white/greyish white precipitate. A red-brown precipitate was observed after the first day of the reaction, which was slowly consumed during the course of the 12-day reaction period. The reaction mixture was filtered over a porosity 4 glass filter frit to obtain a yellow solution. The volatiles were evaporated under reduced pressure and the resulting yellow residue was cooled to -80 °C. A cold (-80 °C) colorless solution of 2 M HCl in Et_2O (13.3 mL) was added to reaction mixture at -80 °C. A yellow suspension was observed during the addition. The reaction mixture was brought to -40 °C and stirred at -40 °C for 30 mins, following which the sample was stirred at r.t. overnight (20 h). The volatiles were removed under vacuum and the sample dried under vacuum for 1 h. ^{1}H NMR spectrum of the resulting sample in d_6-DMSO with dimethylsulfone as the internal standard revealed the formation of $^{15}NH_4Cl$ in 19% yield (0.38 equiv. of $^{15}NH_4Cl$ per complex; corresponding to a 100% conversion to the ^{15}N version of complex **1** according to the stoichiometry utilized with respect to the ligand)(**Figure S17**).

Reaction of 2 with excess HCl

A cold (-80 °C) colorless solution of 2 M HCl in Et_2O (1.5 mL) was added to isolated **2** (6.4 mg, 0.003 mmol, 1.0 equiv.) at -80 °C. A colorless suspension was observed during the addition. The reaction mixture was brought to -40 °C and placed at -40 °C for 30 mins, following which the sample was placed at r.t. overnight (20 h). The volatiles were removed under vacuum and the sample dried under vacuum for 3 h. ^{1}H NMR spectrum of the resulting sample in d_6-DMSO with dimethylsulfone as the internal standard revealed the formation of NH_4Cl in 18% yield (0.35 equiv. of NH_4Cl per complex) (**Figure S36**). A similar yield in NH_4Cl (17%) was also obtained for solutions of "*in situ*" prepared complex **2** (the yield was calculated considering that all complex **1** was reduced to yield complex **2**, *vide infra*).

Quenching of the crude reaction mixture obtained reacting 1 and KC_8 (5.0 equiv.) (i.e. complex 2 generated *in situ*) with excess HCl

A yellow solution of **1** (8.2 mg, 0.004 mmol, 1.0 equiv.) in Et_2O (5.0 mL) at -40 °C was added to solid KC_8 (2.5 mg, 0.019 mmol, 5.0 equiv.) at -40 °C. No observable colour change was perceived during the addition. The reaction mixture was stirred at -40 °C overnight (16 h) affording an orange supernatant along with the presence of a black precipitate (i.e. graphite) and excess bronze KC_8. The reaction mixture was filtered over a 0.2 µm porosity filter to obtain an orange filtrate. The volatiles were evaporated under reduced pressure and the resulting orange residue was cooled to -80 °C. A cold (-80 °C) colorless solution of 2 M HCl in Et_2O (1.5 mL) was added to the orange residue at -80 °C. A pale-yellow suspension was observed during the addition. The reaction mixture was brought to -40 °C and placed at -40 °C for 30 mins, following which the sample was placed at r.t. overnight (20 h). The volatiles were removed under vacuum and the sample dried under vacuum for 3 h. ^1H NMR spectrum of the resulting sample in d_6-DMSO with dimethylsulfone as the internal standard revealed the formation of NH_4Cl in 17 % yield (0.33 equiv. of NH_4Cl per complex) (**Figure S37**).

Quenching of product isolated from methylation reaction (i.e. reaction of 2 with MeOTf (1.0 equiv.)) with excess HCl

A cold (-80 °C) colorless solution of 2 M HCl in Et_2O (1.5 mL) was added to the yellow microcrystalline solid (6.4 mg) isolated from the reaction of complex **2** with MeOTf (1.0 equiv.) (*NOTE*: the yellow microcrystalline solid is a mixture of **1** and **3**). The resulting white suspension was brought to r.t. and left at r.t. overnight (16 h). The colorless supernatant was decanted and the white precipitate obtained was dried under vacuum for 1 h. ^1H NMR spectrum of the white precipitate in D_2O revealed the presence of N,N'-dimethylhydrazine dihydrochloride as the sole species (**Figure S42**). The formation of N,N'-dimethylhydrazine dihydrochloride was further confirmed by the ^1H NMR spectrum recorded after adding commercially available N,N'-dimethylhydrazine dihydrochloride (5.0 mg) to the sample in D_2O.

Quenching of the reaction mixture resulting from the reaction of 2 with MeOTf (1.0 equiv.) with excess HCl

Colorless methyl triflate (0.9 µL, 0.008 mmol, 1.0 equiv.) was added to an orange solution of isolated **2** (17.5 mg, 0.008 mmol, 1.0 equiv.) in hexane (0.8 mL) at r.t. whilst stirring. No observable colour change was perceived during the addition. The reaction mixture was stirred at r.t. for 1 h following which a yellow/lime yellow supernatant was observed along with the formation of a white precipitate (KOTf). The reaction mixture was filtered over a 0.2 µm porosity filter and the volatiles were evaporated under reduced pressure to obtain a lime yellow residue. A cold (-80 °C) colorless solution of 1 M HCl in Et_2O (8.5 mL) was added to the lime yellow residue obtained at -80 °C yielding a white suspension. The reaction mixture was brought to r.t. and stirred at r.t. overnight (18 h). The volatiles were evaporated from the reaction mixture under reduced pressure and the white precipitate obtained was dried under vacuum for 1 h. The reaction mixture was suspended in D_2O and stirred at r.t. for five minutes to extract all the soluble material, following which the reaction mixture was filtered over a 0.2 µm porosity filter to obtain a colorless filtrate. ^1H NMR analysis of the colorless filtrate revealed the presence of N,N'-dimethylhydrazine dihydrochloride in 18 % yield (dimethylsulfone as external standard)(**Figure S43**).[9,10]

Reaction of 4 with KC_8 (2.0 equiv.) in d_8-THF under dinitrogen at -80 °C

A colorless solution of **4** (8.3 mg, 0.004 mmol, 1.0 equiv.) in d_8-THF (0.5 mL) at -80 °C was added to solid KC_8 (1.2 mg, 0.009 mmol, 2.0 equiv.) at -80 °C. No observable color change was perceived during the addition. The reaction mixture was stirred at -80 °C for 3 h resulting in a colorless supernatant along with the presence of bronze KC_8. Analysis of the ^1H NMR spectrum (in d_8-THF at -80 °C and at -40 °C) of the reaction mixture revealed the continued presence of the starting material **4** and resonances corresponding to unknown species (**Figure S20** and**Figure S21**). The rection mixture was stirred at -40 °C overnight (16 h) yielding a very pale green/almost colorless supernatant and a black precipitate (i.e. graphite). Analysis of the ^1H NMR spectrum (in d_8-THF at -40 °C) revealed the complete consumption of **4** and new broad resonances at 17.68 ppm and 1.11 ppm (**Figure S21**), and the absence of a resonance at -28.43 ppm which is a characteristic signal observed upon the dissolution of **1** in d_8-THF (**Figure S4**); thereby signifying the absence of dinitrogen reduction to form **1**.

Reaction of 4 with KC_8 (2.0 equiv.) in Et_2O under dinitrogen at -40 °C

A white suspension of **4** (10.0 mg, 0.005 mmol, 1.0 equiv.) in Et_2O (0.6 mL) at -40 °C was added to solid KC_8 (1.4 mg, 0.01 mmol, 2.0 equiv.) at -40 °C. No observable color change was perceived during the addition. The reaction mixture was stirred at -40 °C for 3 days resulting in a pale-yellow supernatant and a black precipitate (i.e. graphite). Analysis of the ^1H NMR spectrum (in d_8-THF at -40 °C) of the reaction mixture after removal of the volatiles (i.e. Et_2O) revealed a broad resonance at 17.88 ppm (**Figure S22**), and the absence of a resonance at -28.43 ppm which is a characteristic signal observed upon the dissolution of **1** in d_8-THF (**Figure S4**); thereby signifying the absence of dinitrogen reduction to form **1**.

Reaction of 4 with KC_8 (2.0 equiv.) in the presence of 18-crown-6 (2.0 equiv.) in d_8-THF under dinitrogen at -80 °C

A colorless solution of **4** (8.2 mg, 0.004 mmol, 1.0 equiv.) and 18-crown-6 (2.3 mg, 0.009 mmol, 2.0 equiv.) in d_8-THF (0.5 mL) at -80 °C was added to solid KC_8 (1.2 mg, 0.009 mmol, 2.0 equiv.) at -80 °C. No observable color change was perceived during the addition. The reaction mixture was stirred at -80 °C for 3 h resulting in a colorless supernatant along with presence of bronze KC_8. Analysis of the ^{1}H NMR spectrum (in d_8-THF at -80 °C and at -40 °C) of the reaction mixture revealed the continued presence of the starting material **4** (**Figure S24** and **Figure S25**). The rection mixture was continued to stir at -40 °C overnight (16 h) yielding a colorless supernatant and a black precipitate (i.e. graphite). Analysis of the ^{1}H NMR spectrum (in d_8-THF at -40 °C) revealed the complete consumption of **4** and a new resonance at 4.14 ppm (**Figure S25**). Attempts to crystallize the underlying species formed during the reaction led to the isolation of colorless crystals of [K(18-crown-6)][Tm(OSi(O^{t}Bu)$_3$)$_4$].

Reaction of 4 with KC_8 (10.0 equiv.) in the presence of 2.2.2-cryptand (2.0 equiv.) in d_8-THF under dinitrogen at -40 °C

A colorless solution of **4** (8.0 mg, 0.004 mmol, 1.0 equiv.) and 2.2.2-cryptand (3.1 mg, 0.008 mmol, 2.0 equiv.) in d_8-THF (0.5 mL) at -40 °C was added to solid KC_8 (5.6 mg, 0.04 mmol, 10.0 equiv.) at -40 °C. The reaction mixture turned to a deep blue color a few mins after the addition following which the reaction mixture was stirred at -40 °C for 1 h resulting in a deep blue supernatant along with presence of excess bronze KC_8 and graphite. Analysis of the ^{1}H NMR spectrum (in d_8-THF at -40 °C) of the reaction mixture revealed the complete consumption of starting material and new unknown resonances (**Figure S27**). (*NOTE*: The deep blue reaction mixture turns to a pale-yellow solution upon filtration with a glass wool pipette signifying decomposition and any attempts to isolate the underlying species present proved to be futile)

B. NMR Spectroscopic Data

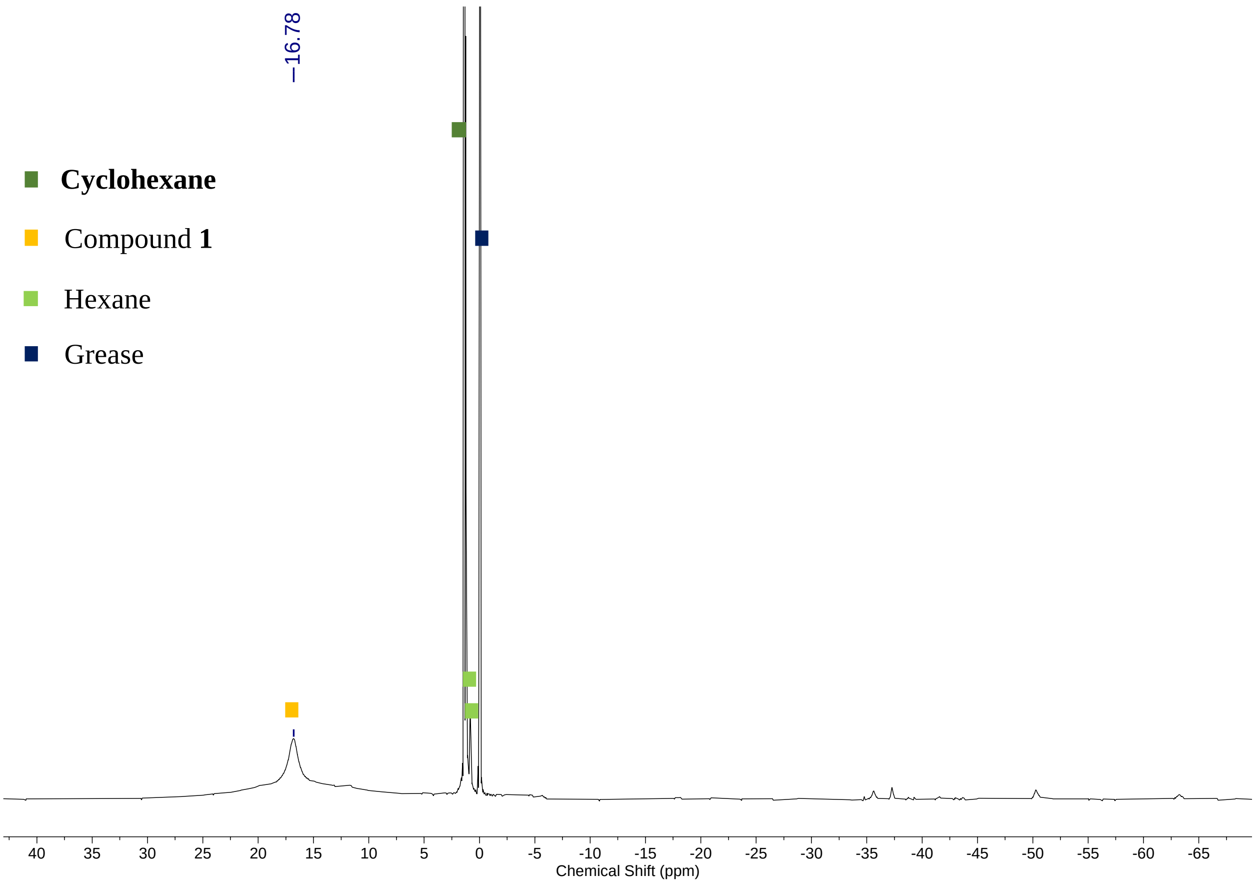

Figure S1. ^{1}H NMR spectrum (400 MHz, *d*12-cyclohexane, 298 K) of the crude reaction mixture obtained after reacting TmI_2 (500 mg) + 2 $KOSi(O^{t}Bu)_3$ under dinitrogen at r.t. for 12 days in Et_2O to yield **1**.

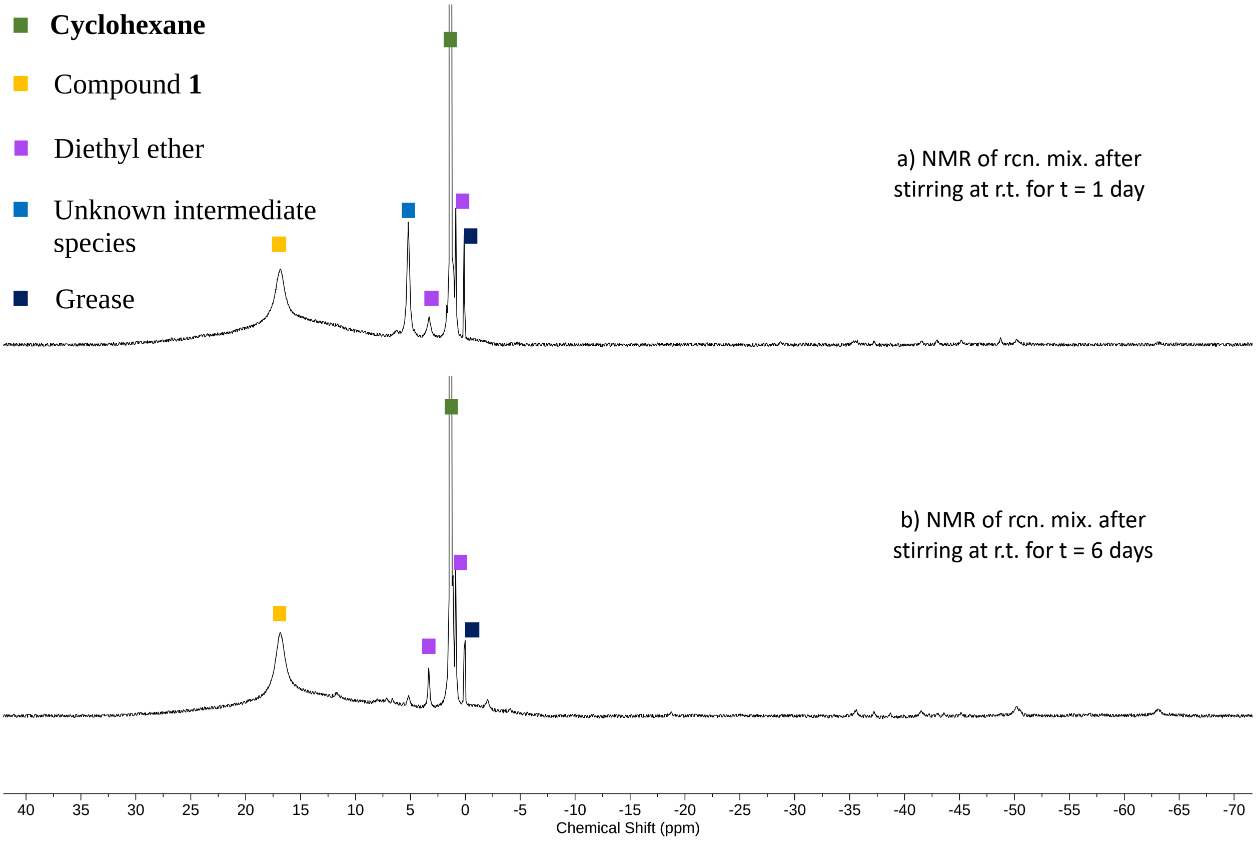

Figure S2. ^{1}H NMR spectrum (400 MHz, d_{12}-cyclohexane, 298 K) of the crude reaction mixture obtained after reacting TmI_2 (5.0 mg) + 2 $KOSi(O^tBu)_3$ conducted under dinitrogen at r.t. for a) 1 day and b) 6 days in Et_2O to yield **1**. (*NOTE*: The concentration used for this reaction was more dilute compared to the preparative scale syntheses performed).

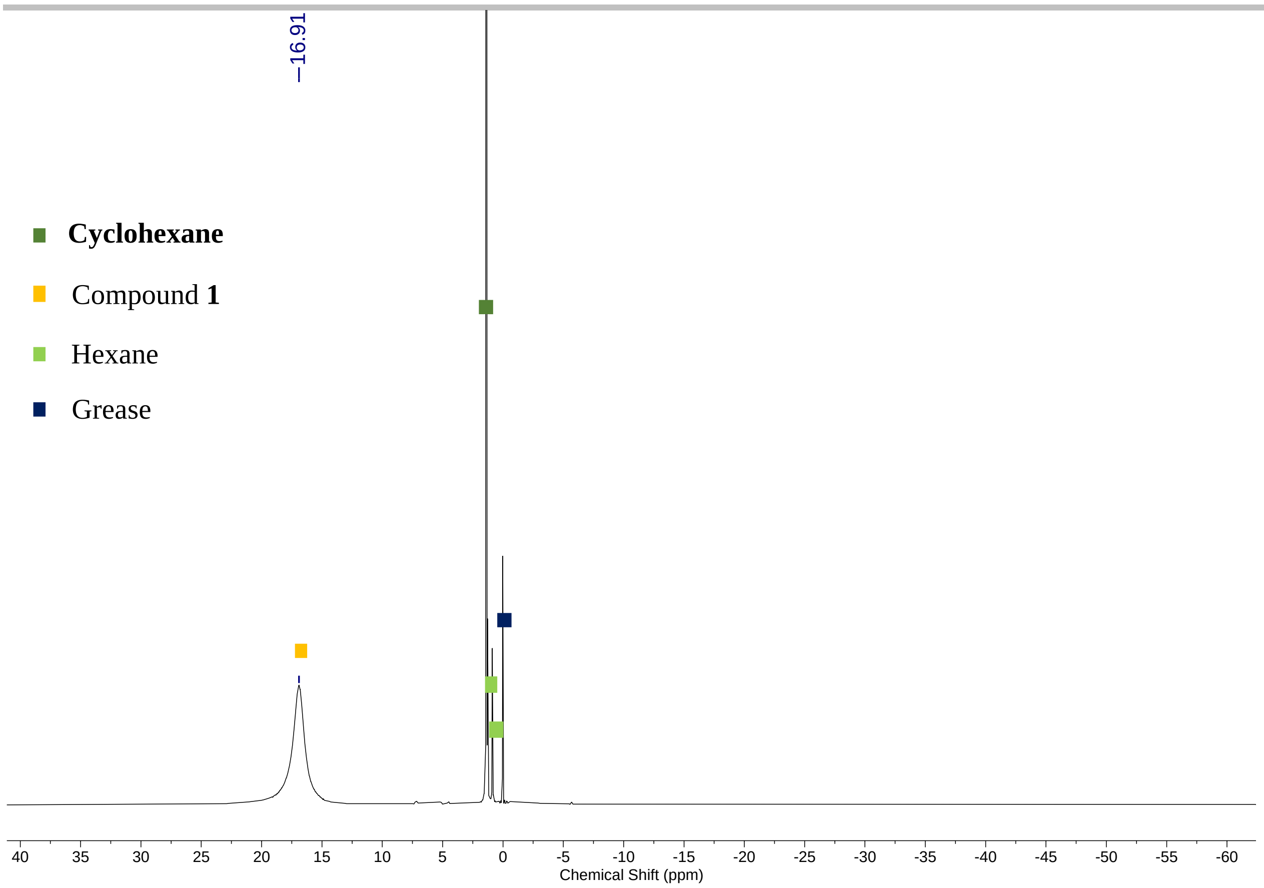

Figure S3. ^{1}H NMR spectrum (400 MHz, d_{12}-cyclohexane, 298 K) of isolated **1**.

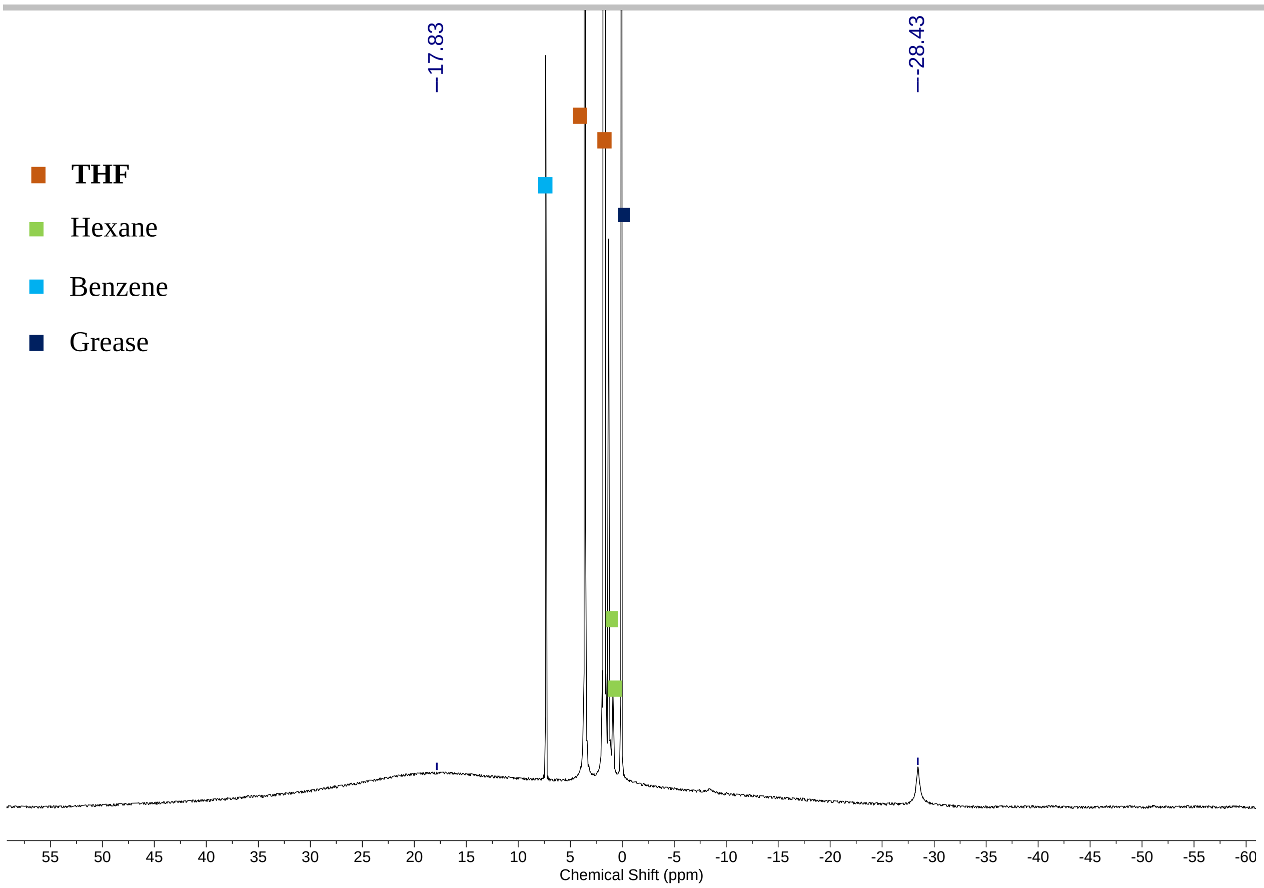

Figure S4. ^{1}H NMR spectrum (400 MHz, d_8-THF, 233 K) of isolated **1**.

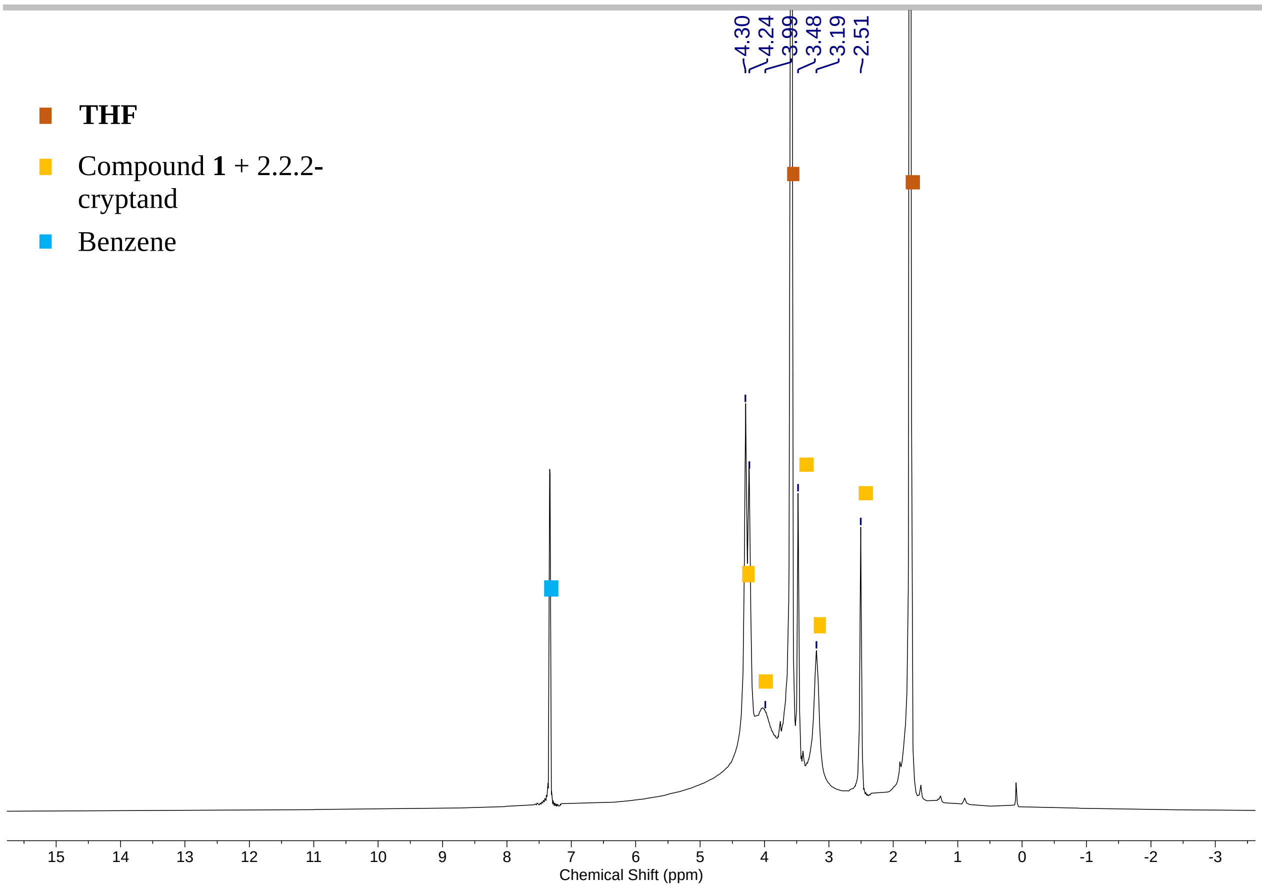

Figure S5. ^{1}H NMR spectrum (400 MHz, d_8-THF, 233 K) of the resulting reaction mixture obtained upon the addition of a solution of 2.2.2-cryptand (2.0 equiv.) in d_8-THF to a solution of isolated **1** in d_8-THF (*NOTE*: the addition of the reagents was performed at -40 °C)

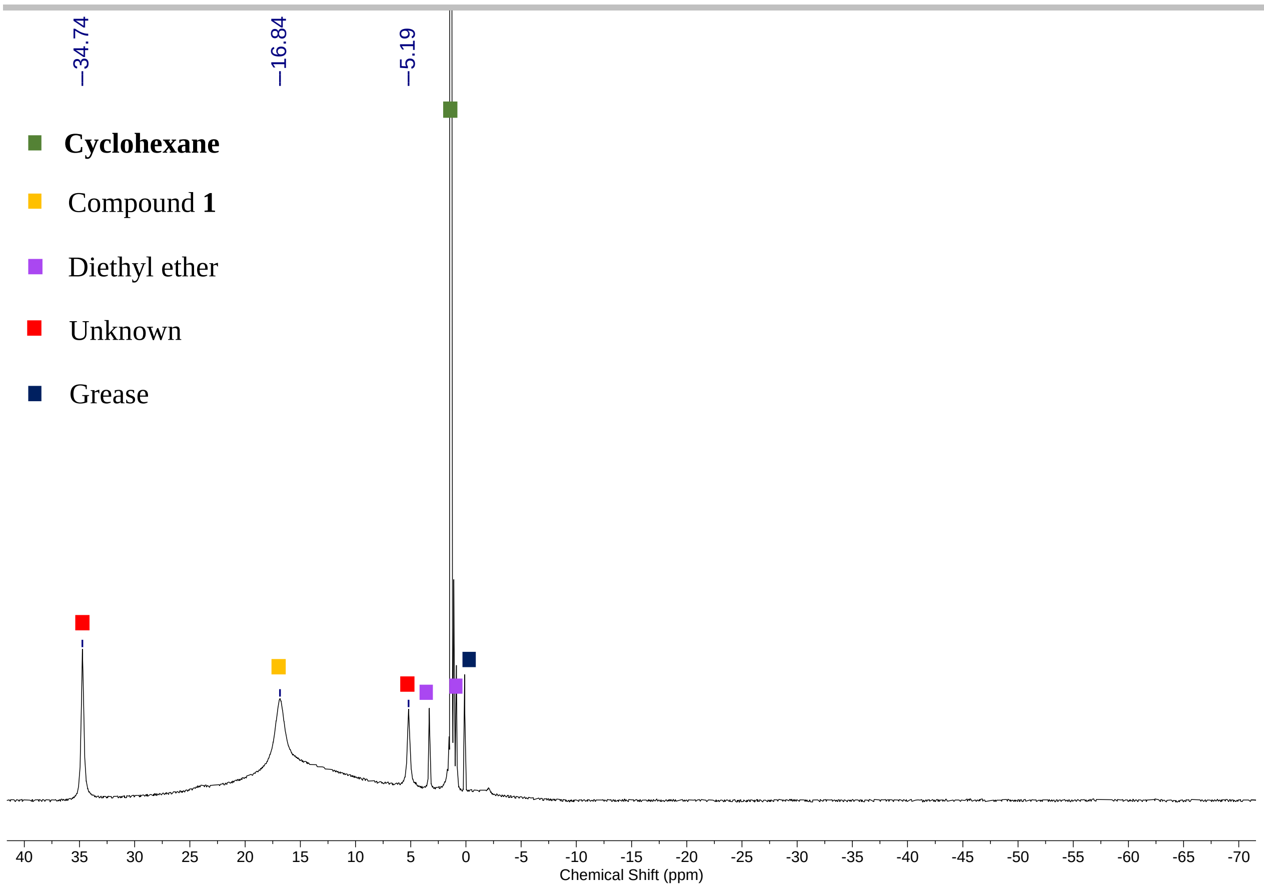

Figure S6. ^{1}H NMR spectrum (400 MHz, *d*12-cyclohexane, 298 K) of the crude reaction mixture obtained from TmI_2 + 3 $KOSi(O^tBu)_3$ conducted under dinitrogen at r.t. for 6 days in Et_2O.

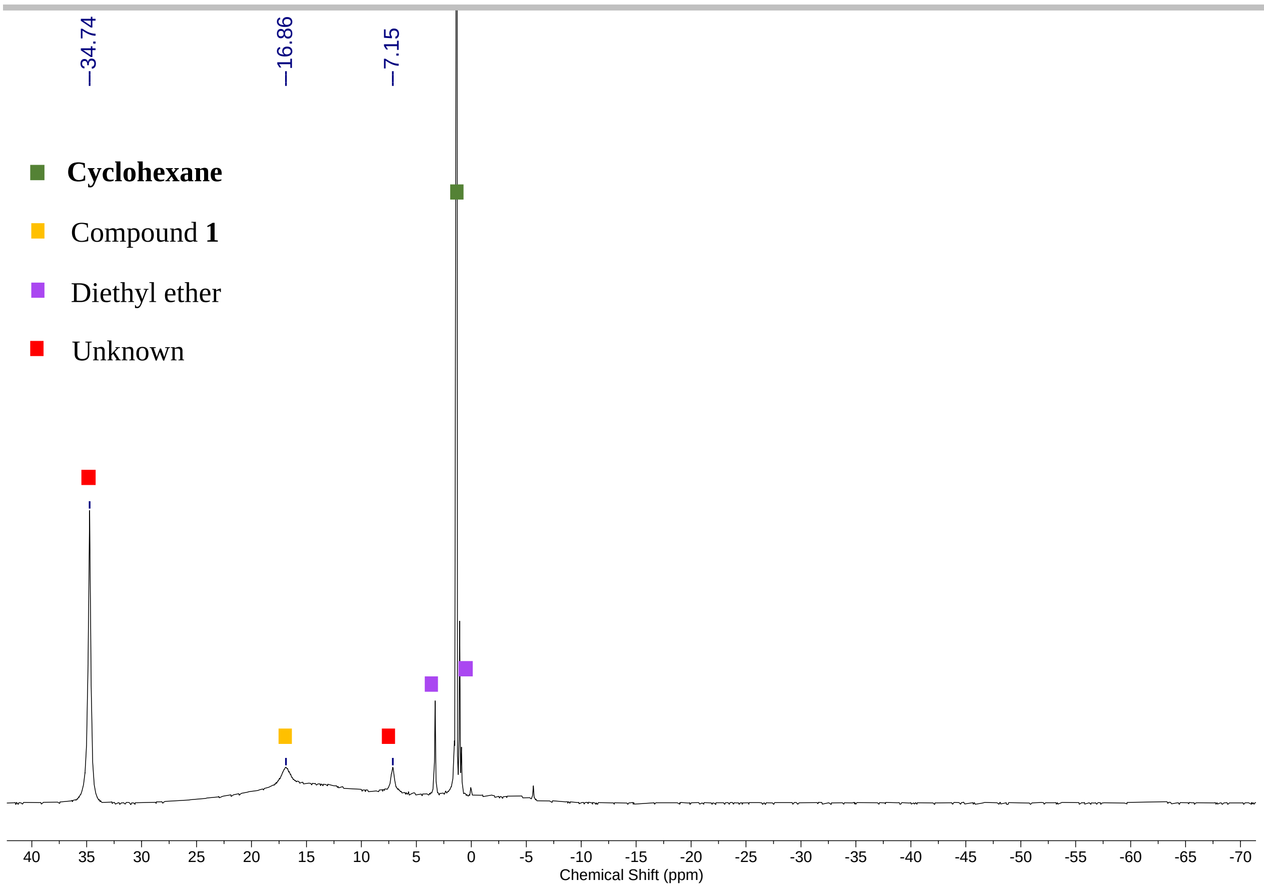

Figure S7. ^{1}H NMR spectrum (400 MHz, d_{12}-cyclohexane, 298 K) of the crude reaction mixture obtained when the TmI_2 + 2 $KOSi(O^tBu)_3$ reaction conducted under argon at r.t. for 1 day in Et_2O was stirred under dinitrogen at r.t. for 6 days in Et_2O.

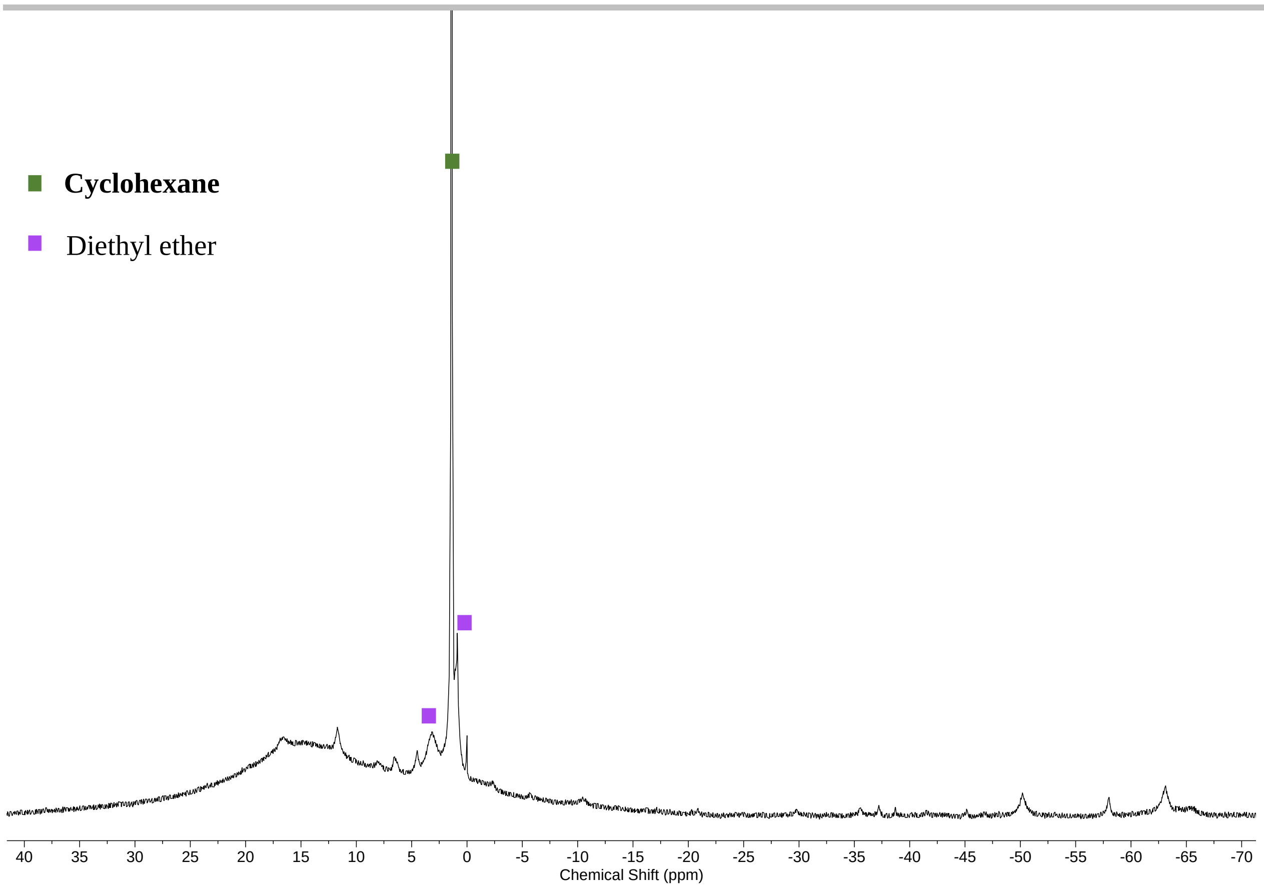

Figure S8. ^{1}H NMR spectrum (400 MHz, d_{12}-cyclohexane, 298 K) of the crude reaction mixture obtained after reacting TmI_2 (5.4 mg) + 2 $KOSi(O^{t}Bu)_3$ conducted under argon at r.t. for 6 days in Et_2O.

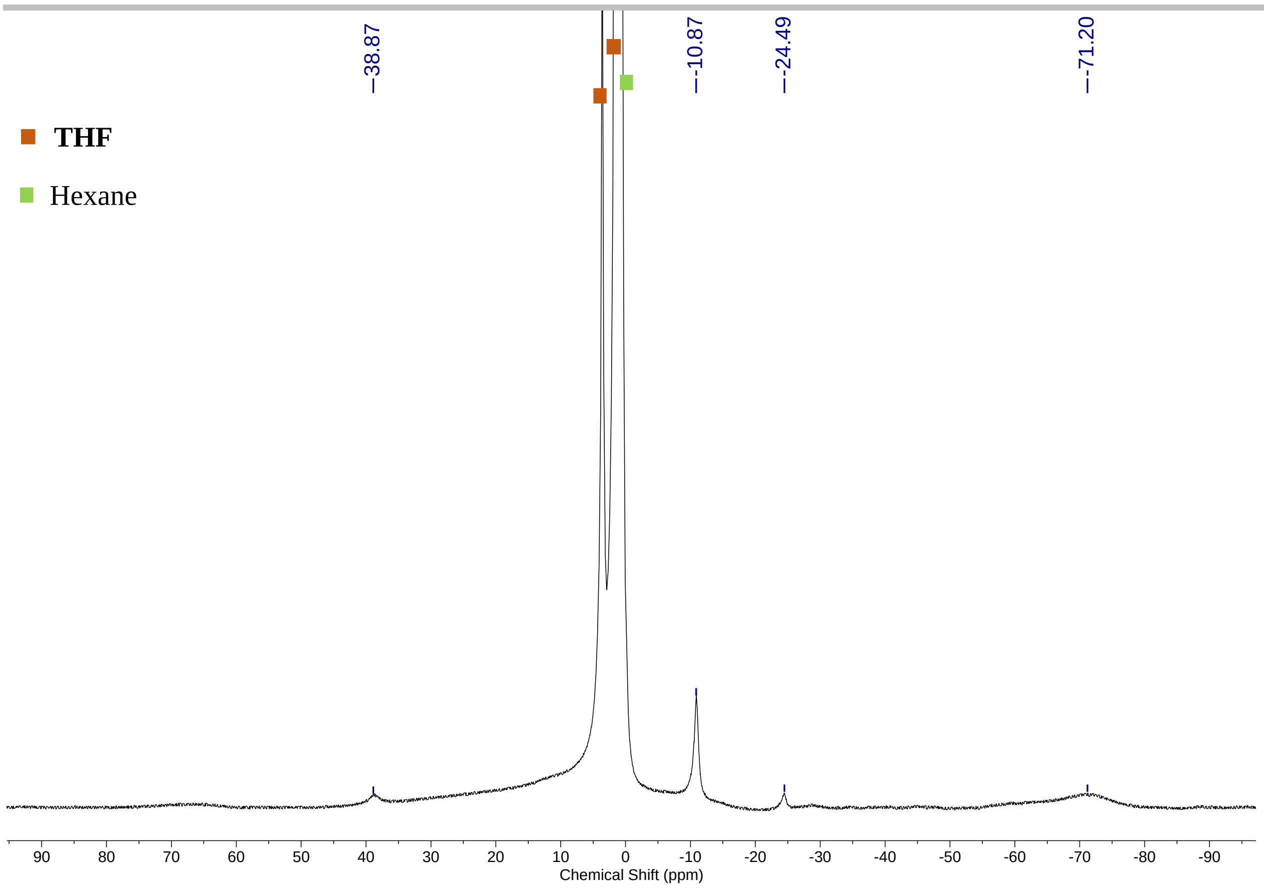

Figure S9. ^{1}H NMR spectrum (400 MHz, d_8-THF, 193 K) of the crude reaction mixture obtained when the TmI_2 + 2 $KOSi(O^tBu)_3$ reaction conducted under argon at -40 °C for 16 h in hexane is suspended in cold (-80 °C) d_8-THF (*NOTE*: Upon suspending the red brown precipitate formed from the reaction in hexane, a green oil forms immediately with a very pale green supernatant).

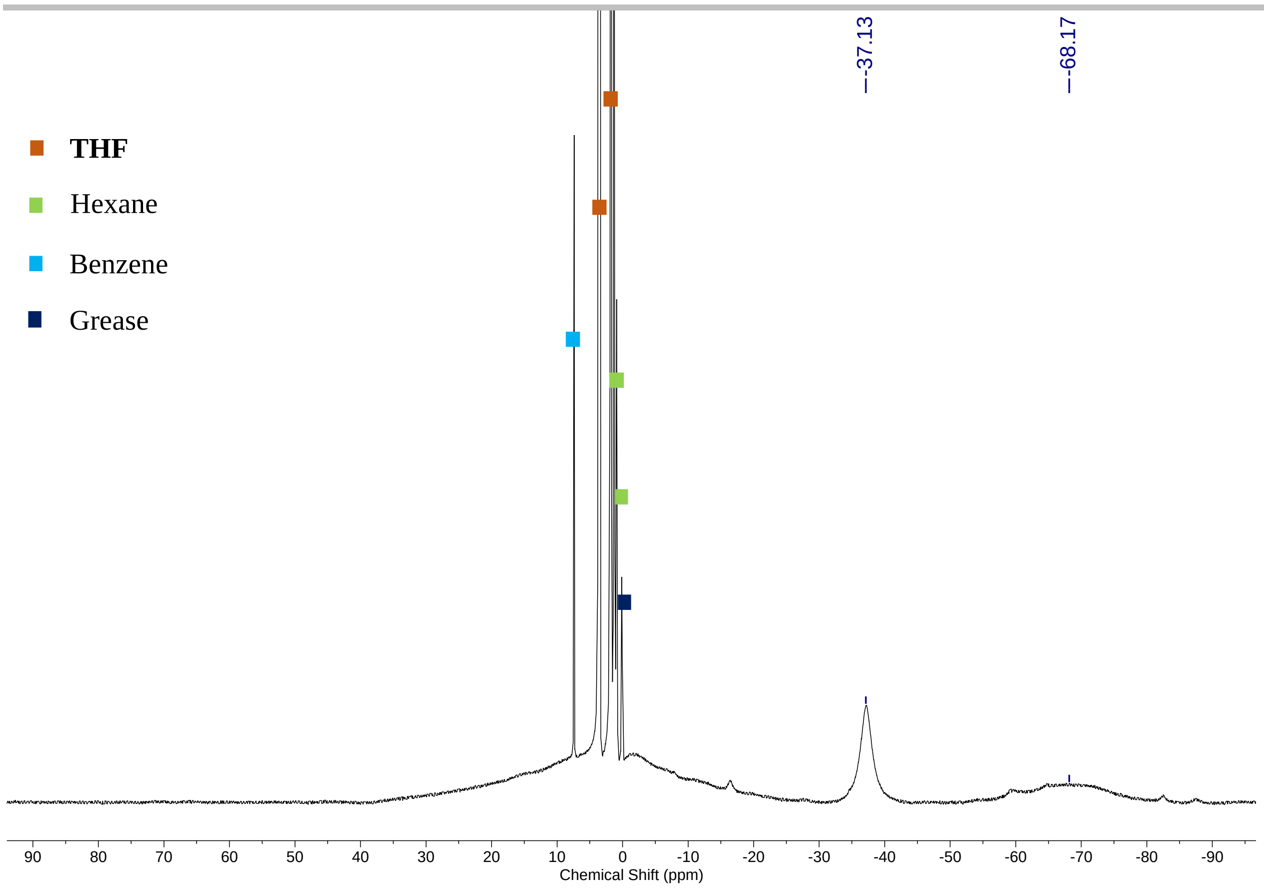

Figure S10. ^{1}H NMR spectrum (400 MHz, d_8-THF, 193 K) of the crude reaction mixture obtained from reacting TmI_2 with 2 $KOSi(O^tBu)_3$ under dinitrogen at -80 °C in d_8-THF.

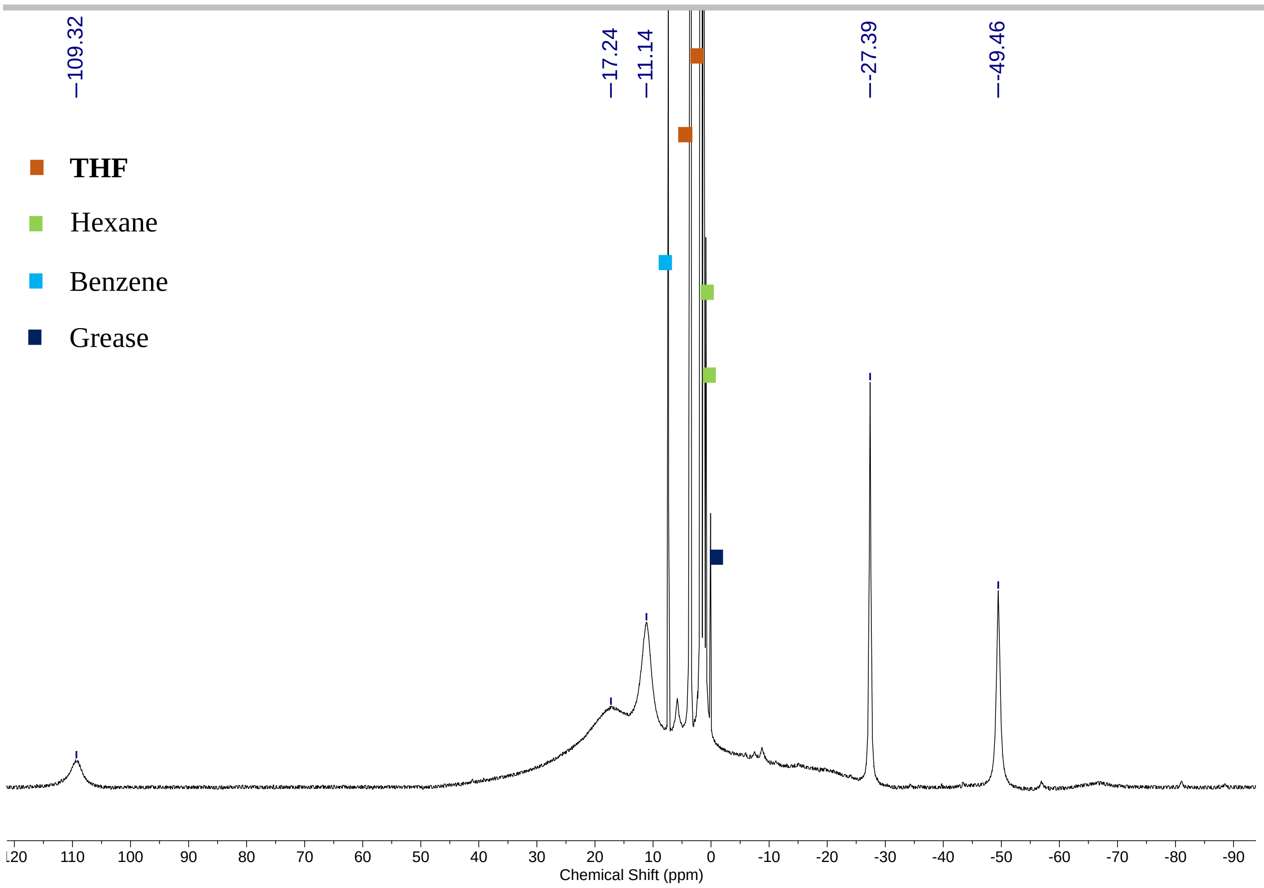

Figure S11. ^{1}H NMR spectrum (400 MHz, d_8-THF, 233 K) of the crude reaction mixture obtained from TmI_2 + 2 $KOSi(O^tBu)_3$ conducted under dinitrogen at -80 °C in d_8-THF.

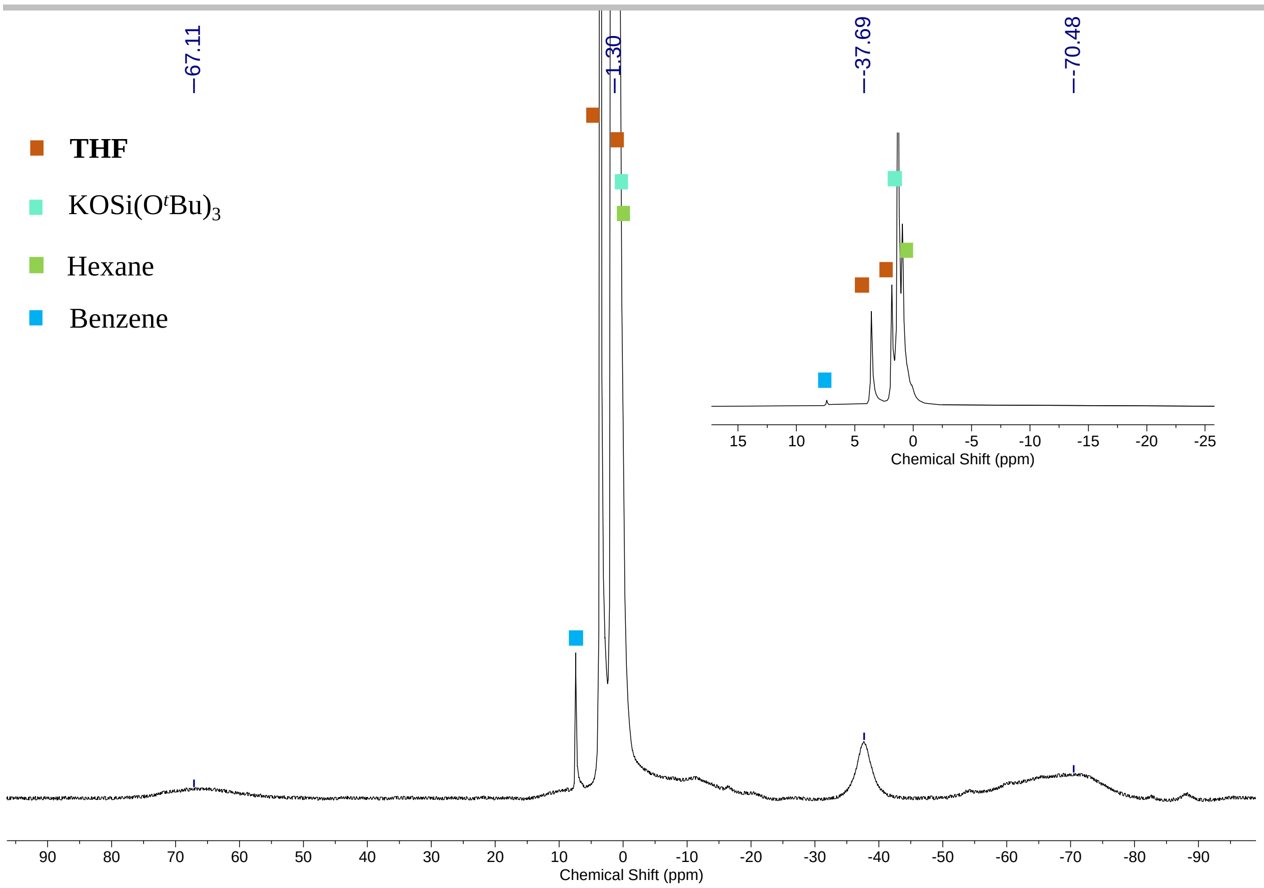

Figure S12. ^{1}H NMR spectrum (400 MHz, d_8-THF, 193 K) of the crude reaction mixture obtained when the TmI_2 + 3 $KOSi(O^tBu)_3$ reaction conducted under dinitrogen at -40 °C for 16 h in hexane is suspended in cold (-80 °C) d_8-THF.

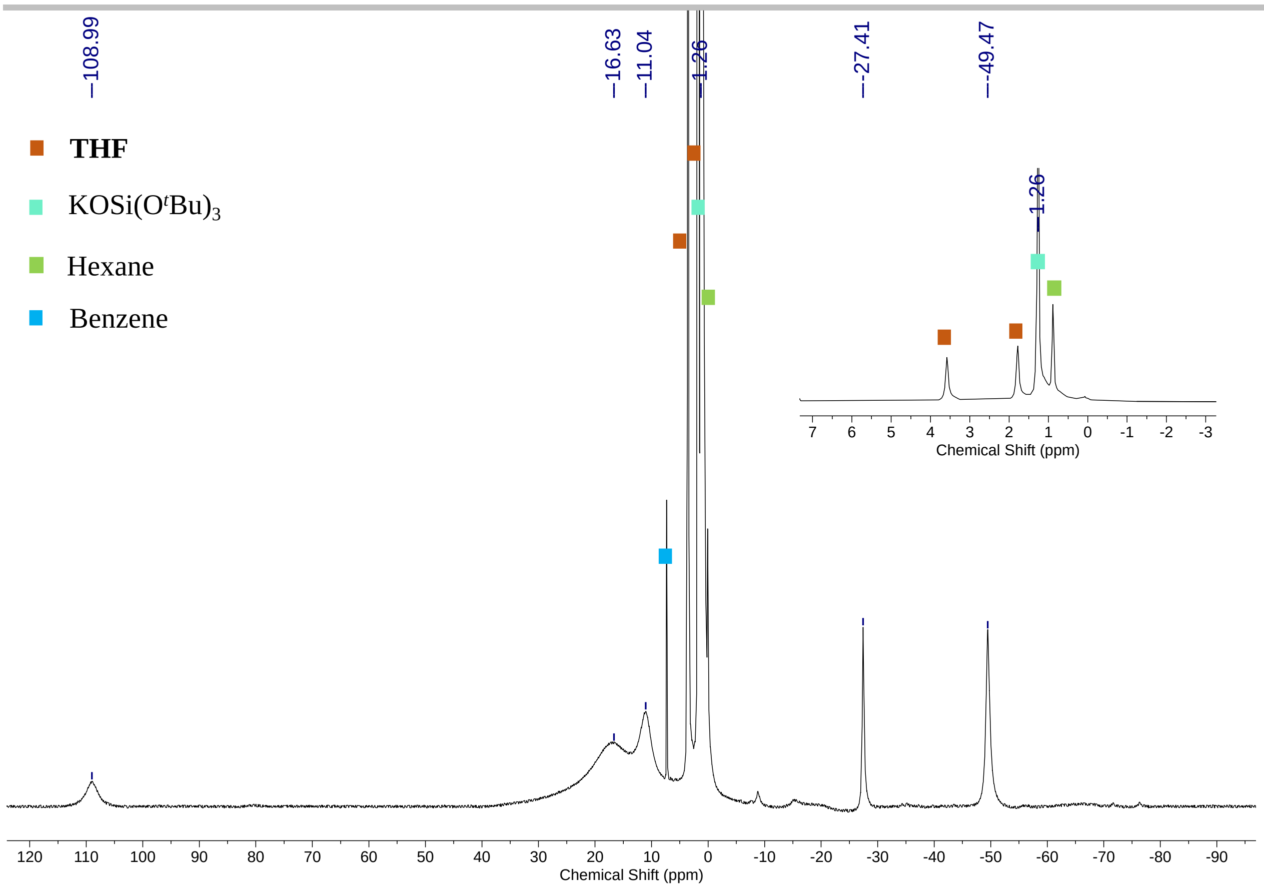

Figure S13. ^{1}H NMR spectrum (400 MHz, d_8-THF, 233 K) of the crude reaction mixture obtained when the TmI_2 + 3 KOSi(O^{t}Bu)$_3$ reaction conducted under dinitrogen at -40 °C for 16 h in hexane is suspended in cold (-80 °C) d_8-THF.

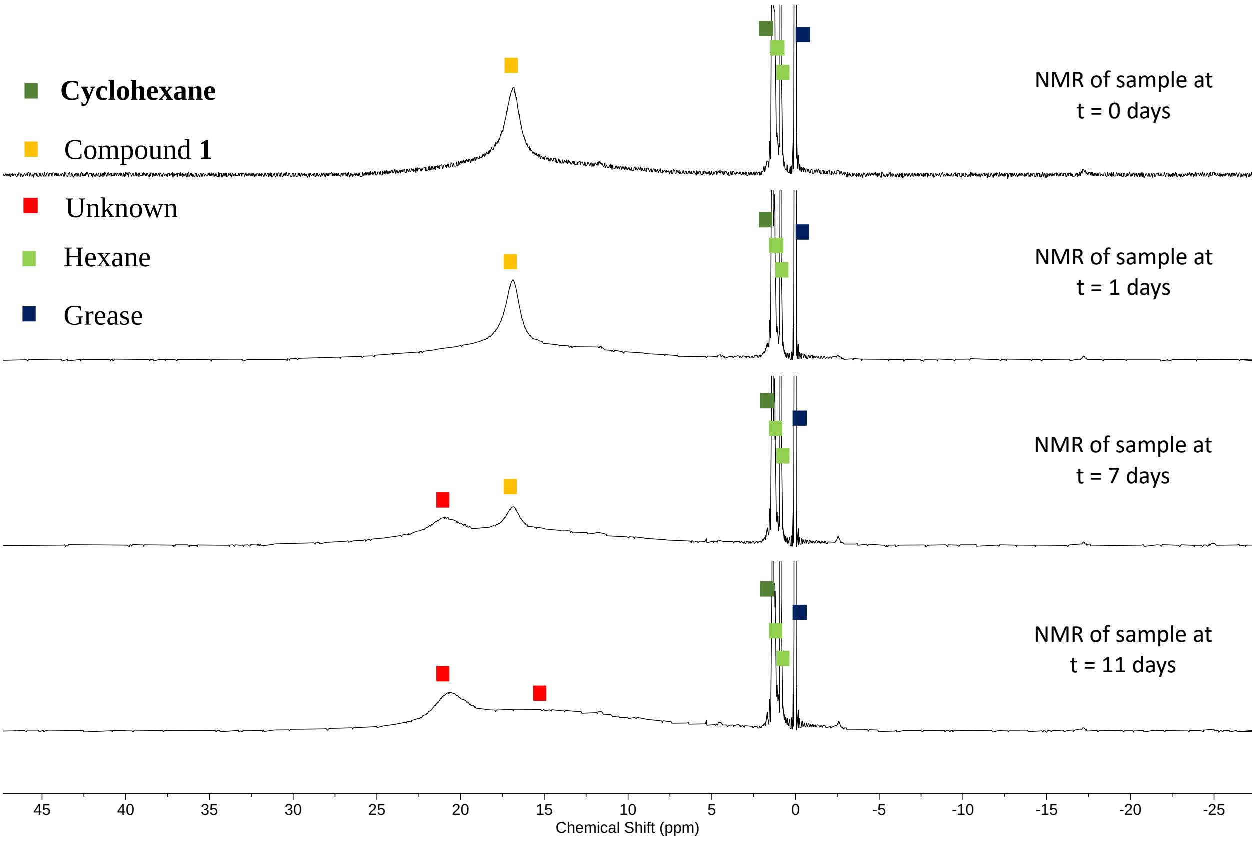

Figure S14. Evolution of the ^{1}H NMR spectrum (400 MHz, *d*12-cyclohexane, 298 K) of a cyclohexane solution of **1** after dissolution (sample was stored at r.t. at all times) under N_2.

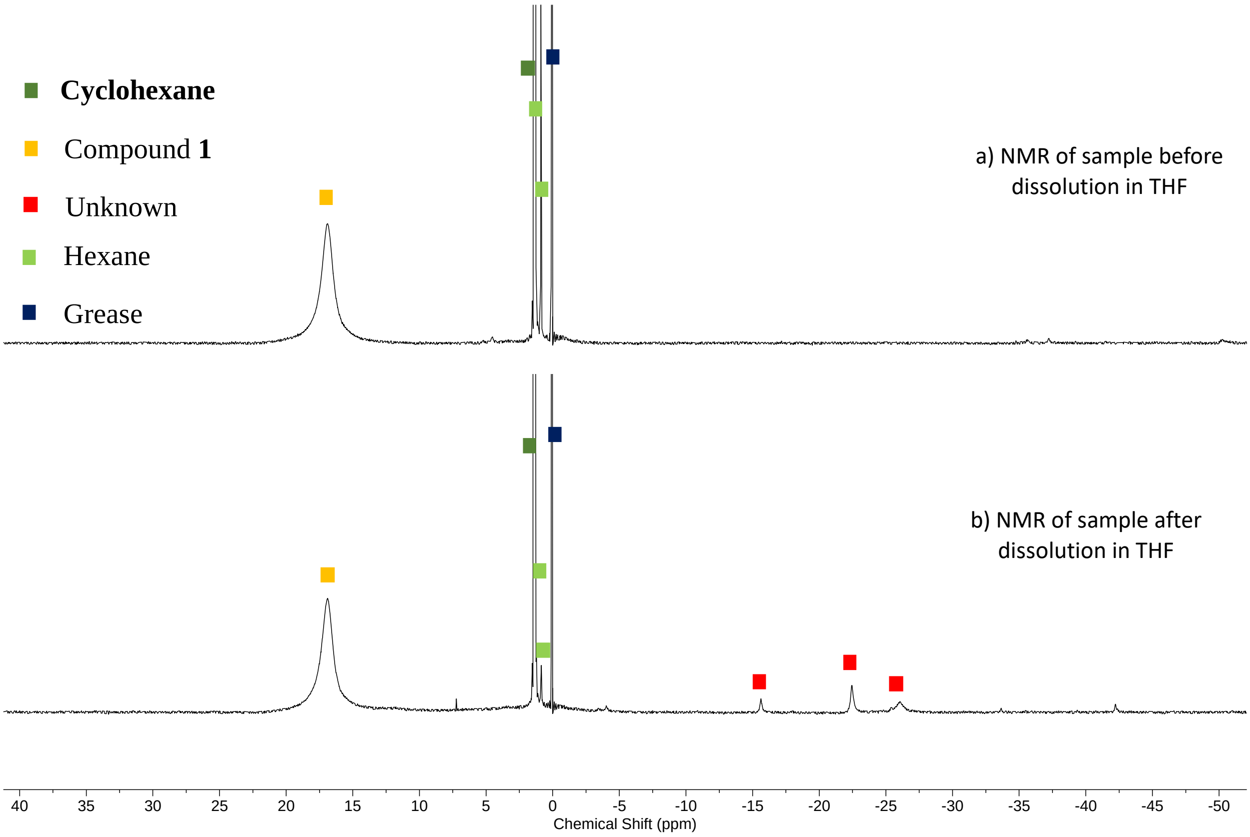

Figure S15. ^{1}H NMR spectrum (400 MHz, d_{12}-cyclohexane, 298 K) of a) isolated **1** and b) isolated **1** immediately after dissolution in THF at -40 °C and removal of the volatiles.

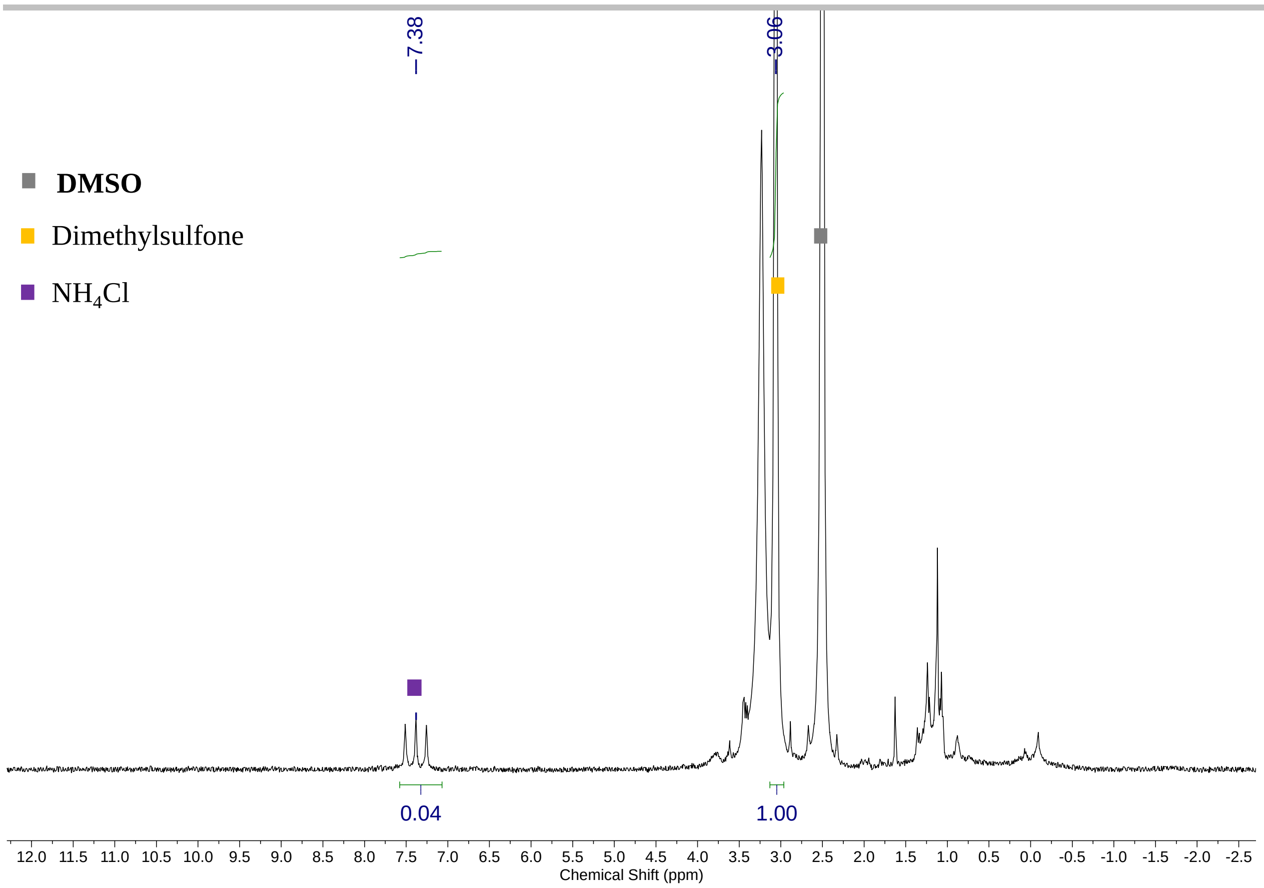

Figure S16. ^{1}H NMR spectrum (400 MHz, d_6-DMSO, 298 K) of the residue obtained after the evaporation of the reaction mixture obtained from the addition of excess 2M HCl (Et_2O) to isolated complex **1**.

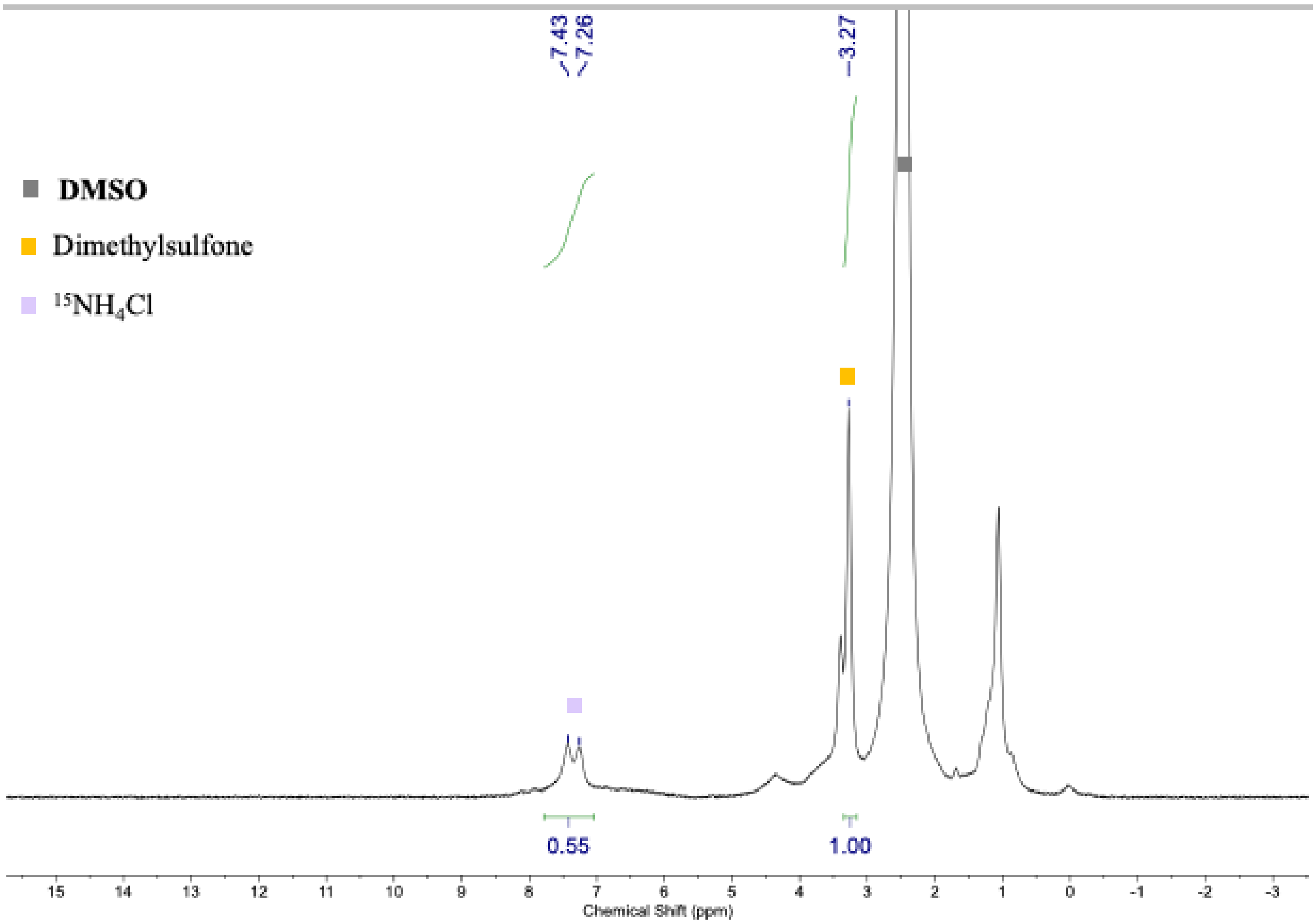

Figure S17. ^{1}H NMR spectrum (400 MHz, d_6-DMSO, 298 K) of the residue obtained after the evaporation of the reaction mixture obtained from the addition of excess 2M HCl (Et_2O) to the crude reaction mixture obtained by reacting TmI_2 and 2 $KOSi(O^tBu)_3$ under $^{15}N_2$ in diethyl ether for 12 days.

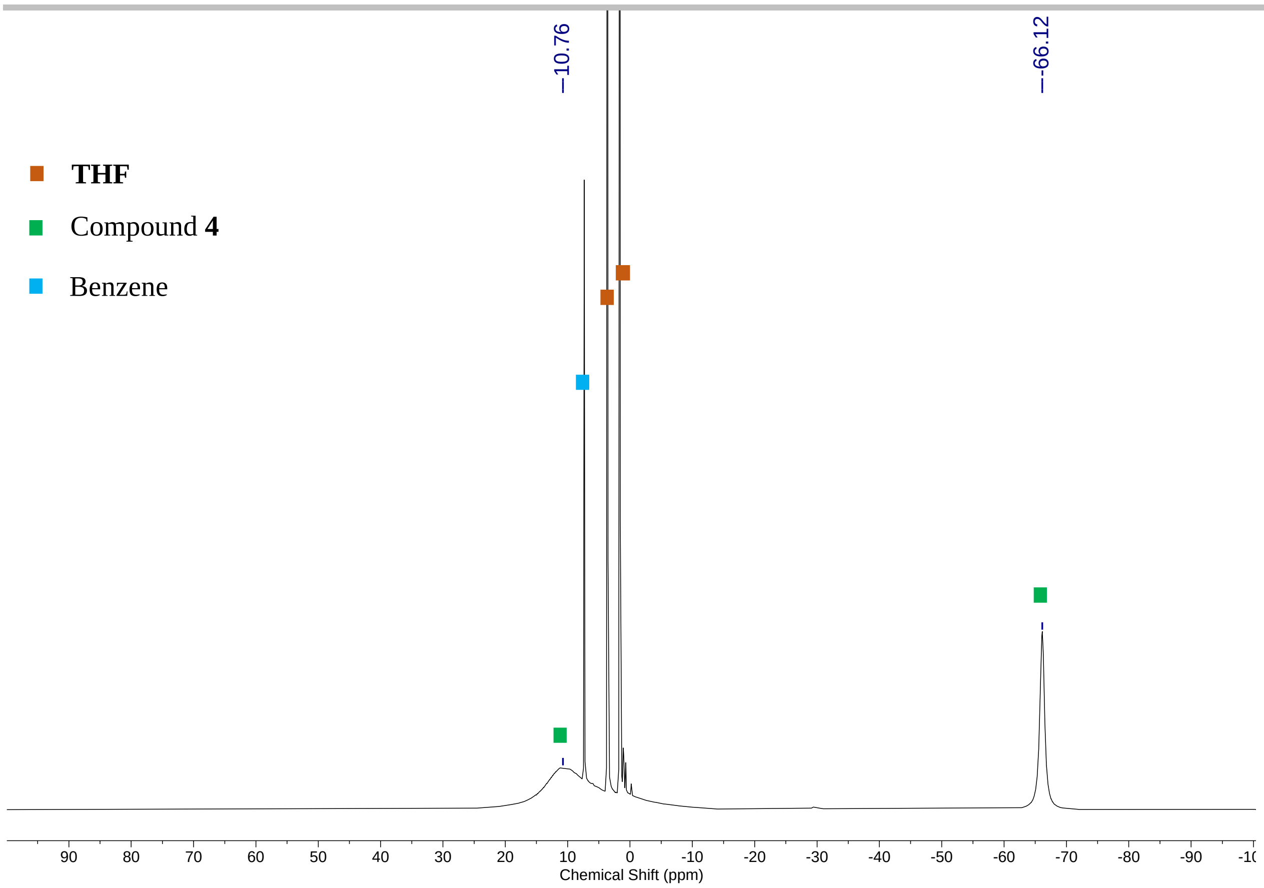

Figure S18. ^{1}H NMR spectrum (400 MHz, d_8-THF, 193 K) of isolated **4**.

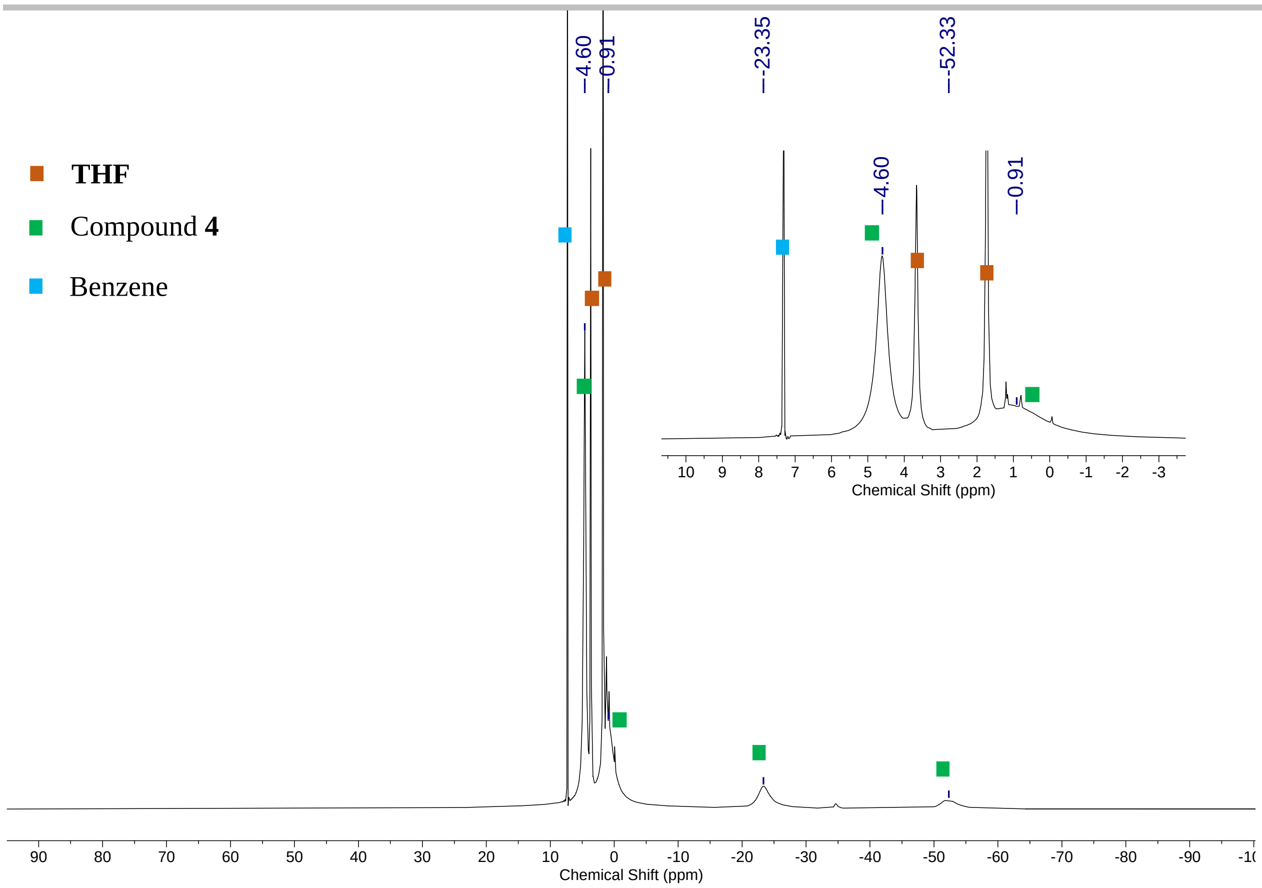

Figure S19. ^{1}H NMR spectrum (400 MHz, d_8-THF, 233 K) of isolated **4**.

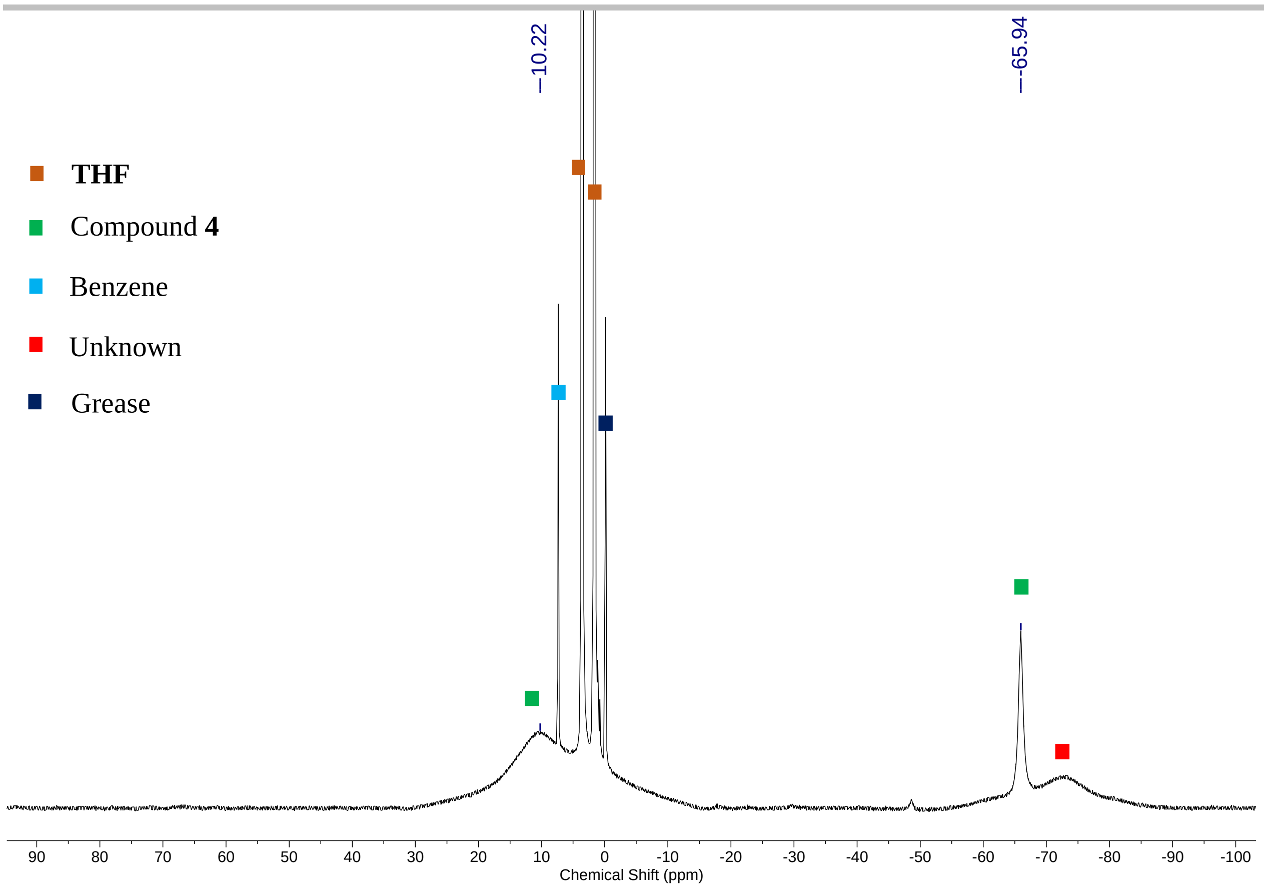

Figure S20. ^{1}H NMR spectrum (400 MHz, d_8-THF, 193 K) of the reaction mixture obtained by reacting **4** with 2.0 equiv. KC_8 in d_8-THF under dinitrogen at -80 °C for 3 h.

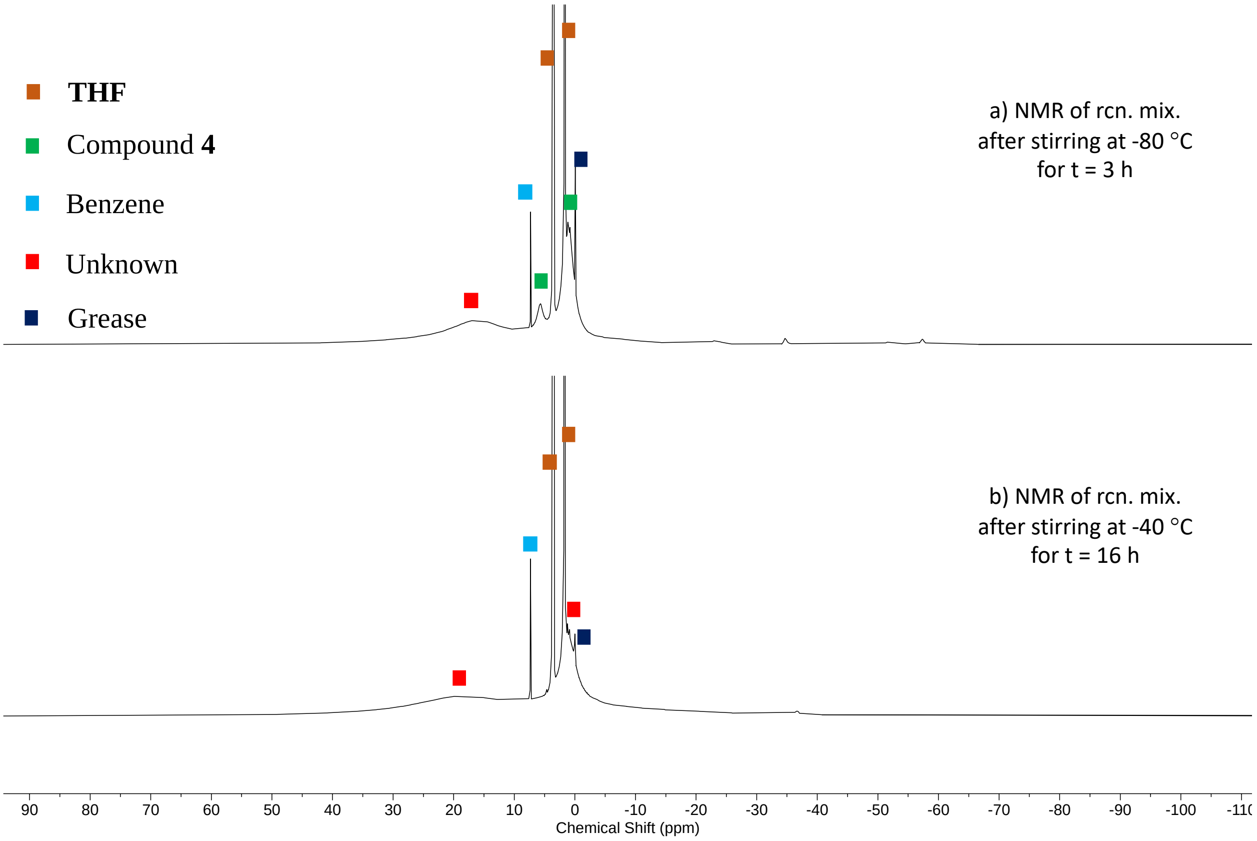

Figure S21. ^{1}H NMR spectrum (400 MHz, d_8-THF, 233 K) of a) the reaction mixture obtained by reacting **4** with 2.0 equiv. KC_8 in d_8-THF under dinitrogen at -80 °C for 3 h followed by b) the reaction mixture obtained after stirring the original reaction mixture at -40 °C overnight (16 h) after raising its temperature from -80 °C to -40 °C.

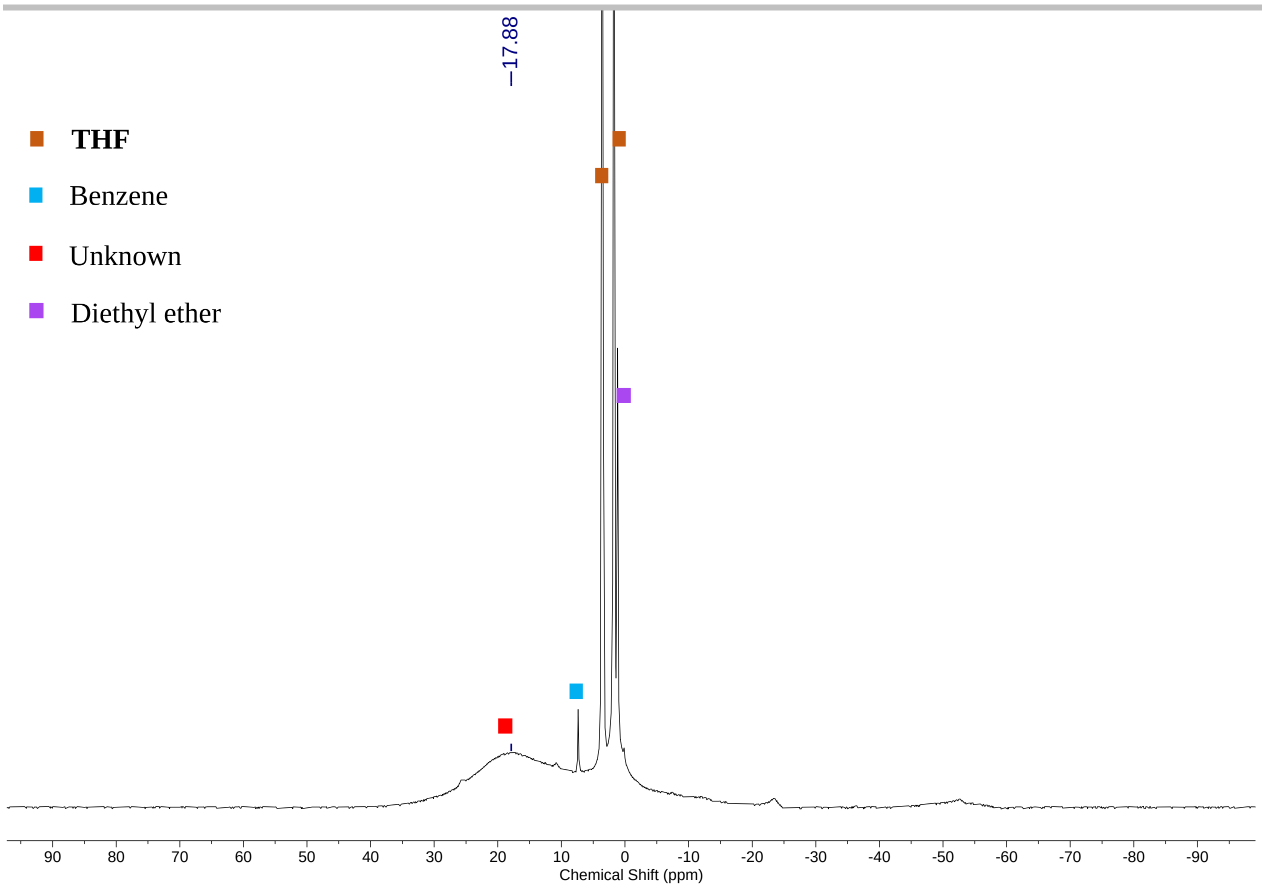

Figure S22. ^{1}H NMR spectrum (400 MHz, d_8-THF, 233 K) of the reaction mixture obtained by reacting **4** with 2.0 equiv. KC_8 in Et_2O under dinitrogen at -40 °C for 3 days.

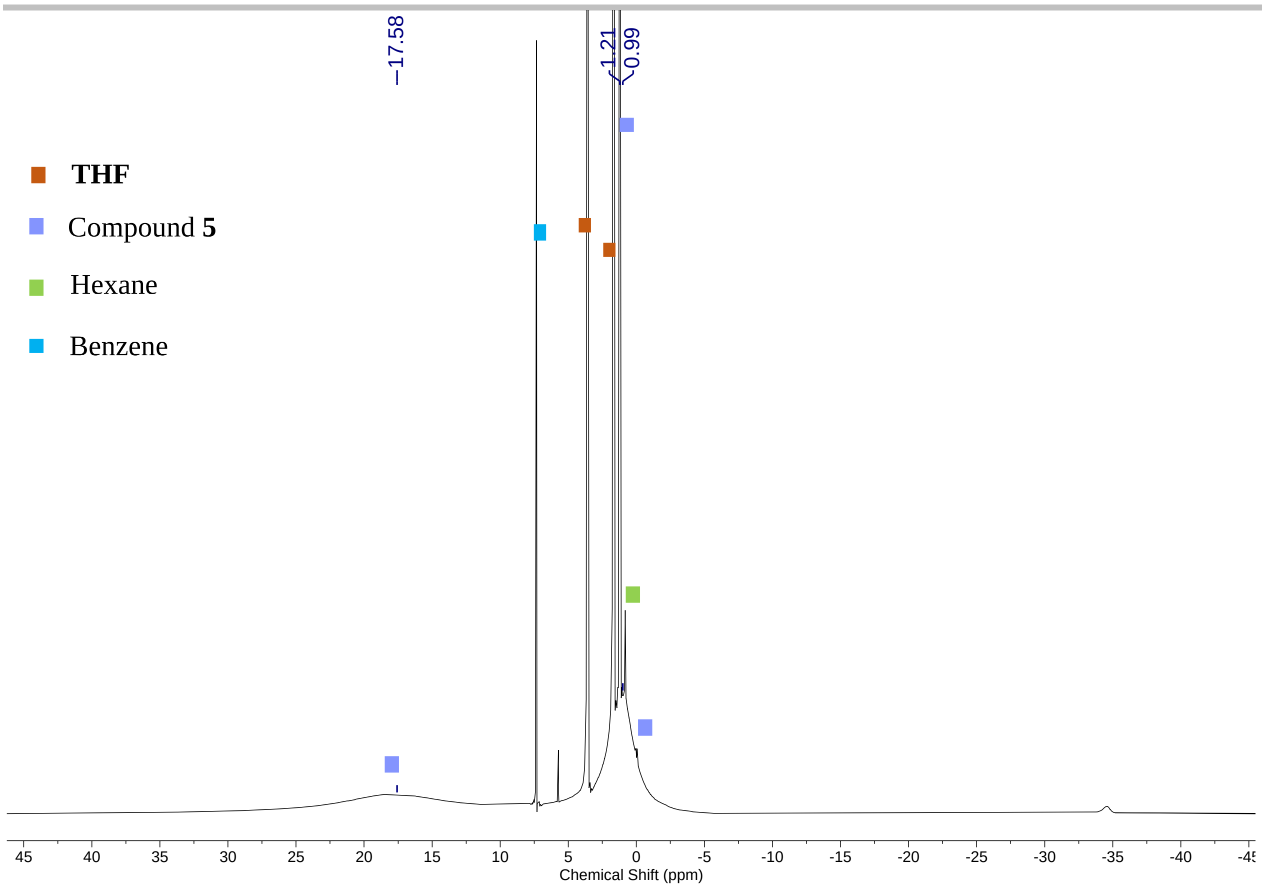

Figure S23. ^{1}H NMR spectrum (400 MHz, d_8-THF, 233 K) of $[KTm(OSi(O^tBu)_3)_4]$ (**5**).

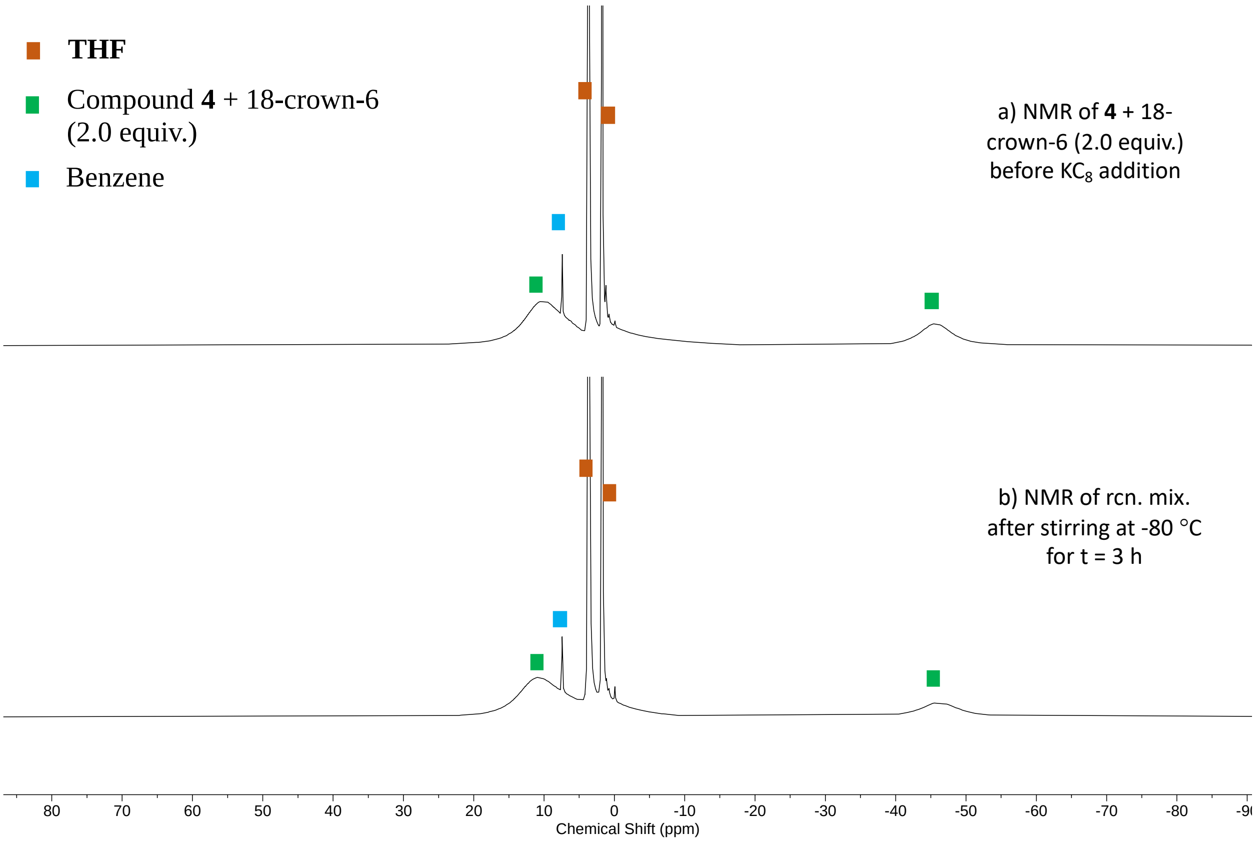

Figure S24. ^{1}H NMR spectrum (400 MHz, d_8-THF, 193 K) of a) **4** and 2.0 equiv. 18-crown-6 and b) the reaction mixture obtained by reacting **4** and 2.0 equiv. 18-crown-6 with 2.0 equiv. KC_8 in d_8-THF under dinitrogen at -80 °C for 3 h.

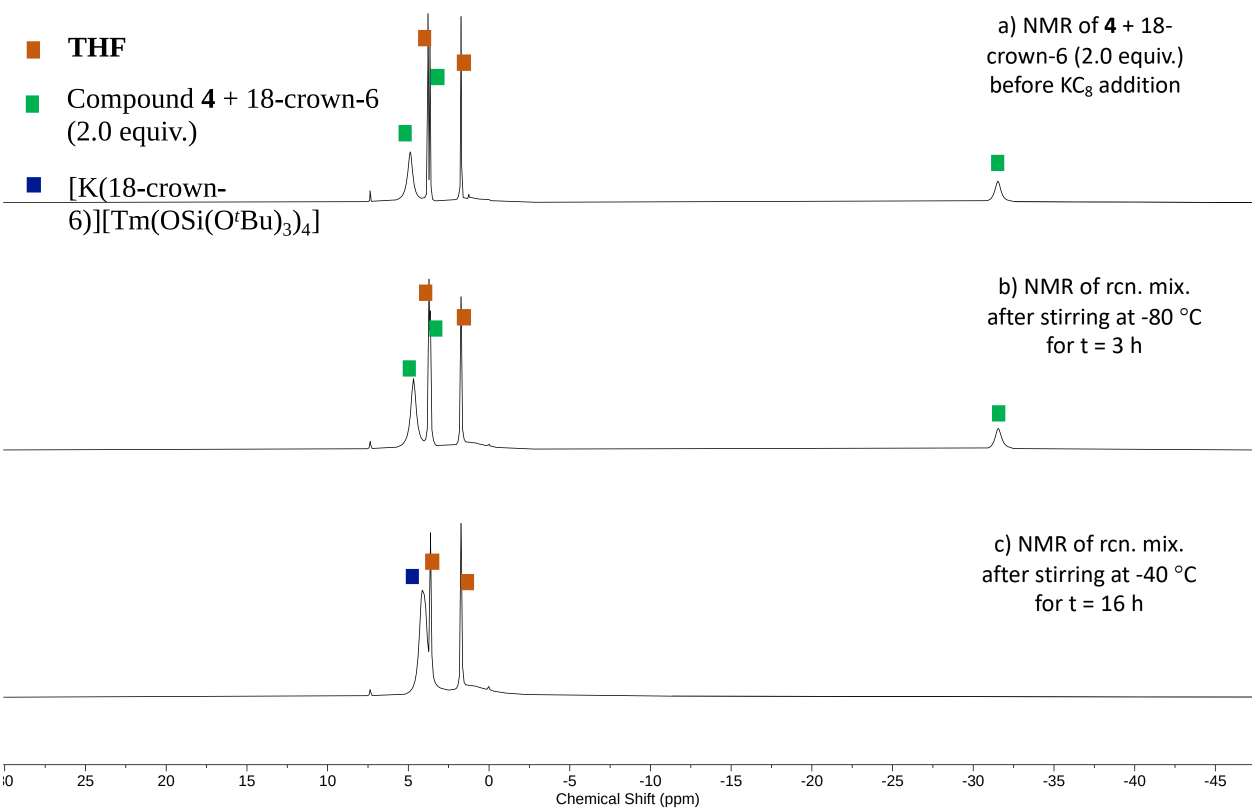

Figure S25. ^{1}H NMR spectrum (400 MHz, d_8-THF, 233 K) of a) **4** and 2.0 equiv. 18-crown-6, b) the reaction mixture obtained by reacting **4** and 2.0 equiv. 18-crown-6 with 2.0 equiv. KC_8 in d_8-THF under dinitrogen at -80 °C for 3 h and c) the reaction mixture obtained after stirring the original reaction mixture at -40 °C overnight (16 h) after raising its temperature from -80 °C to -40 °C.

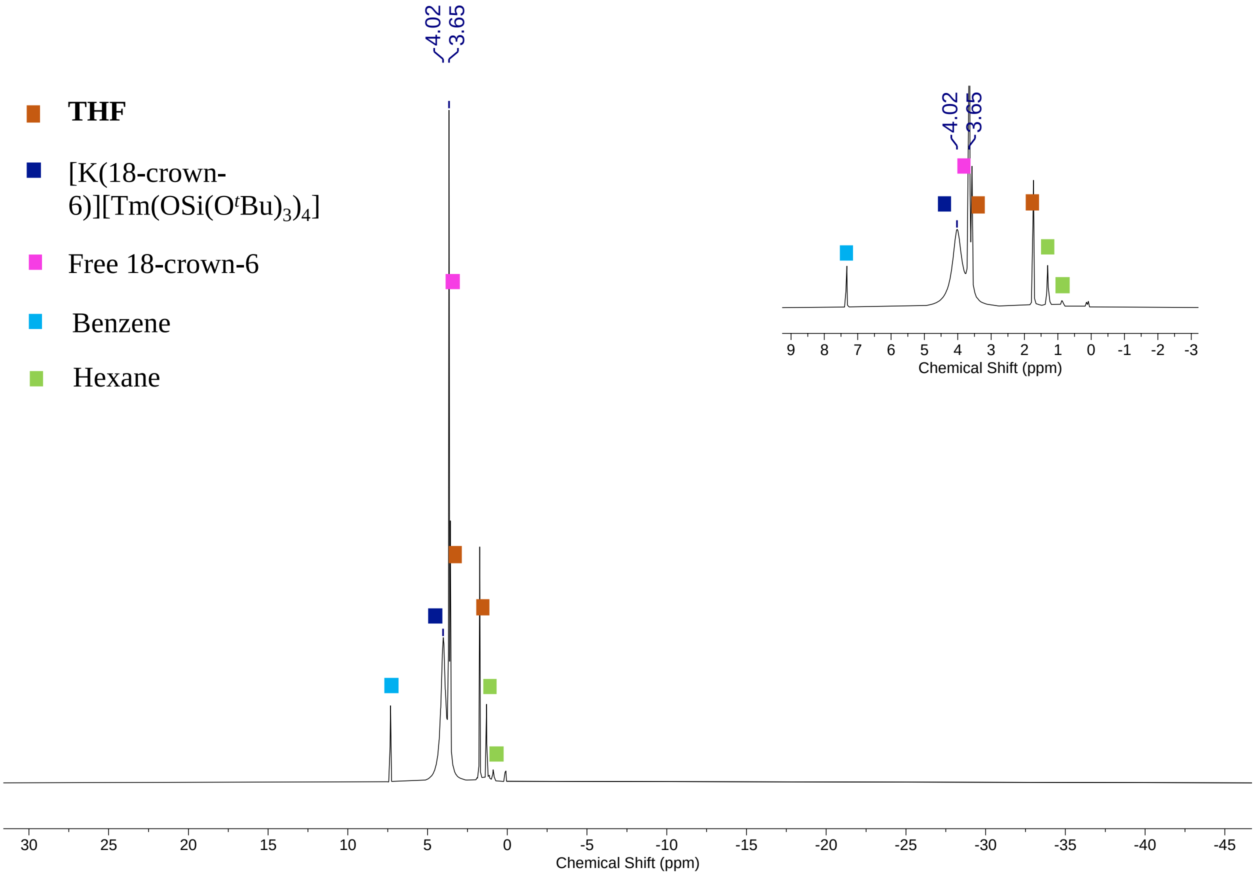

Figure S26. ^{1}H NMR spectrum (400 MHz, d_8-THF, 233 K) of the resulting reaction mixture when a solution of 18-crown-6 (2.0 equiv.) in d_8-THF is added to a solution of [KTm(OSi(O^{t}Bu)$_3$)$_4$] (**5**) in d_8-THF.

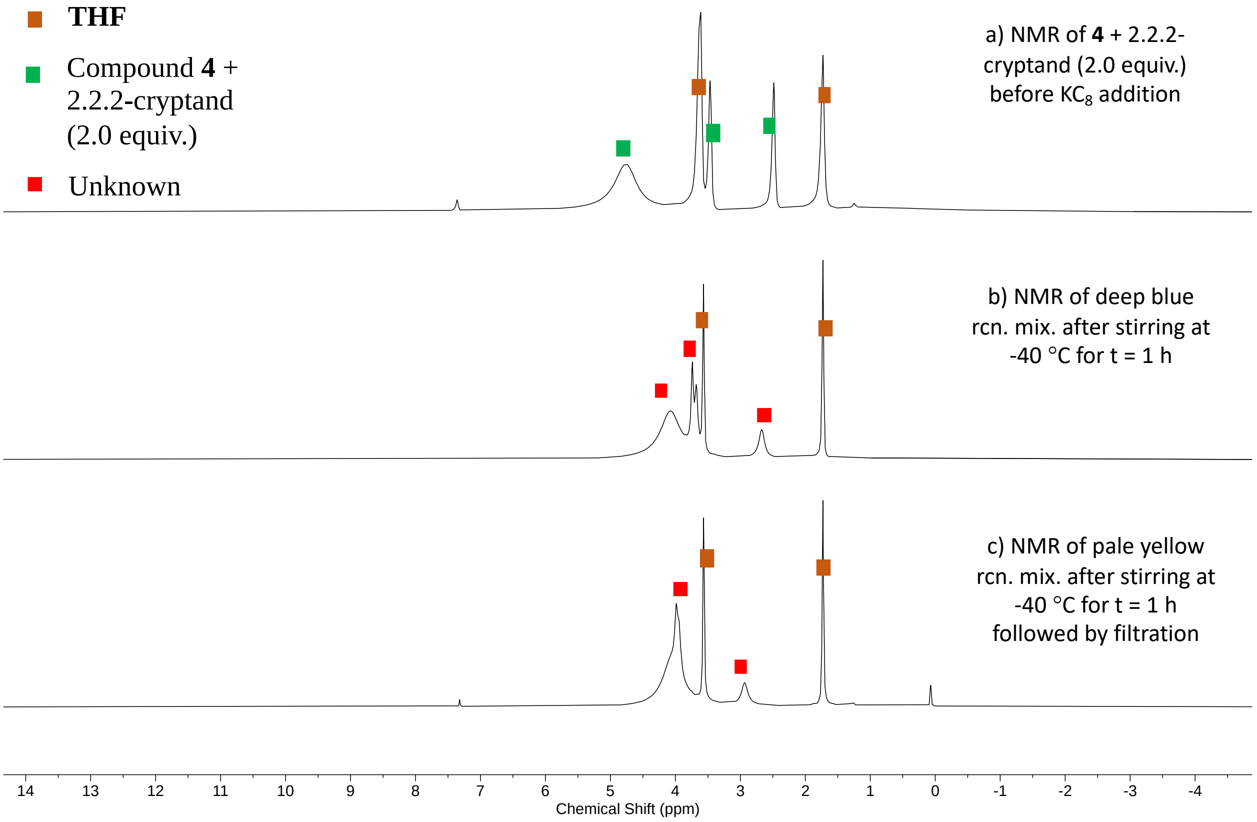

Figure S27. ^{1}H NMR spectrum (400 MHz, d_8-THF, 233 K) of a) **4** and 2.0 equiv. 2.2.2-cryptand, b) the deep blue reaction mixture obtained by reacting **4** and 2.0 equiv. 2.2.2-cryptand with 10.0 equiv. KC_8 in d_8-THF under dinitrogen at -40 °C for 1 h and c) the pale-yellow reaction mixture obtained when the original reaction mixture in b) is filtered using a glass wool pipette.

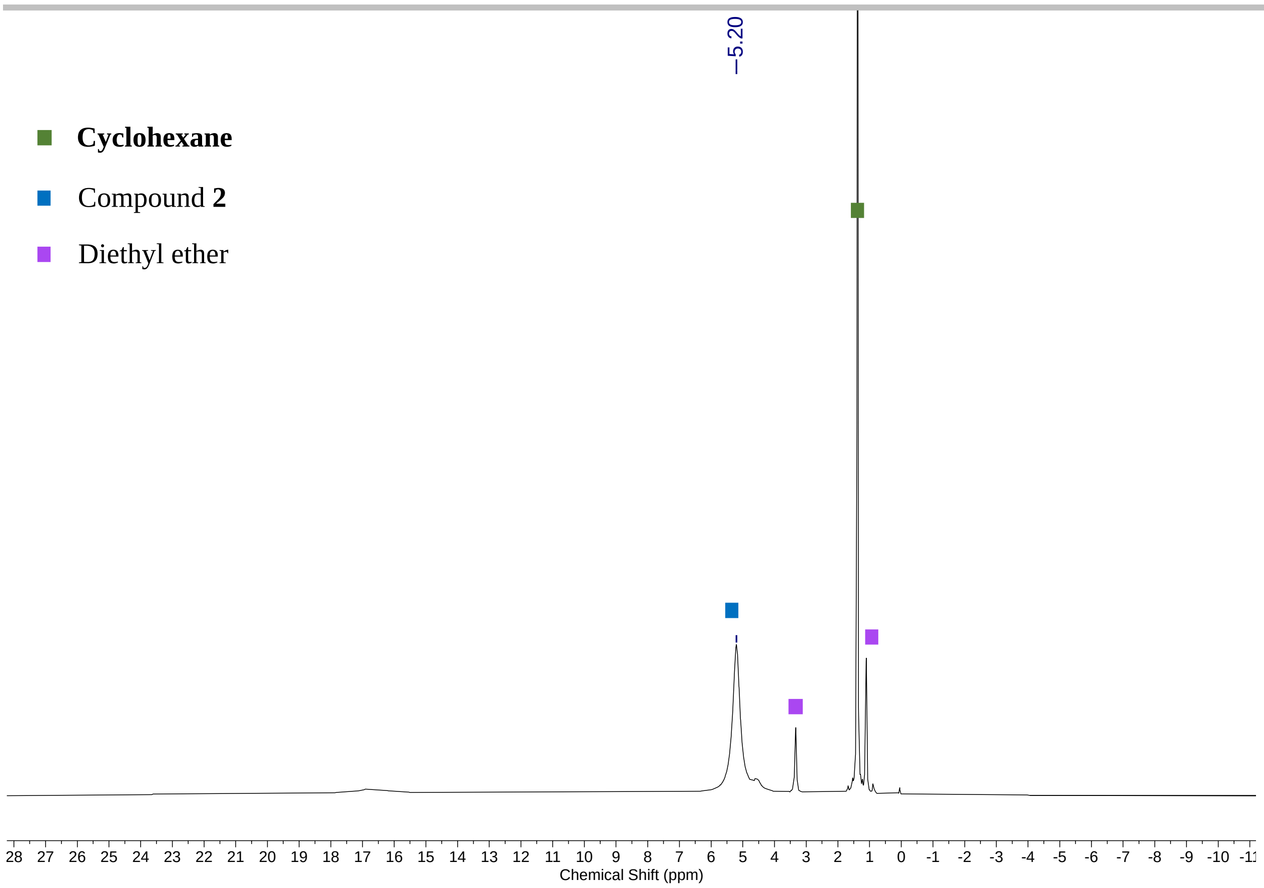

Figure S28. ^{1}H NMR spectrum (400 MHz, d_{12}-cyclohexane, 298 K) of the crude reaction mixture obtained from reacting **1** with 5.0 equiv. KC_8 at -40 °C overnight (16 h) in Et_2O to yield **2**.

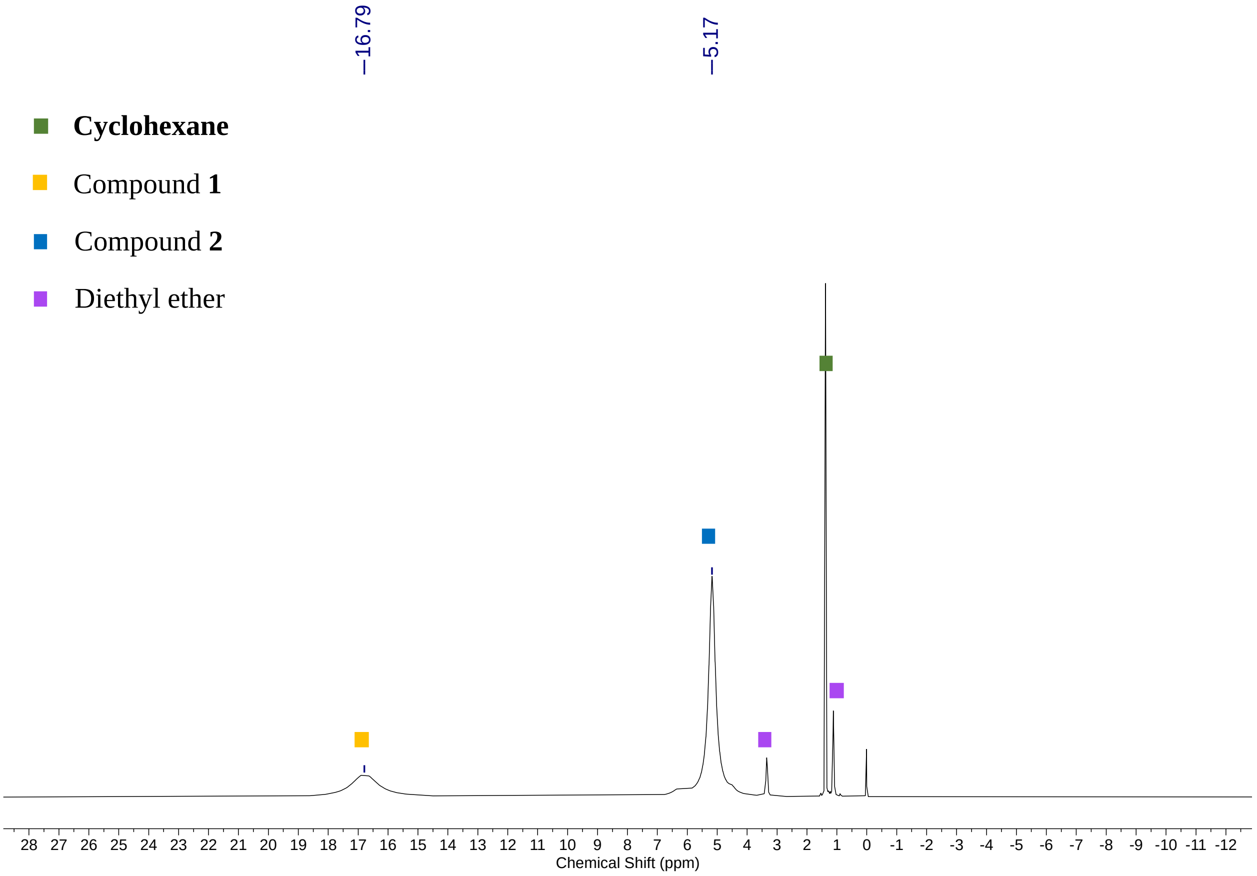

Figure S29. ^{1}H NMR spectrum (400 MHz, d_{12}-cyclohexane, 298 K) of the crude reaction mixture obtained from reacting **1** with 1.2 equiv. KC_8 at -40 °C overnight (16 h) in Et_2O.

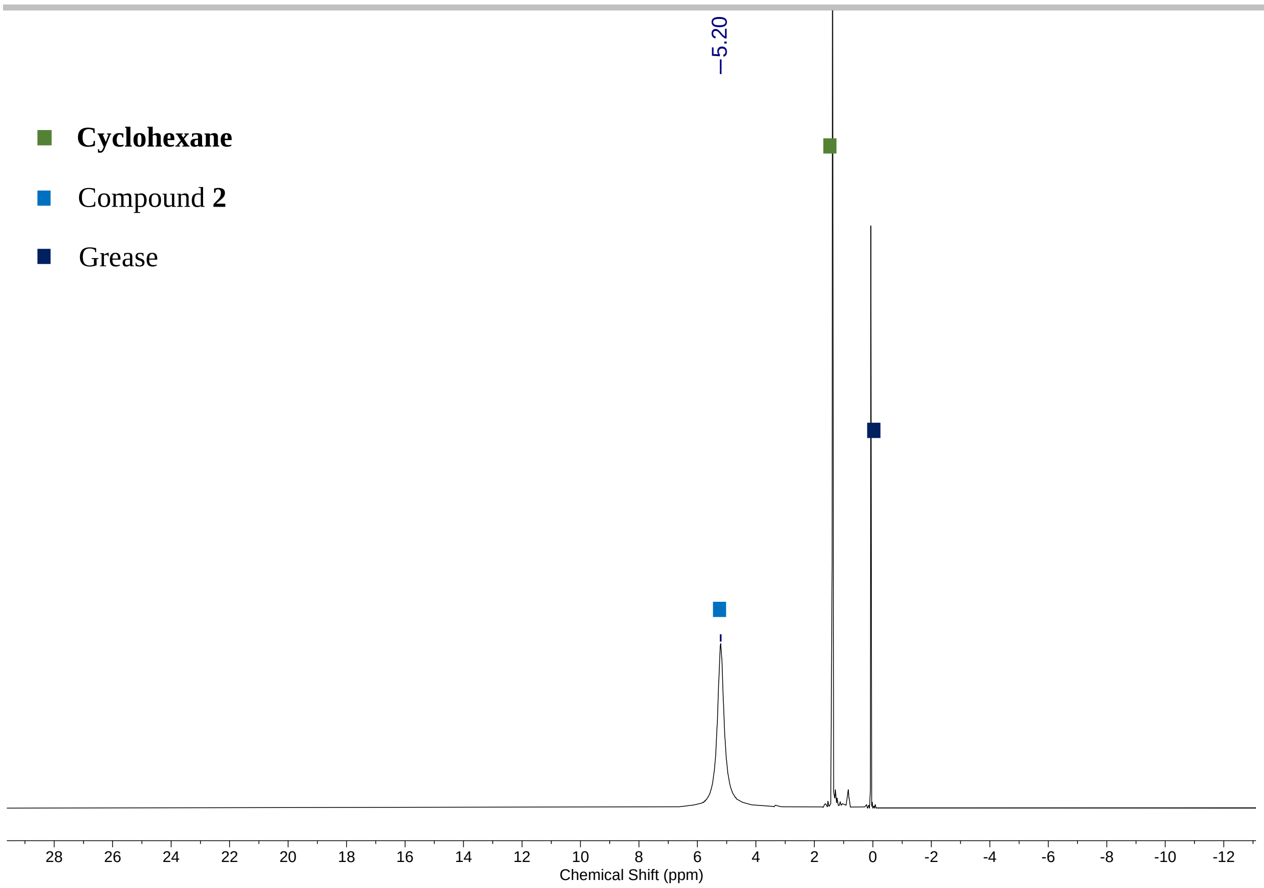

Figure S30. ^{1}H NMR spectrum (400 MHz, d_{12}-cyclohexane, 298 K) of isolated **2**.

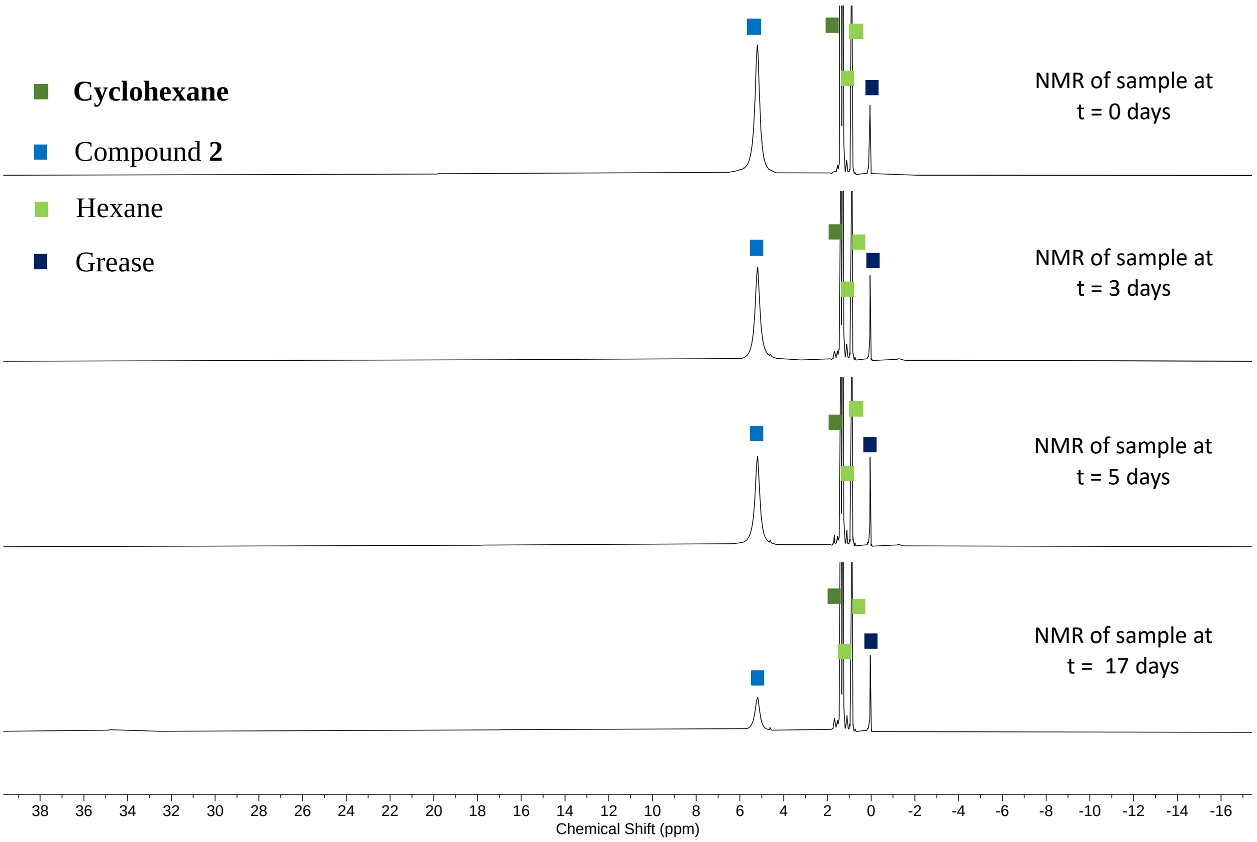

Figure S31. Evolution of the ^{1}H NMR spectrum (400 MHz, d_{12}-cyclohexane, 298 K) of a cyclohexane solution of **2** after dissolution (sample was stored at r.t. at all times).

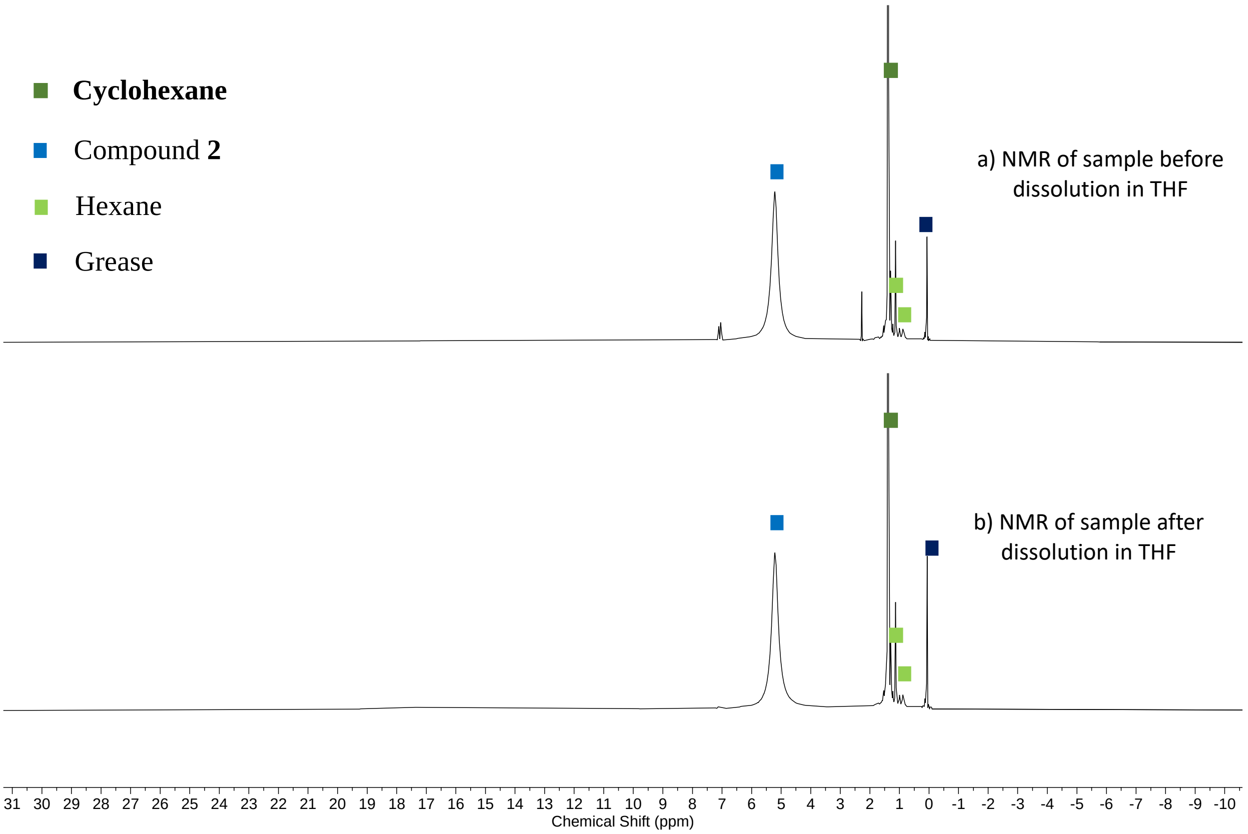

Figure S32. ^{1}H NMR spectrum (400 MHz, d_{12}-cyclohexane, 298 K) of a) isolated **2** and b) isolated **2** immediately after dissolution in THF and removal of the volatiles.

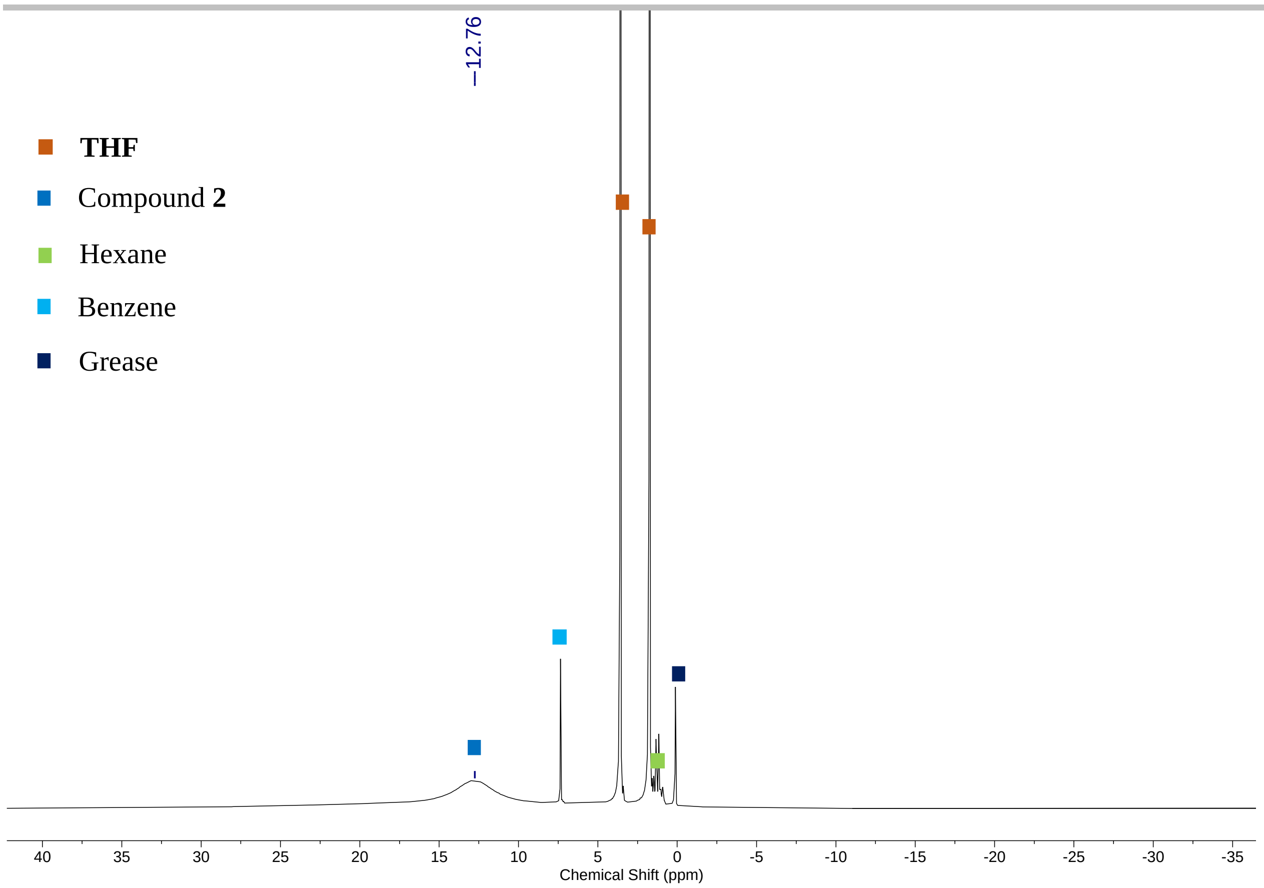

Figure S33. ^{1}H NMR spectrum (400 MHz, d_8-THF, 233 K) of isolated **2**.

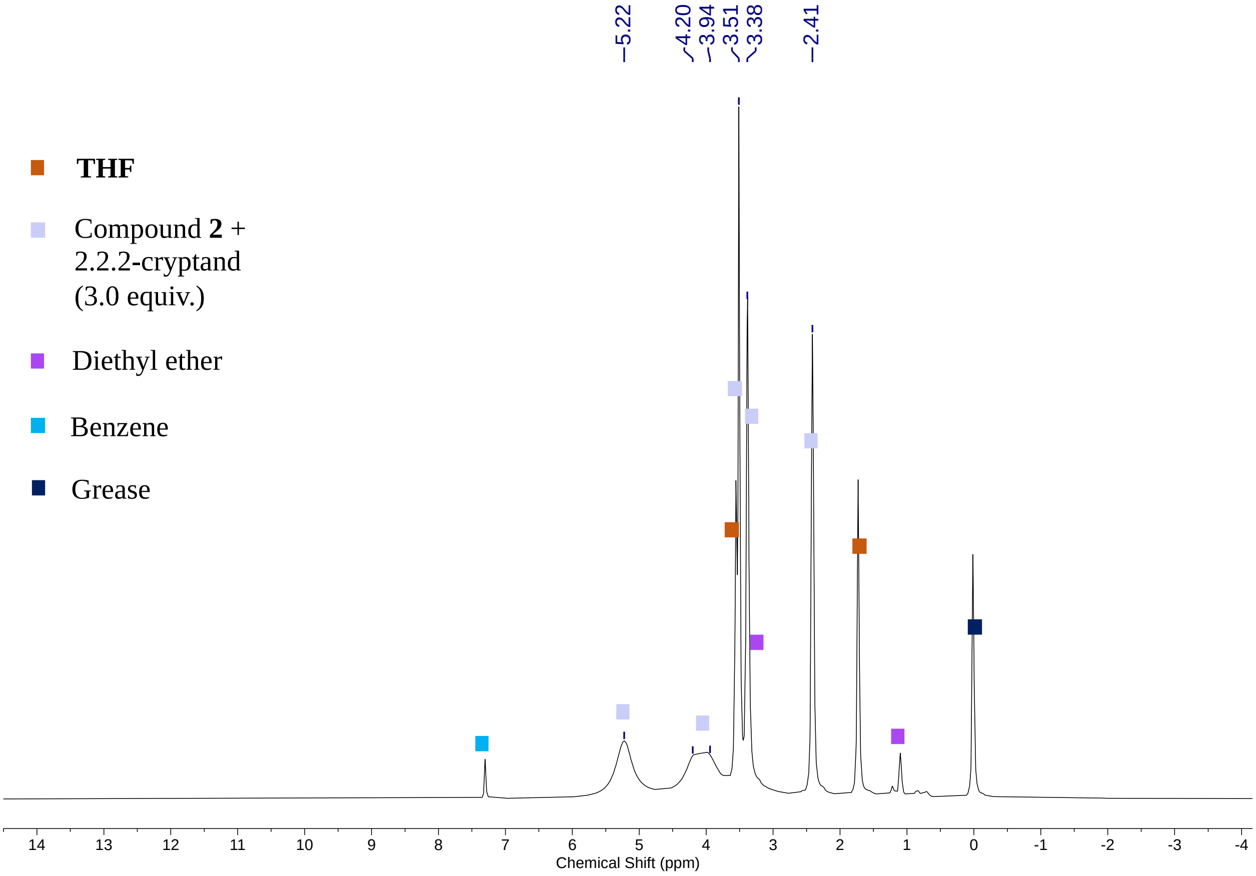

Figure S34. ^{1}H NMR spectrum (400 MHz, d_8-THF, 233 K) of the resulting reaction mixture obtained upon the addition of a solution of 2.2.2-cryptand (3.0 equiv.) in d_8-THF to a solution of complex **2** in d_8-THF (*NOTE*: the addition of the reagents was performed at -40 °C)

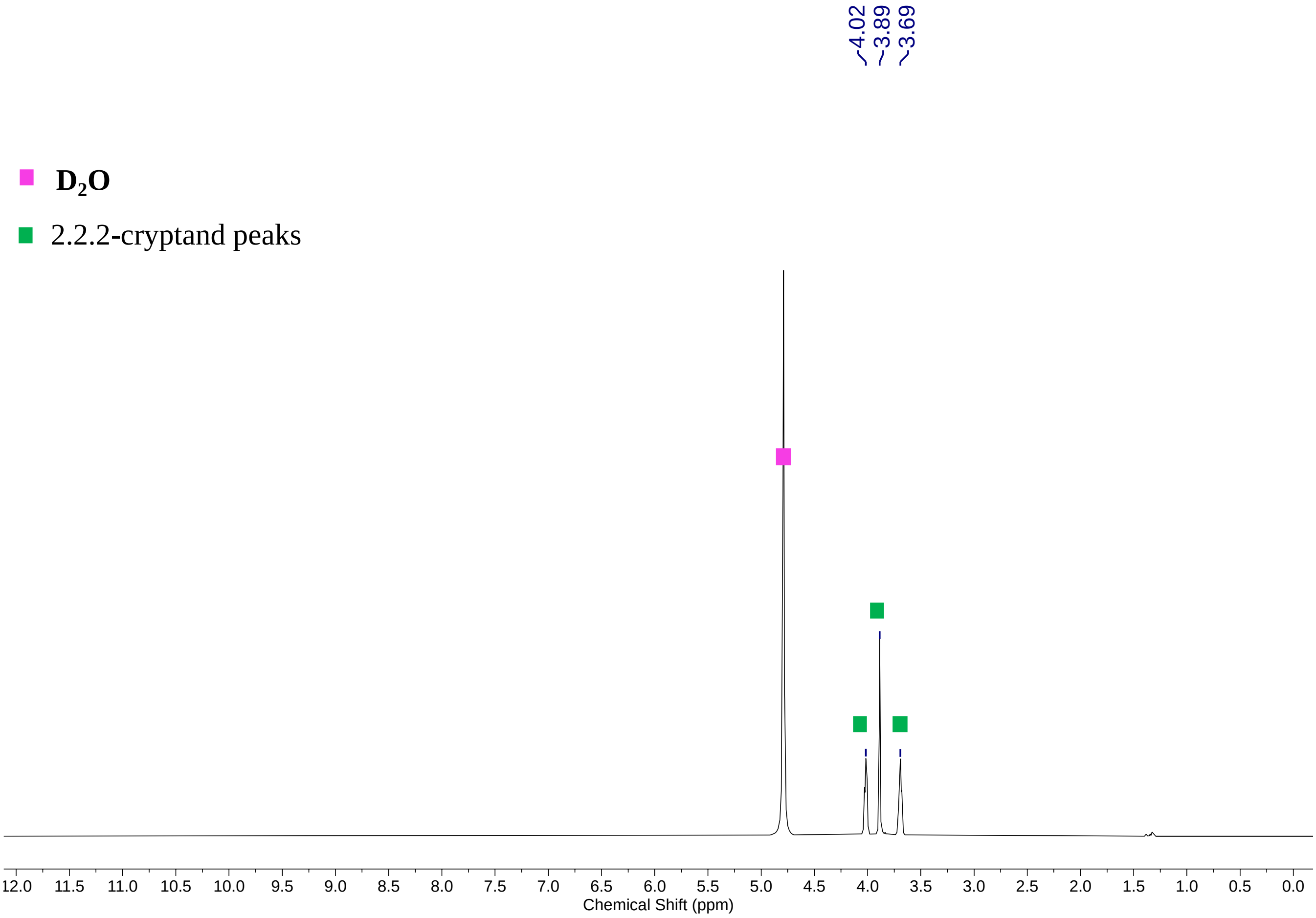

Figure S35. ^{1}H NMR spectrum (400 MHz, D_2O, 298 K) of the colorless solution obtained after extraction of the soluble material resulting from the acid quenching (1 M HCl in Et_2O) of the reaction between complex **2** and 2.2.2-cryptand (3.0 equiv.) with 1.0 equiv. MeOTf in hexane at r.t.

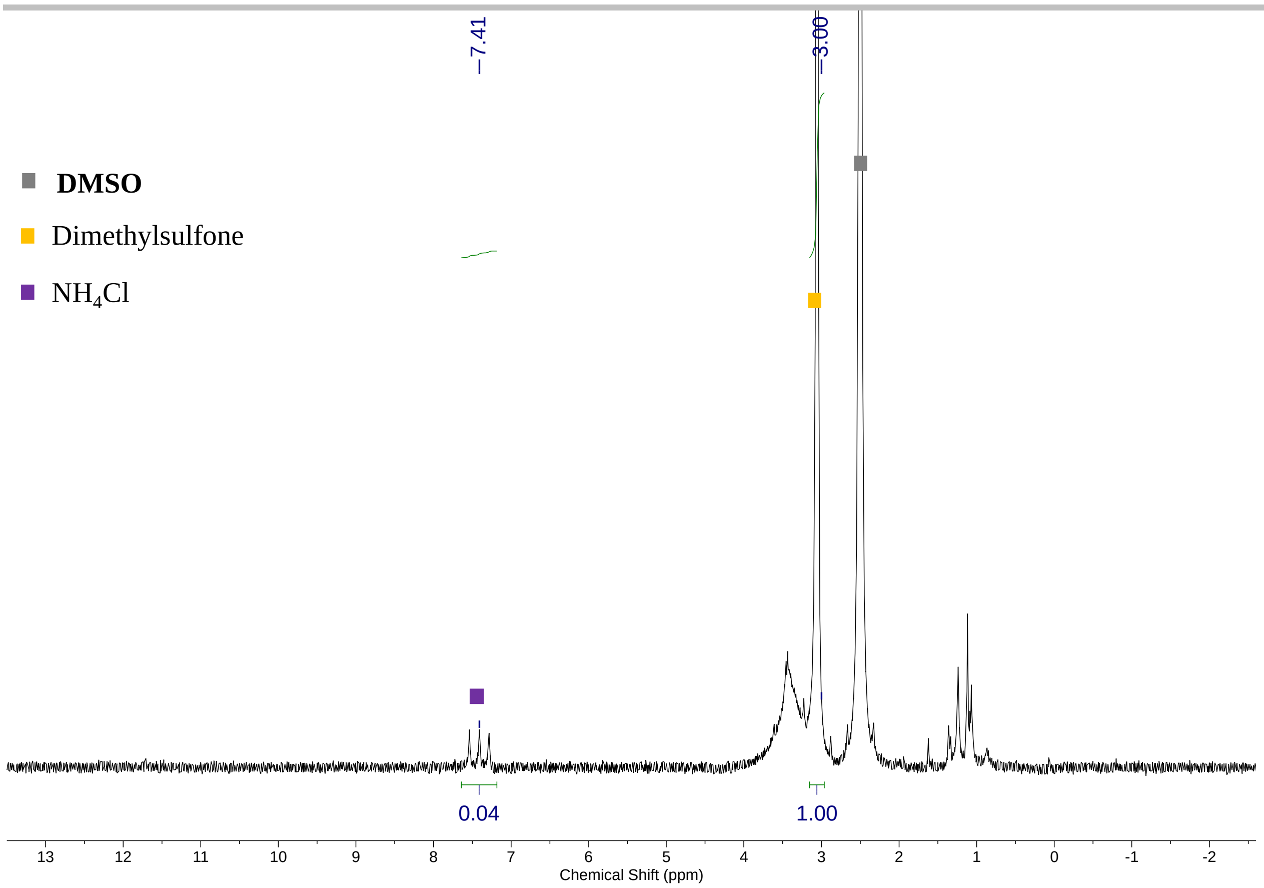

Figure S36. ^{1}H NMR spectrum (400 MHz, d_6-DMSO, 298 K) of the residue obtained after the evaporation of the reaction mixture obtained from the addition of excess 2M HCl (Et_2O) to isolated complex **2**.

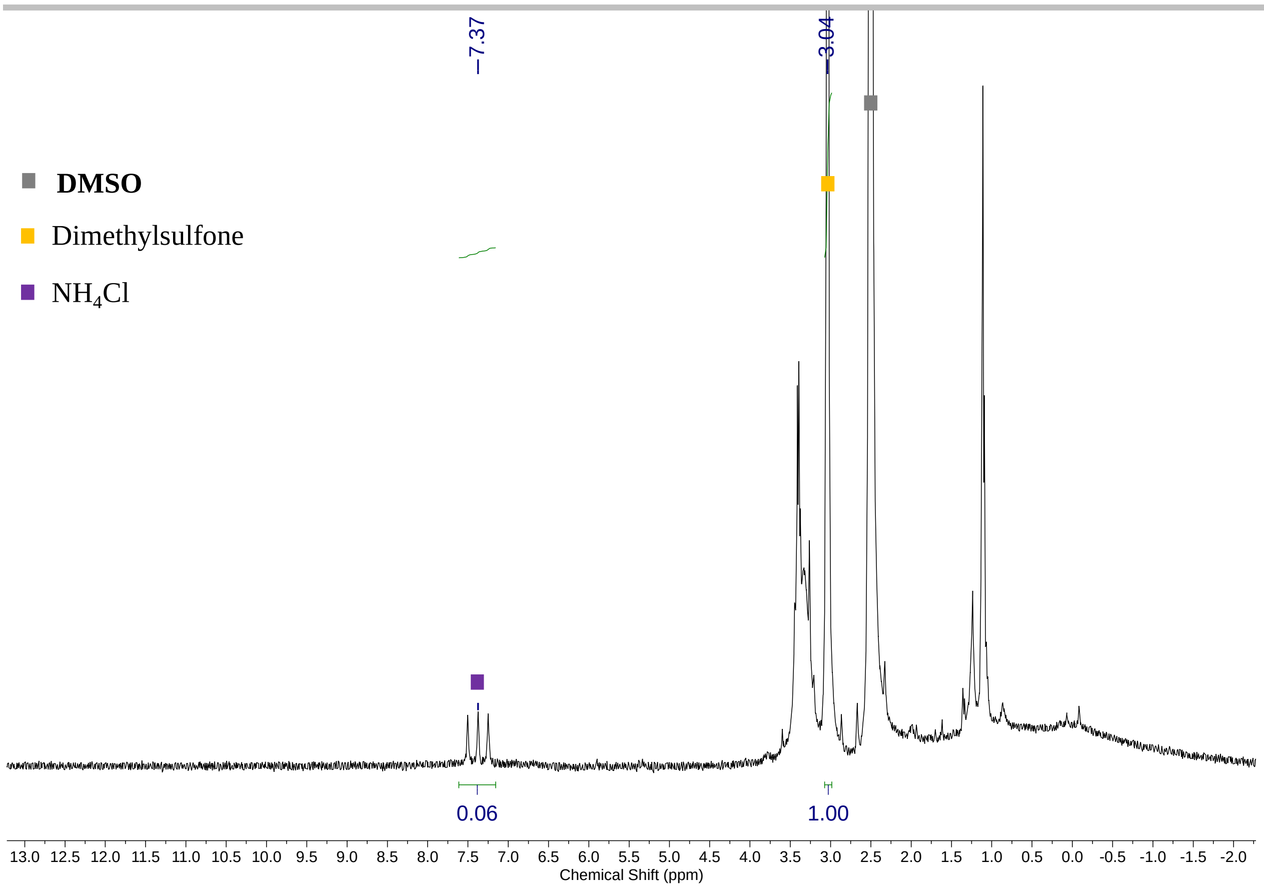

Figure S37. ^{1}H NMR spectrum (400 MHz, d_6-DMSO, 298 K) of the residue obtained after the evaporation of the reaction mixture obtained from the addition of excess 2M HCl (Et_2O) to the crude reaction mixture obtained by reacting **1** with 5.0 equiv. KC_8 at -40 °C overnight (16 h) in Et_2O.

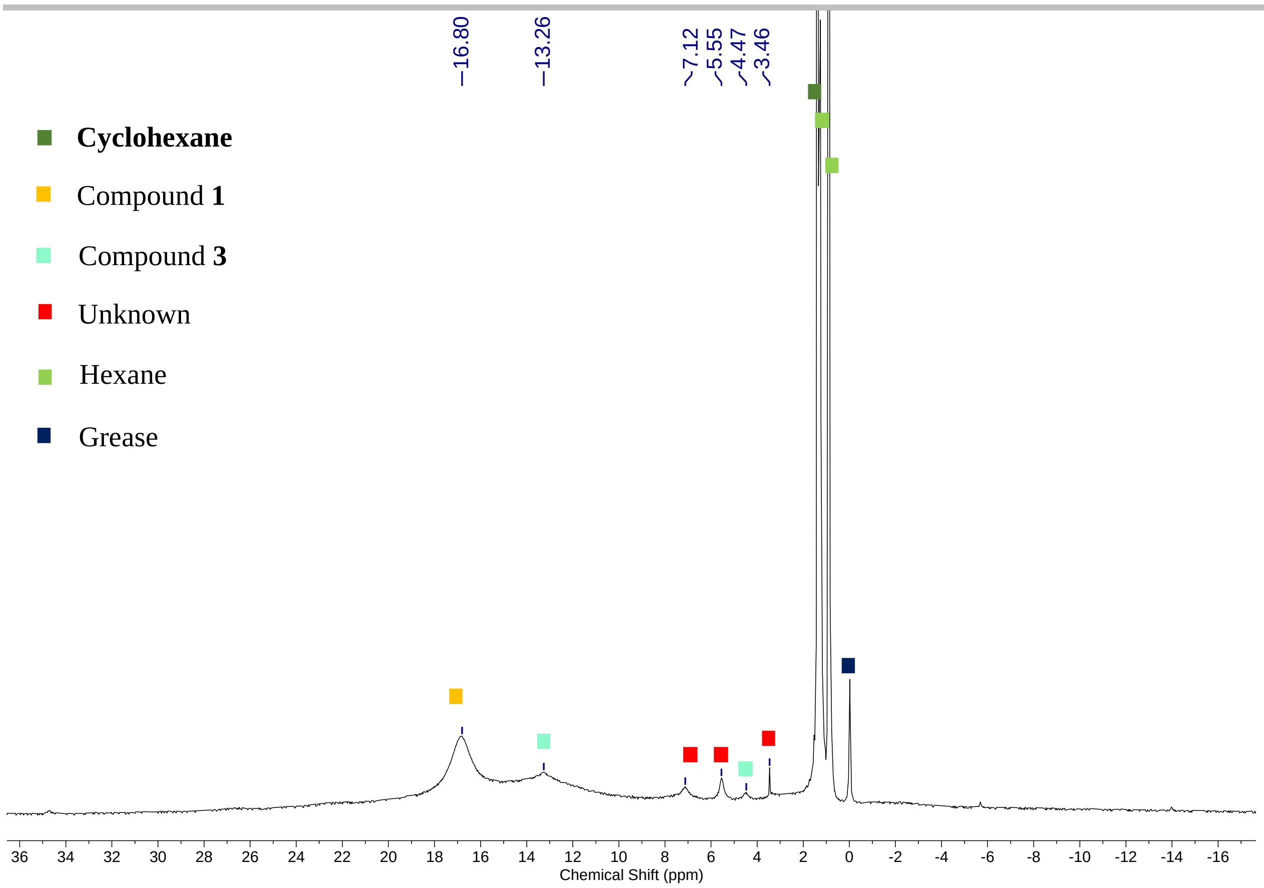

Figure S38. ^{1}H NMR spectrum (400 MHz, d_{12}-cyclohexane, 298 K) of the crude reaction mixture obtained from the reaction of **2** with 1.0 equiv. MeOTf in hexane at r.t.

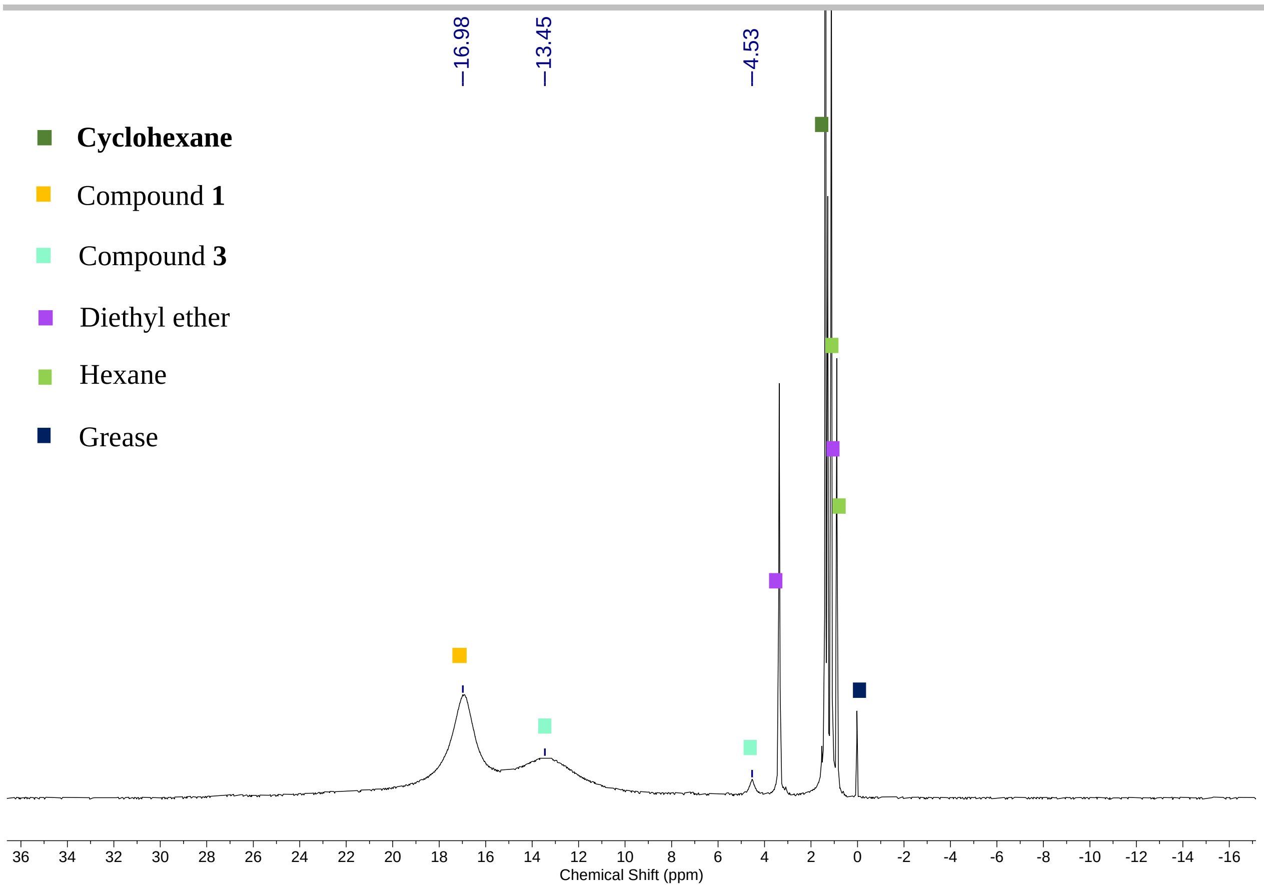

Figure S39. ^{1}H NMR spectrum (400 MHz, *d*$_{12}$-cyclohexane, 298 K) of the yellow microcrystalline solid isolated from the reaction of **2** with 1.0 equiv. MeOTf in hexane at r.t. (*NOTE*: the yellow microcrystalline solid is a mixture of **1** and **3**)

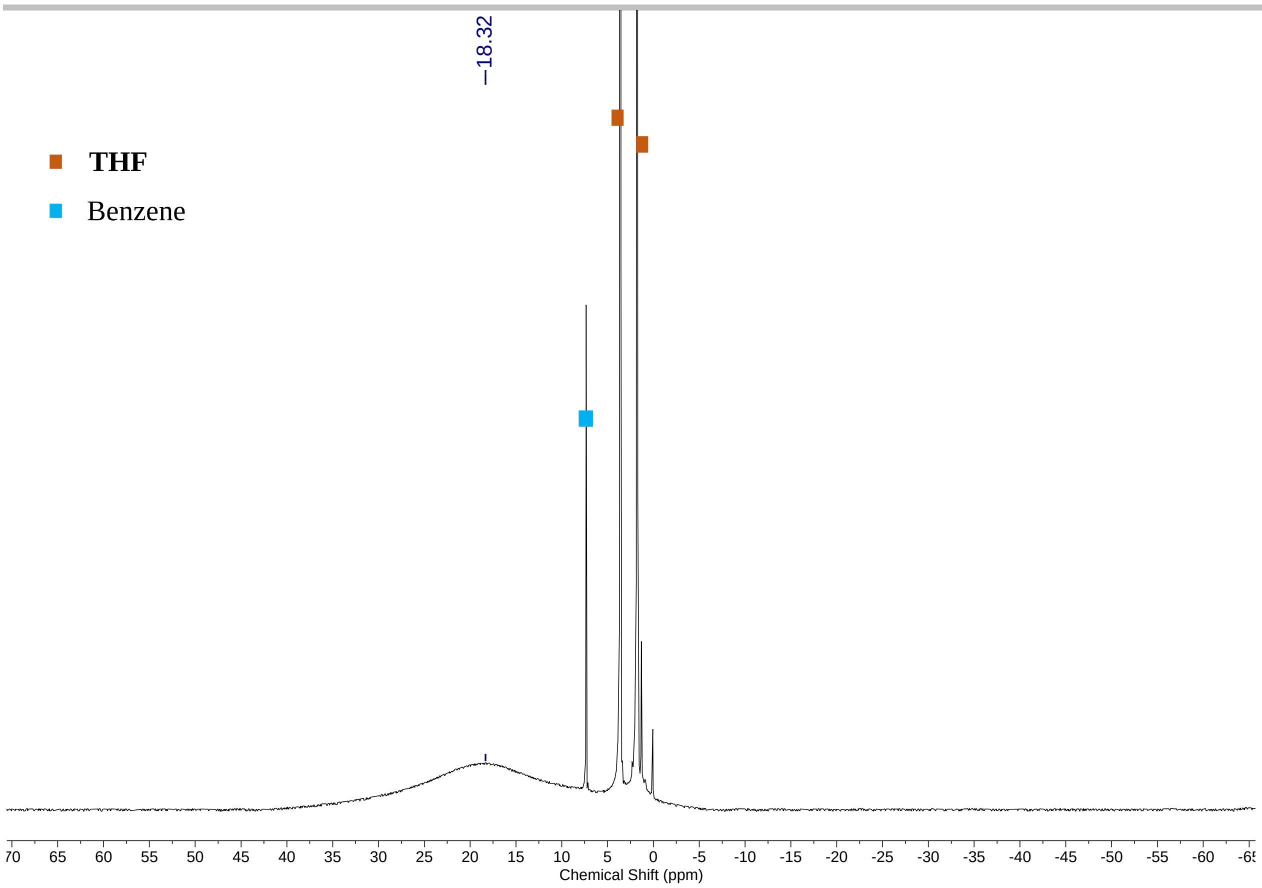

Figure S40. ^{1}H NMR spectrum (400 MHz, d_8-THF, 233 K) of the yellow microcrystalline solid isolated from the reaction of **2** with 1.0 equiv. MeOTf in hexane at r.t. (*NOTE*: the yellow microcrystalline solid is a mixture of **1** and **3**)

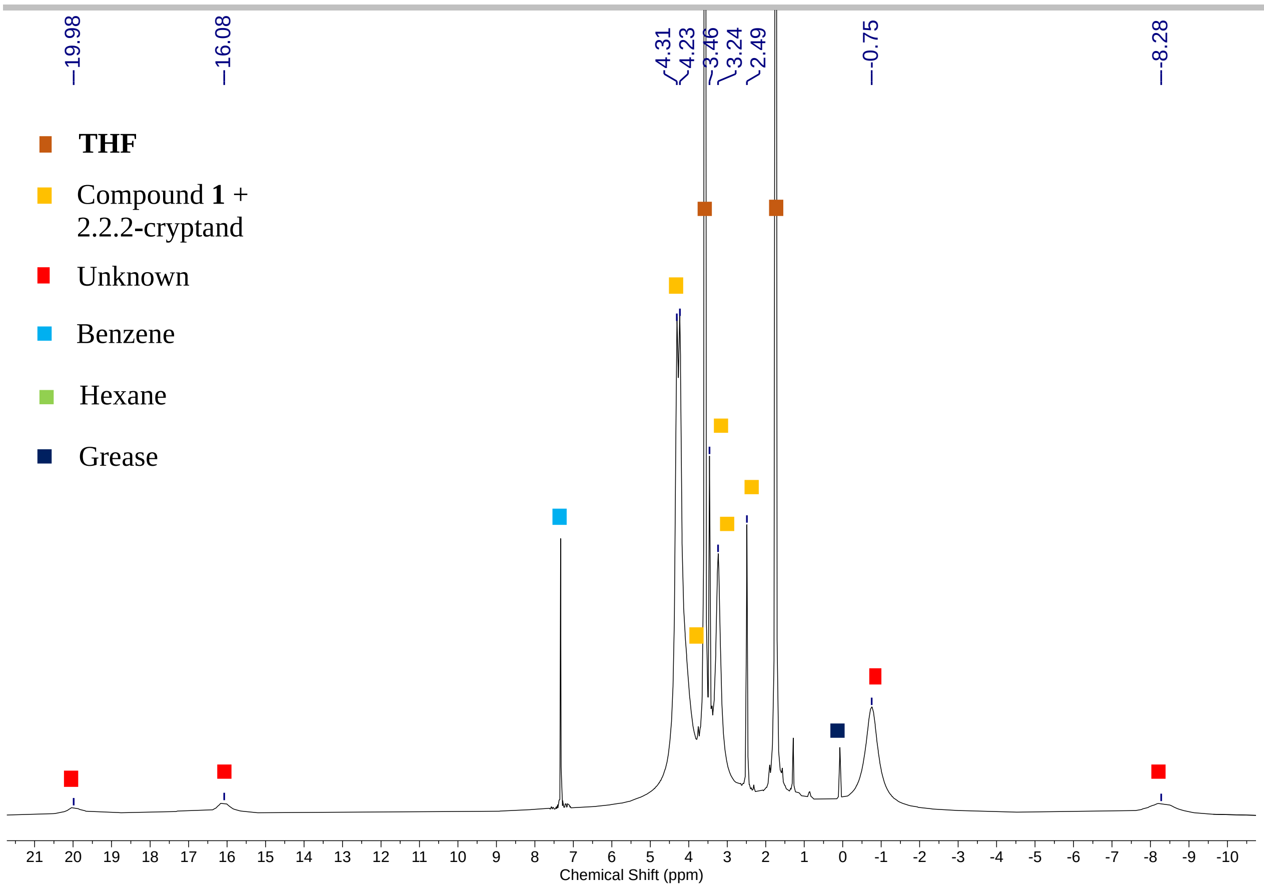

Figure S41. ^{1}H NMR spectrum (400 MHz, d_8-THF, 233 K) of the resulting reaction mixture upon the addition of a solution of 2.2.2-cryptand (2.0 equiv.) in d_8-THF to a solution of the yellow microcrystalline solid isolated from the reaction of **2** with 1.0 equiv. MeOTf, in d_8-THF (*NOTE*: the yellow microcrystalline solid is a mixture of **1** and **3** and the addition of the reagents was performed at -40 °C)

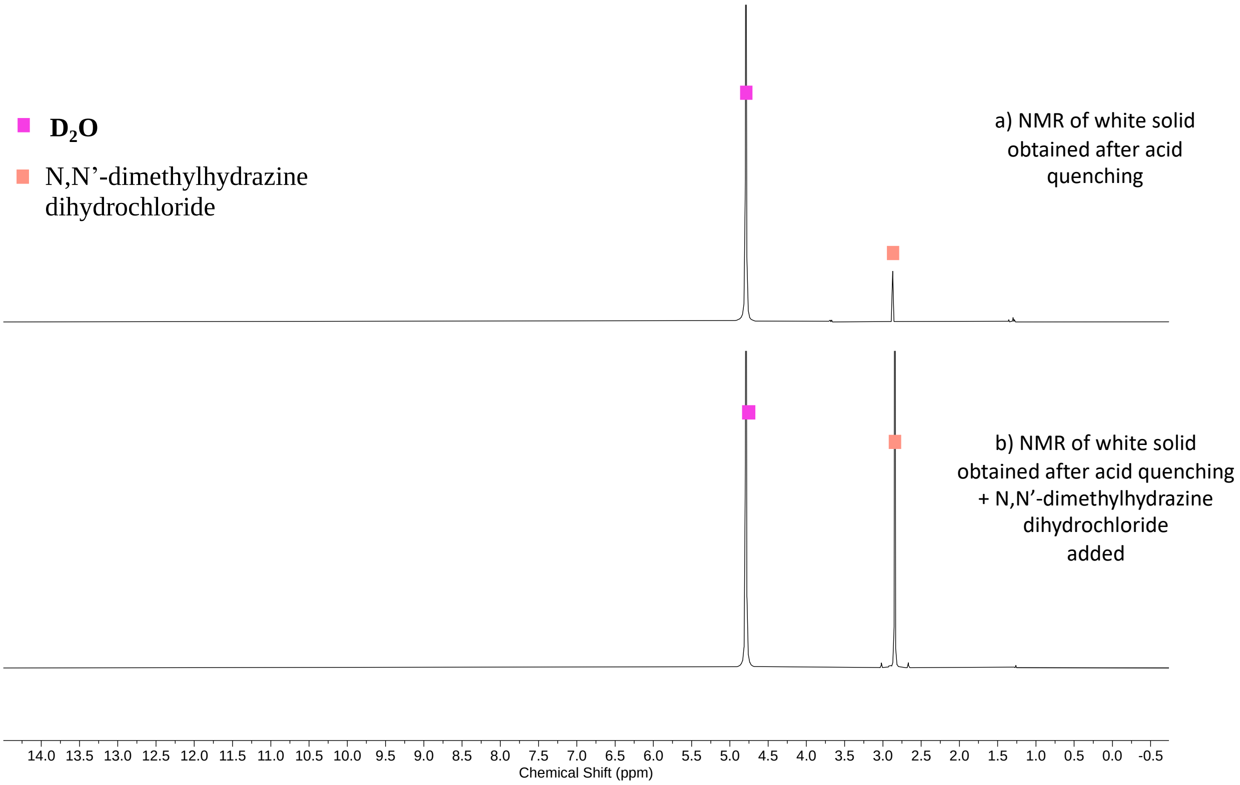

Figure S42. ^{1}H NMR spectrum (400 MHz, D_2O, 298 K) of a) the white solid obtained after the addition of excess 2M HCl (Et_2O) to the yellow microcrystalline solid (which is a mixture of **1** and **3)** isolated from the reaction of **2** with 1.0 equiv. of MeOTf and b) the resulting solution after the addition of commercially available N,N’-dimethylhydrazine dihydrochloride (5.0 mg) to the original sample in a).

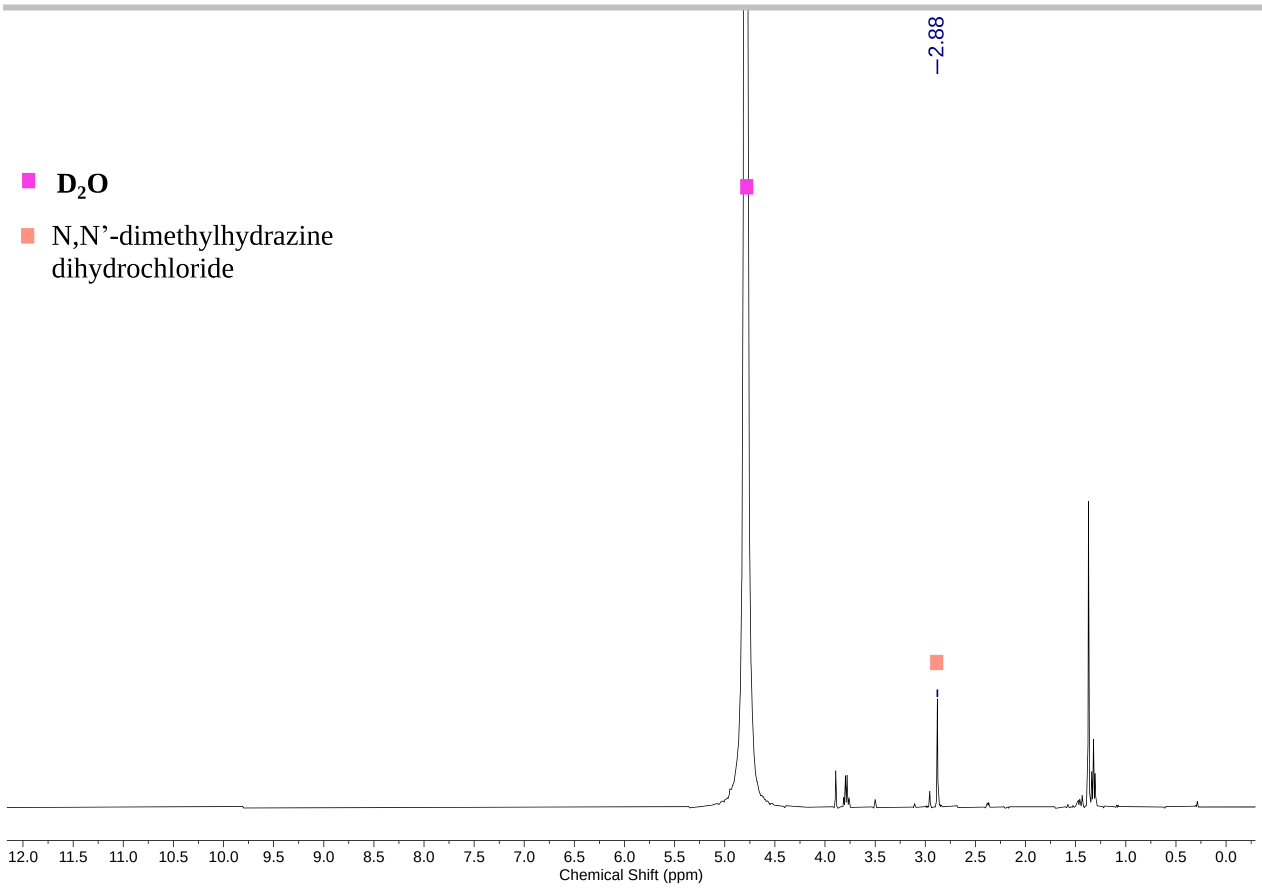

Figure S43. ^{1}H NMR spectrum (400 MHz, D_2O, 298 K) of the colorless solution obtained after extraction of the soluble material resulting from the acid quenching (1 M HCl in Et_2O) of the reaction between isolated complex **2** with 1.0 equiv. MeOTf in hexane at r.t.

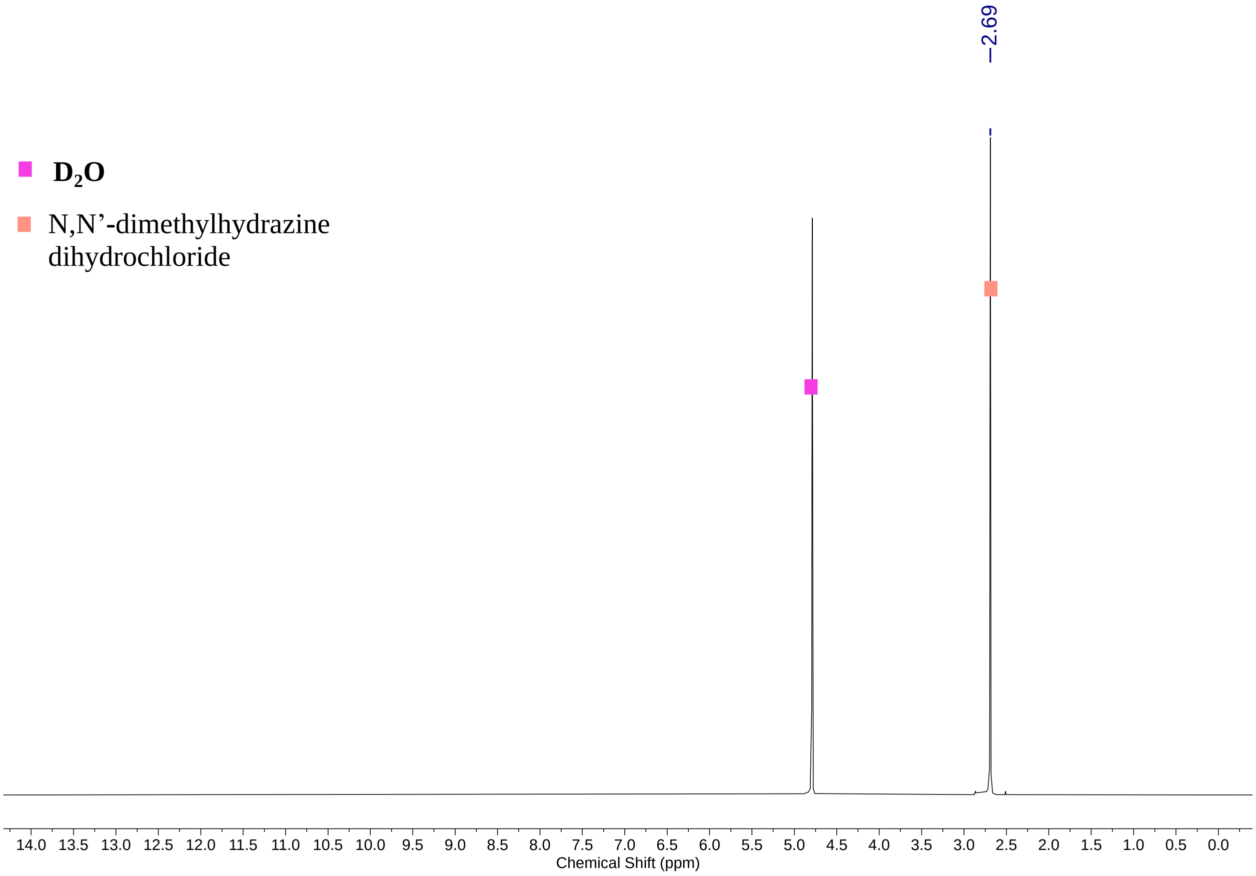

Figure S44. ^{1}H NMR spectrum (400 MHz, D_2O, 298 K) of commercially available N,N'-dimethylhydrazine dihydrochloride (concentration = 0.2 M).

C. X-Ray Crystal Structure Determination Details

Suitable crystals were selected and mounted on various Rigaku diffractometers (XtaLAB Synergy R, DW system, HyPix-Arc 150 detector or SuperNova, Dual, Cu at home/near, AtlasS type detectors). The crystals were kept at a steady temperature during data collection. Data were measured using ω scans with Cu K_{α} radiation. The diffraction patterns were indexed and the total number of runs and images were based on the strategy calculation from the program CrysAlisPro 1.171.43.124a.[11] The unit cells were refined using CrysAlisPro 1.171.43.124a.[11] Data reduction, scaling and absorption corrections were performed using CrysAlisPro 1.171.43.124a.[11]

The structures were solved with the **ShelXT** solution program using dual methods and by using **Olex2** 1.5 as the graphical interface.[12–14] The models were refined with **ShelXL** 2019/3 using full matrix least squares minimisation on $\boldsymbol{F^2}$.[12,13] All non-hydrogen atoms were refined anisotropically. Hydrogen atom positions were calculated geometrically and refined using the riding model.

Several issues were encountered during the stages of the refinement of the crystal structures presented in this paper. All structures except compound **6** showed disordered moieties and numerous restraints (SADI, SIMU, DFIX, RIGU, ISOR) were employed to get acceptable geometric and anisotropic parameters. The data of compound **4** were treated for twinning (BASF parameter = 0.06667). The solvent displayed disorder problems in 4 (out of 6) structures: **1**, **2**, **3**, **5**. It was masked by the Olex2 algorithm based on BYPASS/SQUEEZE (by P. van der Sluis and A.L. Spek).

SUPPORTING INFORMATION

Table S1. Crystal data and structural refinement parameters for complexes [{KTm(OSi(O^{t}Bu)$_3$)$_3$}$_2$(μ-η^2:η^2-N_2)].(hexane)$_2$ (**1**.(hexane)$_2$), [K_3{Tm(OSi(O^{t}Bu)$_3$)$_3$}$_2$(μ-η^2:η^2-N_2)].(hexane)$_{1.5}$ (**2**.(hexane)$_{1.5}$) and [K_2{Tm(OSi(O^{t}Bu)$_3$)$_3$}$_2$(μ-(CH_3)NN(CH_3))])].(toluene)$_{0.8}$ (**3**.(toluene)$_{0.8}$)

Compound	**1**.(hexane)$_2$	**2**.(hexane$_{1.5}$)	**3**.(toluene)$_{0.8}$
Formula	$C_{84}H_{190}K_2N_2O_{24}Si_6Tm_2$	$C_{81}H_{183}K_3N_2O_{24}Si_6Tm_2$	$C_{79.6}H_{174.4}K_2N_2O_{24}Si_6Tm_2$
Crystal Size (mm)	0.70×0.48×0.37	0.32×0.09×0.08	0.19×0.11×0.07
Crystal System	triclinic	monoclinic	triclinic
Space Group	*P*-1	*C*2/*c*	*P-1*
Volume (Å^3)	3011.47(11)	23869.3(8)	2792.0(3)
a (Å)	13.8506(3)	57.0067(9)	13.8480(7)
b (Å)	14.7414(3)	14.4168(3)	13.8494(10)
c (Å)	17.3225(3)	29.2129(6)	18.1759(8)
α (°)	112.2909(17)	90	94.210(4)
β (°)	107.8880(18)	96.1788(17)	107.598(4)
γ (°)	96.1969(15)	90	119.331(6)
Z	1	8	1
Formula Weight	2196.97	2192.98	2128.40
Density (g cm^{-3})	1.211	1.220	1.266
μ (mm^{-1})	4.335	4.681	4.661
F (000)	1160	9232	1118
Temperature (K)	200.00(10)	199.99(10)	140.00(10)
Total Reflections	35279	63037	19772
Unique Reflections	12528	22863	10742
R_{int}	0.0217	0.0341	0.0602
R Indices [I >2σ(I)]	R_1 = 0.0344<br>wR_2 = 0.0934	R_1 = 0.0340<br>wR_2 = 0.0855	R_1 = 0.0597<br>wR_2 = 0.1407
Largest Diff. Peak and Hole (e A^{-3})	1.964 and -1.477	0.461 and -0.735	1.868 and -1.722
GooF	1.040	1.005	1.016
CCDC	2357977	2357978	2357979

Table S2. Crystal data and structural refinement parameters for complexes [Tm(OSi(O^{t}Bu)$_3$)$_2$(μ-OSi(O^{t}Bu)$_3$)]$_2$ (**4**), [KTm(OSi(O^{t}Bu)$_3$)$_4$] (**5**) and [K(crypt)]$_2$[{Tm(OSi(O^{t}Bu)$_3$)$_3$}$_2$(μ-η^2:η^2-N$_2$)] (**1-crypt**).

Compound	**4**	**5**	**1-crypt**
Formula	$C_{72}H_{162}O_{24}Si_6Tm_2$	$C_{60}H_{136}KO_{20}Si_5Tm$	$C_{116}H_{254}K_2N_6O_{38}Si_6Tm_2$
Crystal Size (mm)	0.06×0.04×0.02	0.24×0.15×0.12	0.30×0.16×0.02
Crystal System	monoclinic	trigonal	triclinic
Space Group	*C*2/*c*	*R*3*c*	*P*-1
Volume (Å^3)	9801.1(3)	12704.8(3)	3872.62(8)
a (Å)	25.5278(5)	18.8611(2)	15.71983(19)
b (Å)	14.2088(2)	18.8611(2)	16.5222(2)
c (Å)	27.3258(4)	41.2384(4)	16.65284(16)
α (°)	90	90	114.7319(11)
β (°)	98.5618(16)	90	95.9275(10)
γ (°)	90	120	94.4242(10)
Z	4	6	1
Formula Weight	1918.41	1526.16	2925.84
Density (g cm^{-3})	1.300	1.197	1.255
μ (mm^{-1})	4.501	3.547	3.574
F (000)	4032	4884	1556
Temperature (K)	140.00(10)	200.00(10)	139.99(10)
Total Reflections	12523	70689	82085
Unique Reflections	12523	5952	15756
R_{int}	n/a	0.0391	0.0426
R Indices [I >2σ(I)]	R_1 = 0.0986<br>wR_2 = 0.2660	R_1 = 0.0362<br>wR_2 = 0.1046	R_1 = 0.0422<br>wR_2 = 0.1071
Largest Diff. Peak and Hole (e A^{-3})	1.181 and -2.157	0.521 and -0.519	1.415 and -2.224
GooF	1.063	1.059	1.041
CCDC	2355585	2357980	2355714

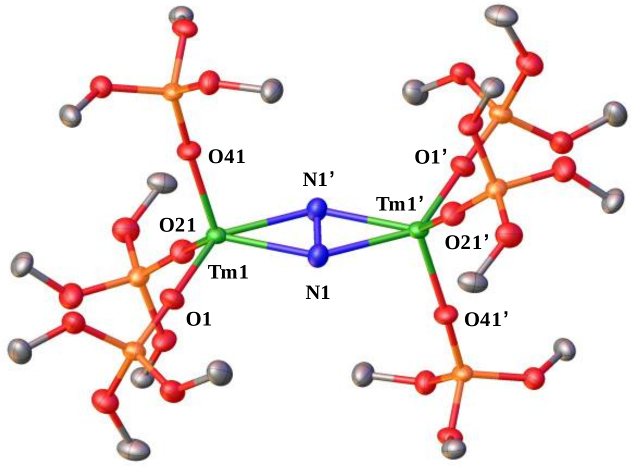

Figure S45. Molecular structure of $[K(crypt)]_2[\{Tm(OSi(O^tBu)_3)_3\}_2(\mu\text{-}\eta^2\text{:}\eta^2\text{-}N_2)]$ (**1-crypt**) with thermal ellipsoids drawn at the 50% probability level. Methyl groups, hydrogen atoms and 2.2.2-cryptand moieties (x2) have been omitted for clarity. Selected bond lengths (Å): Tm1-$O_{siloxide}$ = 2.122(2), 2.127(2), 2.135(2); N1-N1' = 1.271(6).

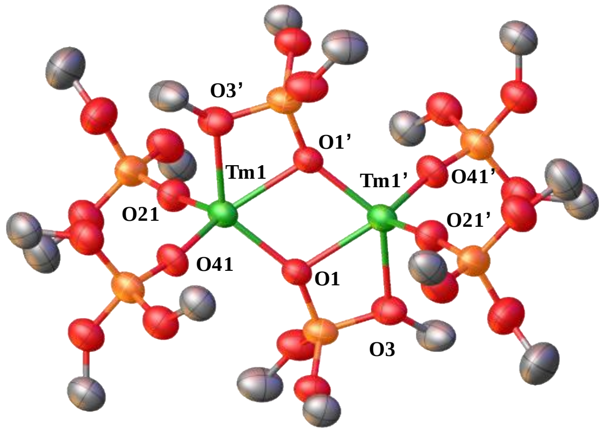

Figure S46. Molecular structure of $[Tm(OSi(O^tBu)_3)_2(\mu\text{-}OSi(O^tBu)_3)]_2$ (**4**) with thermal ellipsoids drawn at the 50% probability level. Methyl groups and hydrogen atoms have been omitted for clarity. Selected bond lengths (Å): Tm1-$O_{siloxideterminal}$ = 2.076(7), 2.088(6); Tm1-$O_{siloxidebridging}$ = 2.227(7).

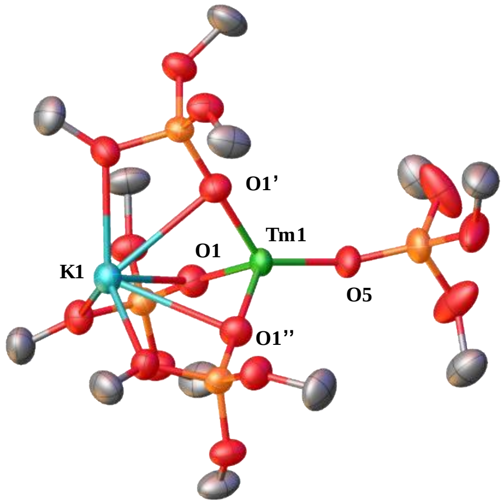

Figure S47. Molecular structure of [KTm(OSi(O^{t}Bu)$_3$)$_4$] (**5**) with thermal ellipsoids drawn at the 50% probability level. Methyl groups and hydrogen atoms have been omitted for clarity. Selected bond lengths (Å): Tm1-O$_{siloxide}$ = 2.079(4)-2.086(11).

Table S3. Selected bond lengths (Å) of complexes **1**, **1-crypt**, **2** and **3**.

Complex	**1**	**1-crypt**	**2**	**3**
N-N	1.19(4)	1.271(6)	1.22(6)	1.38(5)
Tm-O$_{siloxideaverage}$	2.107(5)	2.128(5)	2.141(6) (Tm1) ; 2.136(8) (Tm2)	2.122(10)

The Tm1-O$_{siloxide}$ (2.136(2) Å, 2.137(2) Å , 2.148(2) Å) and Tm2-O$_{siloxide}$ (2.125(2) Å, 2.140(2) Å , 2.144(2) Å) bond distances in **2** are indistinguishable from one another, further corroborating the assignment of dinuclear Tm(III)/Tm(III) complex bridged by an N_2^{3-} moiety.

D. EPR Spectroscopic Data

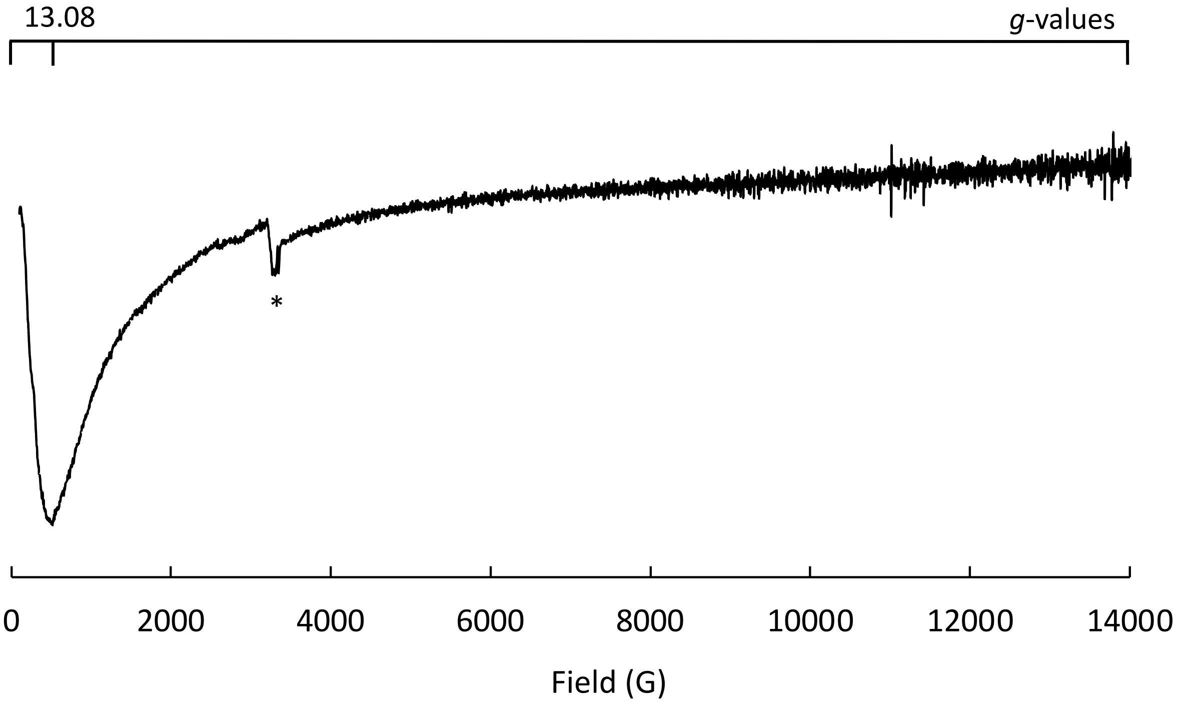

Figure S48. Frozen solution state X-band (9.40 GHz) EPR spectrum of **1** (10 mM in pentane) at 6 K. *Represents an organic radical impurity present in the sample.

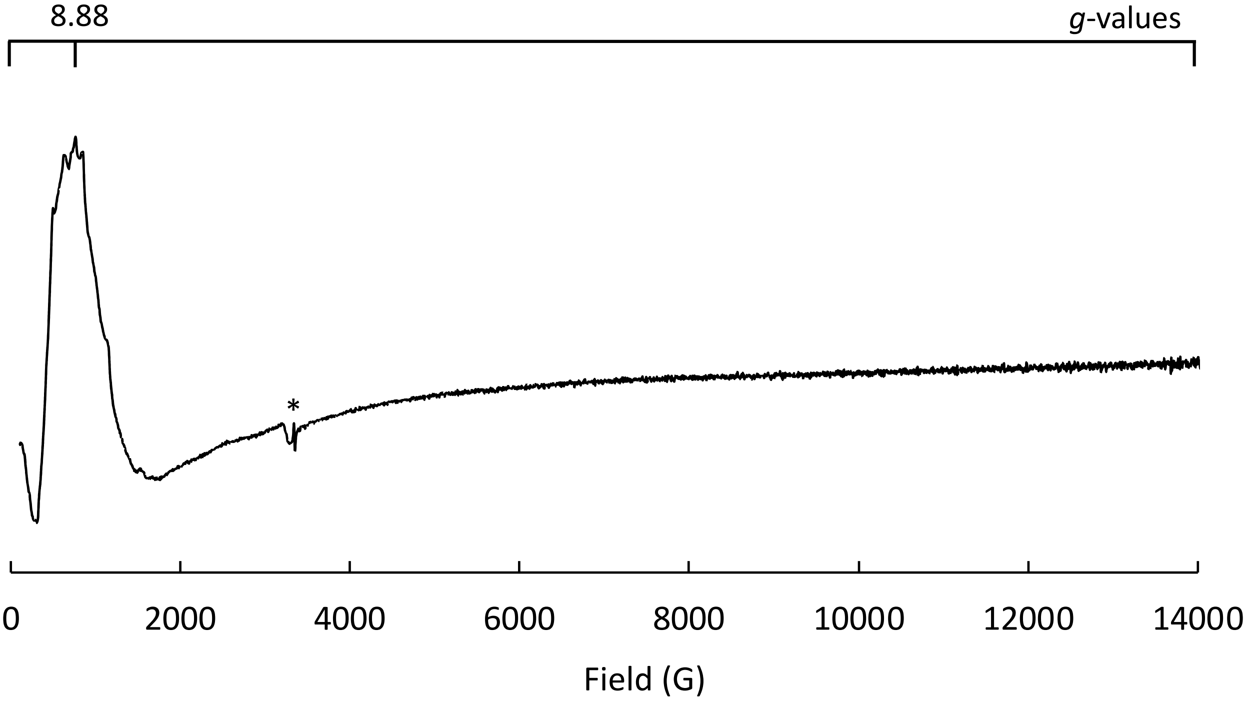

Figure S49. Frozen solution state X-band (9.40 GHz) EPR spectrum of **2** (10 mM in pentane) at 6 K. The observed super-hyperfine structure at g = 8.88 further supports the presence of a nitrogen based radical interacting with the metal ion in complex **2**.*Represents an organic radical impurity present in the sample.

E. SQUID Magnetometry Data

Direct Current (d.c.) magnetic susceptibility data were collected in the temperature range 2–300 K for complexes **1** and **2** (*vide infra*) at 1 T.

Complex **1** has a χT value of 8.4 emu K mol^{-1} per ion at 300 K (16.8 emu K mol^{-1} per complex), in good agreement with the presence of two non-interacting free $4f^{12}$ ions.[15] This value is similar to values reported for mononuclear Tm(III) complexes.[16] The value of χT decreases rapidly with decreasing temperature, reaching 1.6 emu K mol^{-1} per ion at 2 K (3.2 emu K mol^{-1} per complex).

Complex **2** shows a significantly different behavior with temperature consistent with a delocalized bonding description.

The χT value for the complex **2** of 10.7 emu K mol^{-1} at 300 K is significantly lower than the value of 15.5 emu K mol^{-1} per complex expected for two magnetically isolated Tm(III) ions and a single radical S= ½ N_2^{3-} unit in agreement with the delocalization of the electron over the N_2 and the two metal centers observed by computational studies, but could also suggest the presence of antiferromagnetic coupling.

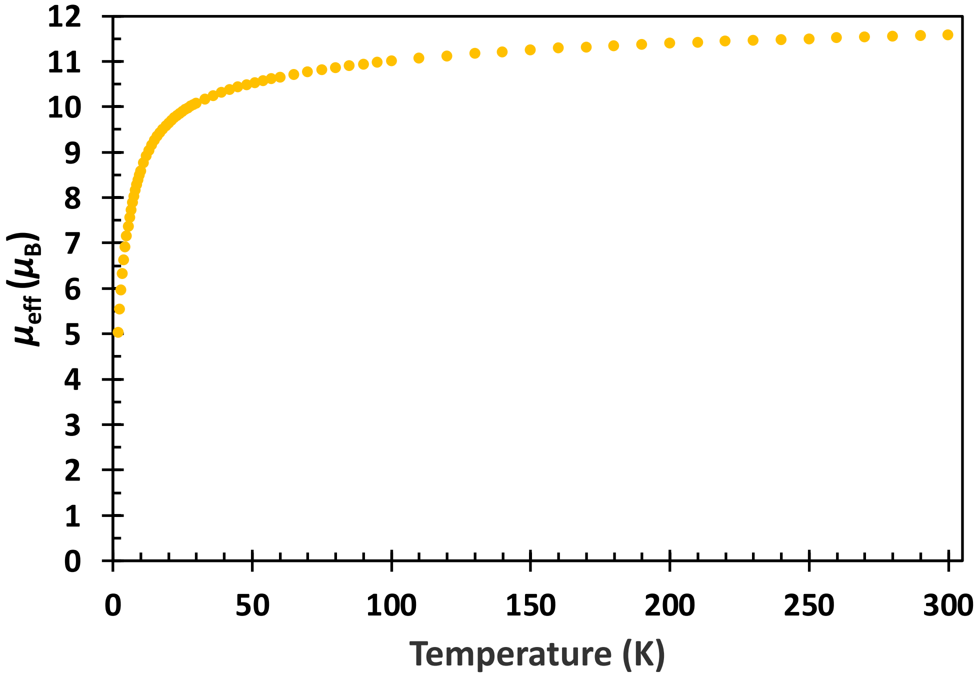

Figure S50. Temperature dependent SQUID magnetisation data (per complex) for **1** plotted as a function of μ_{eff} vs. temperature, measured at 1 T.

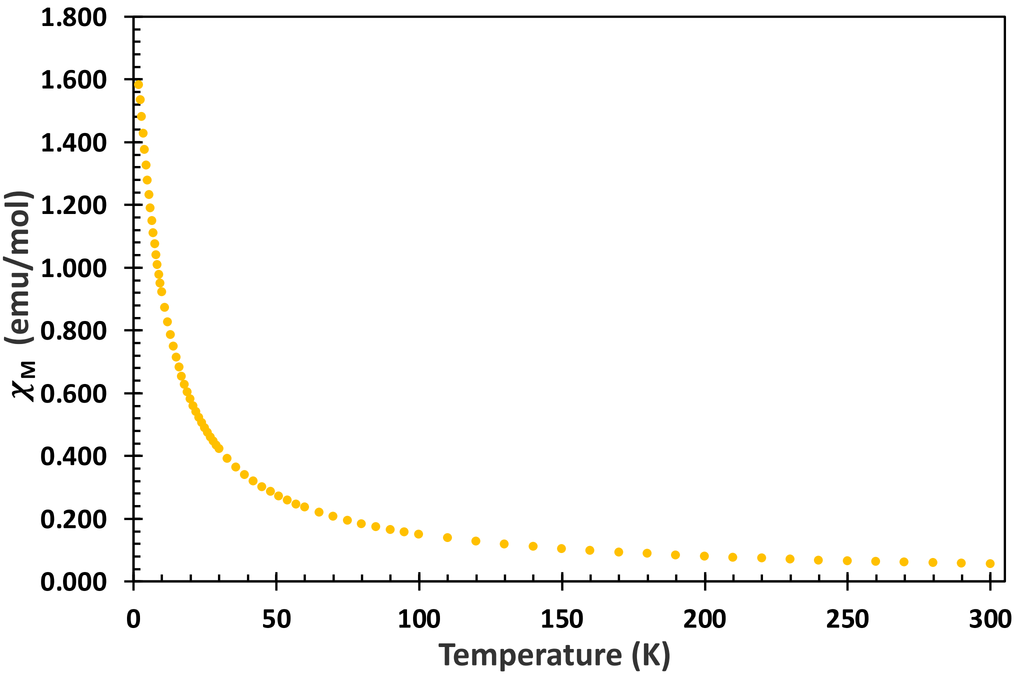

Figure S51. Temperature dependent SQUID magnetisation data (per complex) for **1** plotted as a function of χ_M (molar magnetic susceptibility) vs. temperature, measured at 1 T.

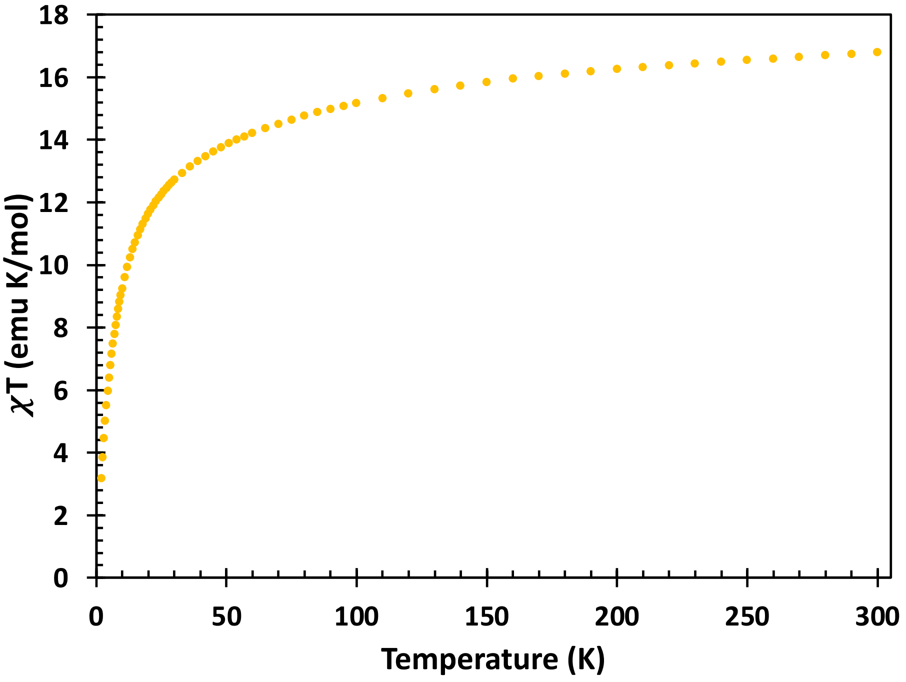

Figure S52. Temperature dependent SQUID magnetisation data (per complex) for **1** plotted as a function of χT vs. temperature, measured at 1 T.

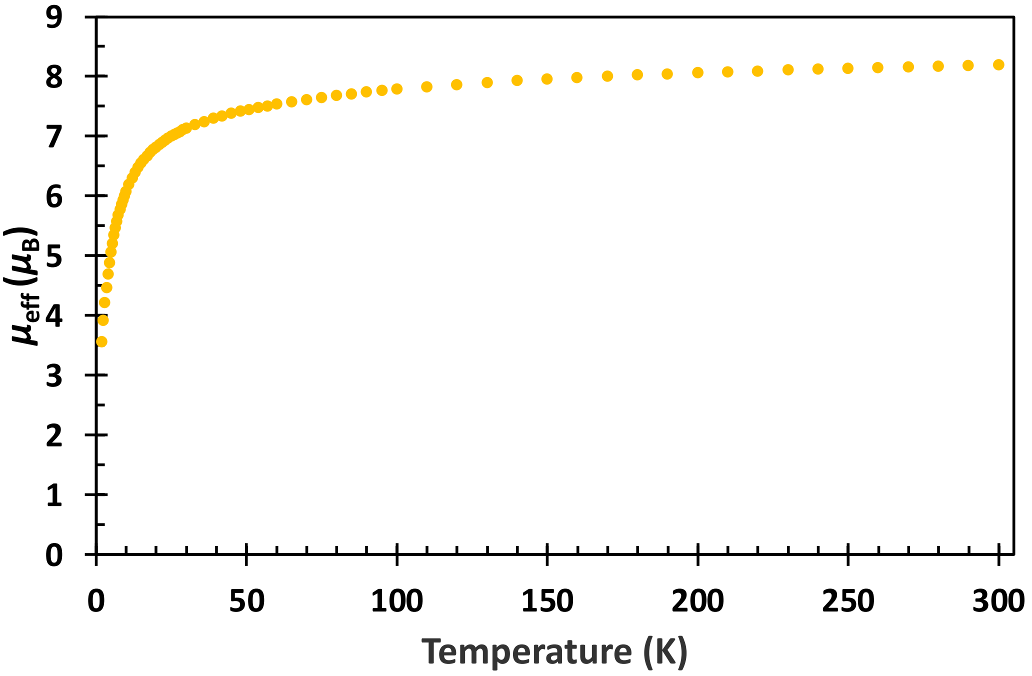

Figure S53. Temperature dependent SQUID magnetisation data (per Tm ion) for **1** plotted as a function of μ_{eff} vs. temperature, measured at 1 T.

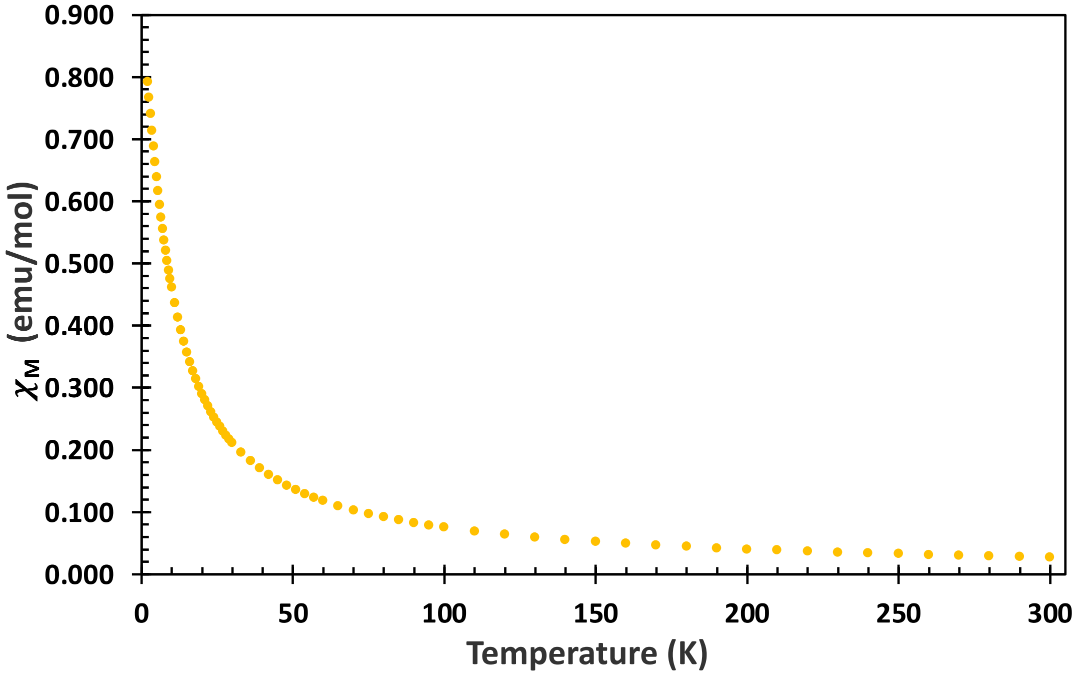

Figure S54. Temperature dependent SQUID magnetisation data (per Tm ion) for **1** plotted as a function of χ_M (molar magnetic susceptibility) vs. temperature, measured at 1 T.

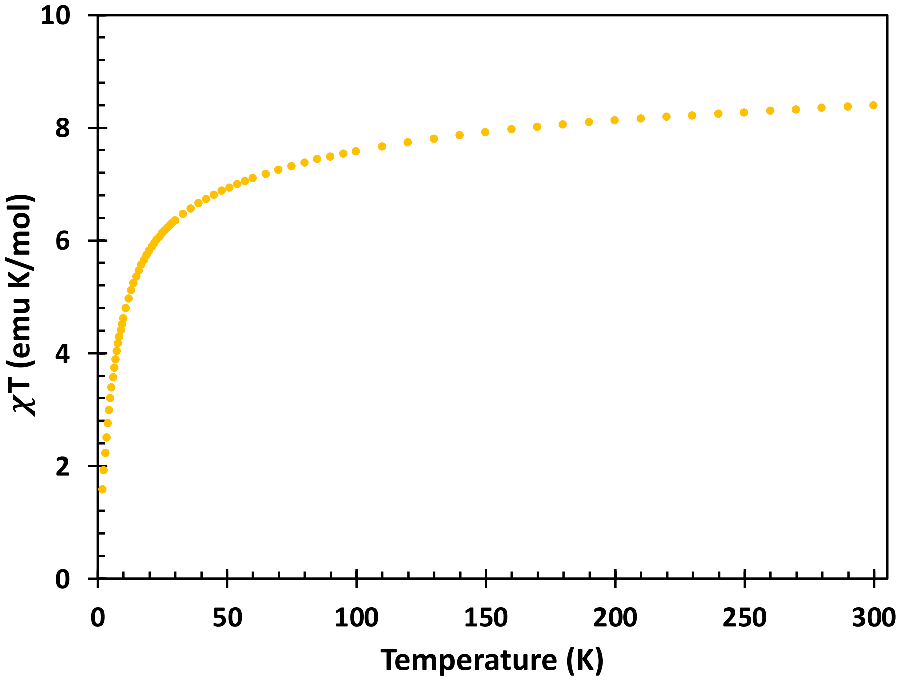

Figure S55. Temperature dependent SQUID magnetisation data (per Tm ion) for **1** plotted as a function of χT vs. temperature, measured at 1 T.

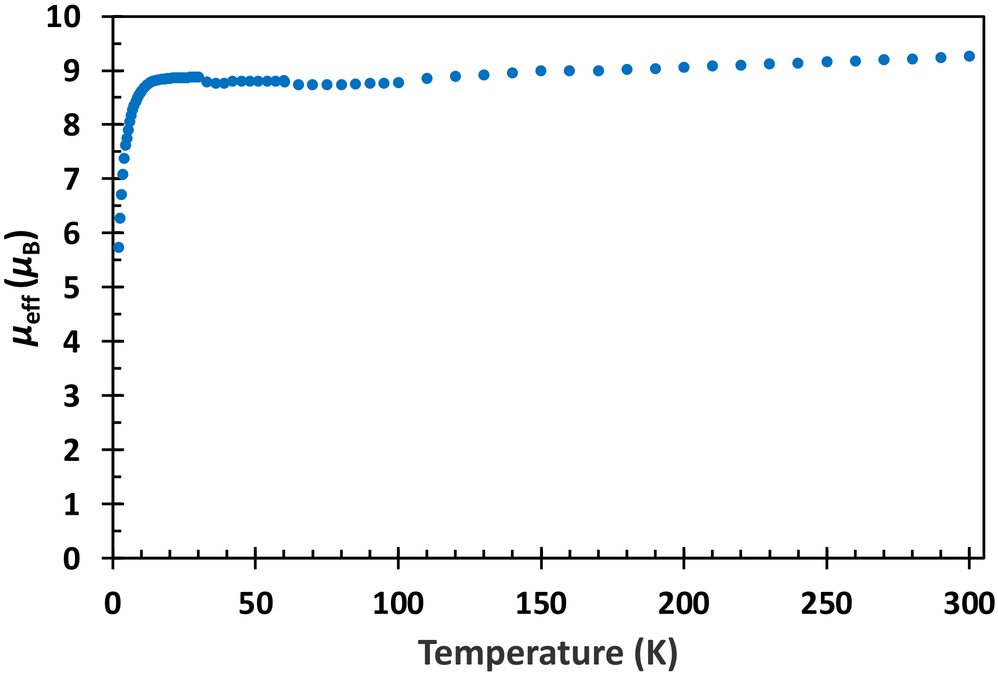

Figure S56. Temperature dependent SQUID magnetisation data (per complex) for **2** plotted as a function of μ_{eff} vs. temperature, measured at 1 T.

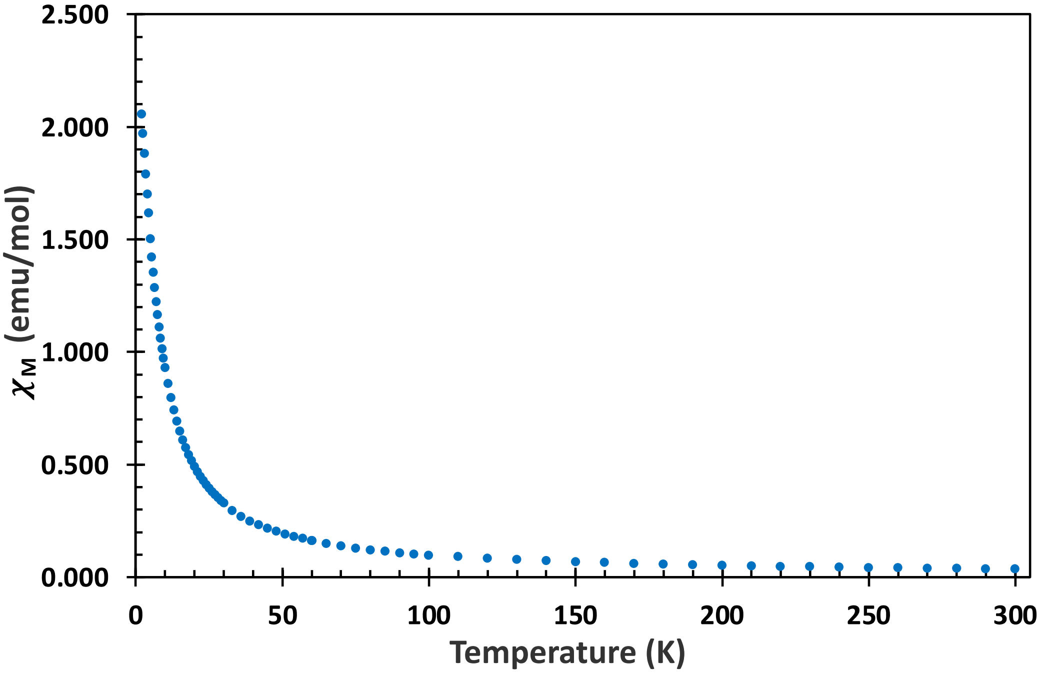

Figure S57. Temperature dependent SQUID magnetisation data (per complex) for **2** plotted as a function of χ_M (molar magnetic susceptibility) vs. temperature, measured at 1 T.

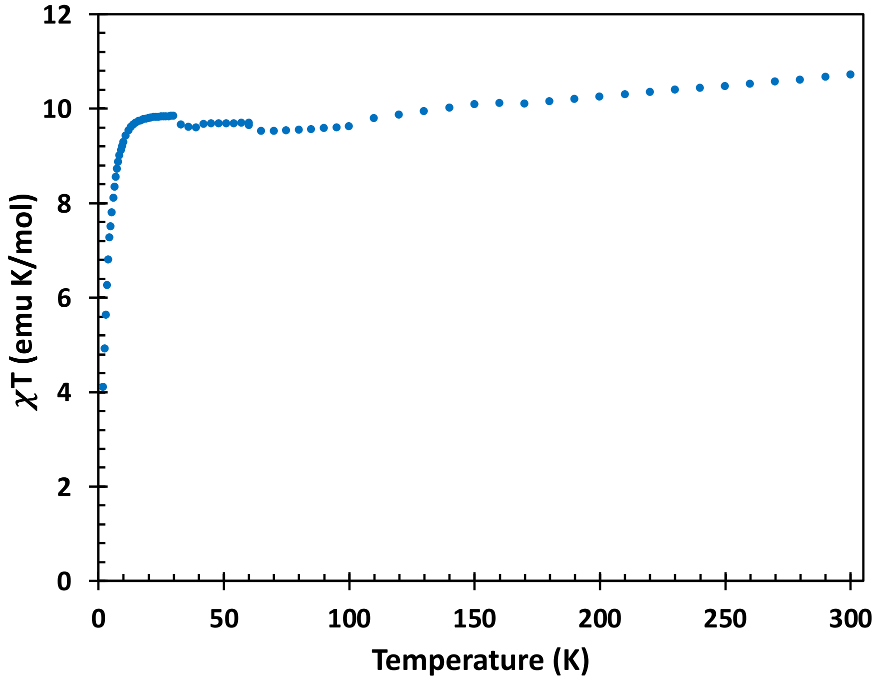

Figure S58. Temperature dependent SQUID magnetisation data (per complex) for **2** plotted as a function of χT vs. temperature, measured at 1 T.

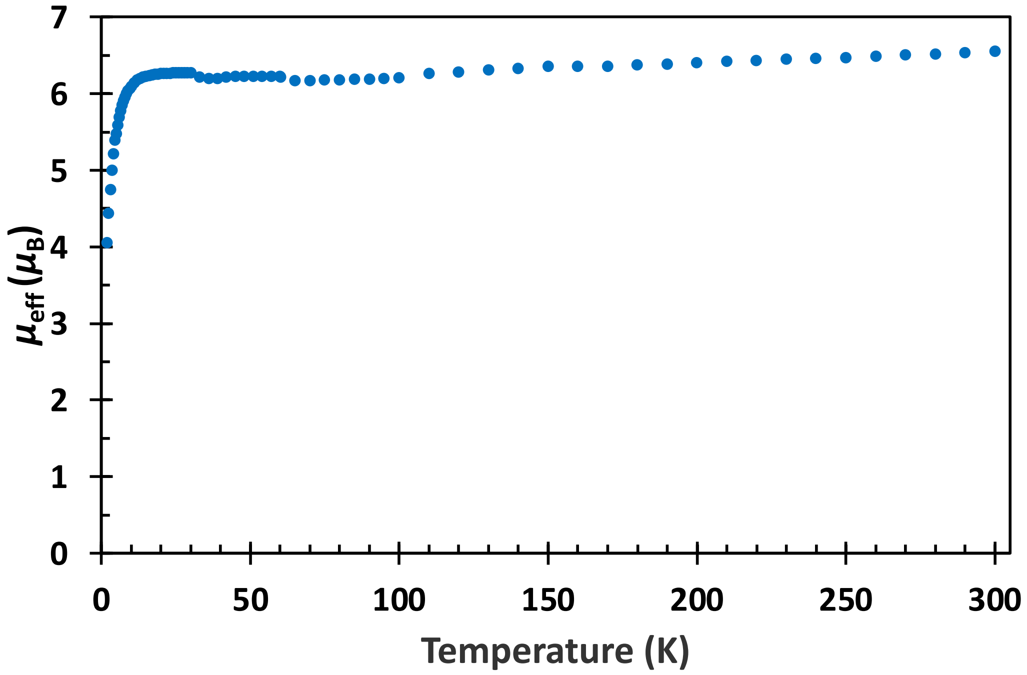

Figure S59. Temperature dependent SQUID magnetisation data (per Tm ion) for **2** plotted as a function of μ_{eff} vs. temperature, measured at 1 T.

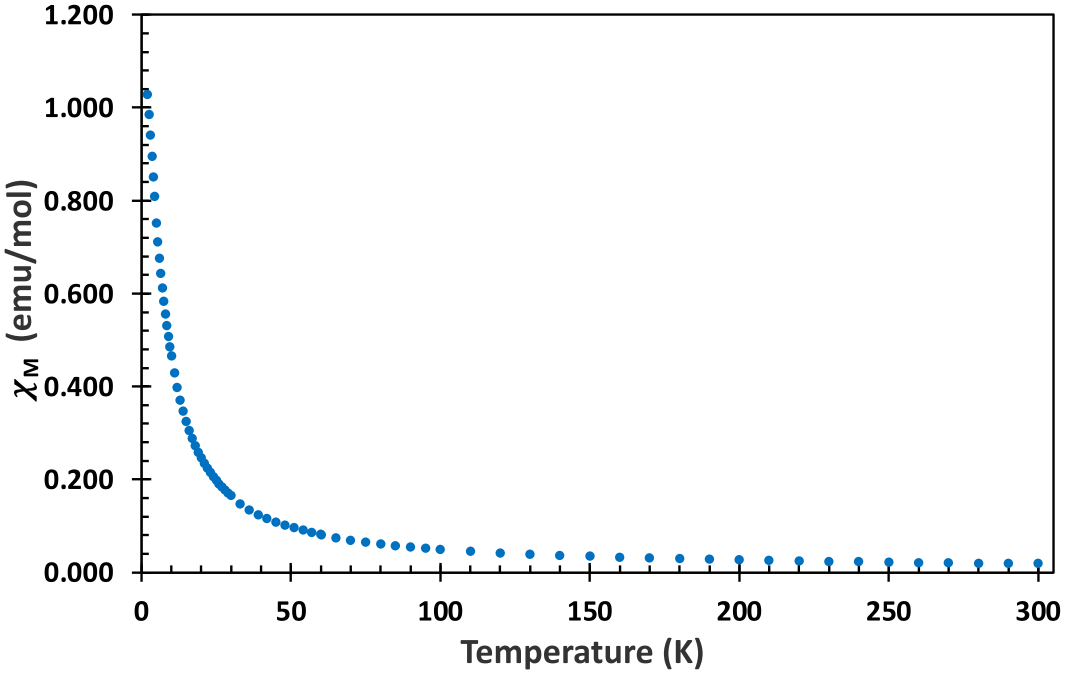

Figure S60. Temperature dependent SQUID magnetisation data (per Tm ion) for **2** plotted as a function of χ_M (molar magnetic susceptibility) vs. temperature, measured at 1 T.

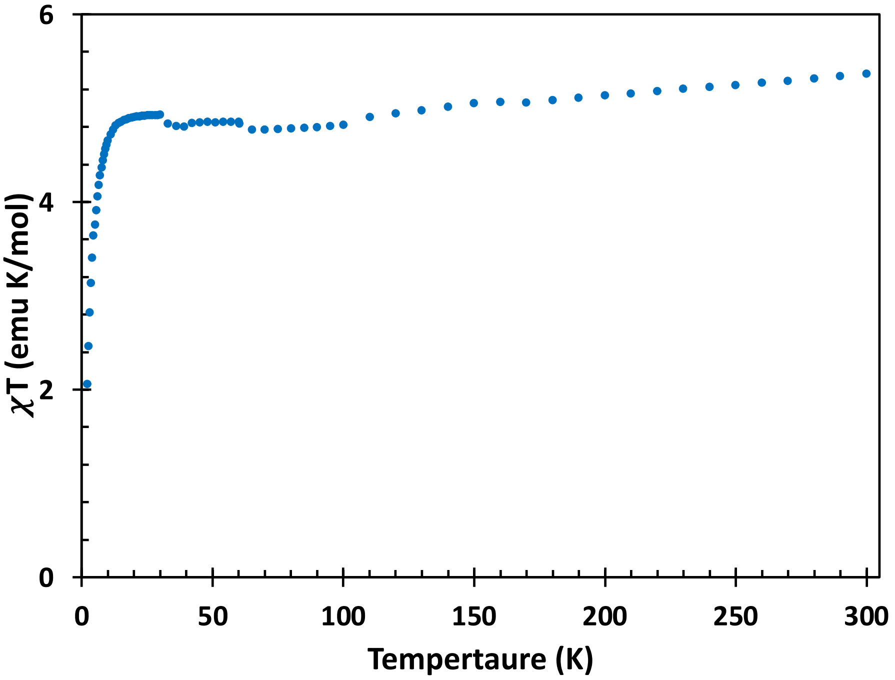

Figure S61. Temperature dependent SQUID magnetisation data (per Tm ion) for **2** plotted as a function of χT vs. temperature, measured at 1 T.

F. UV/Vis/NIR Spectra

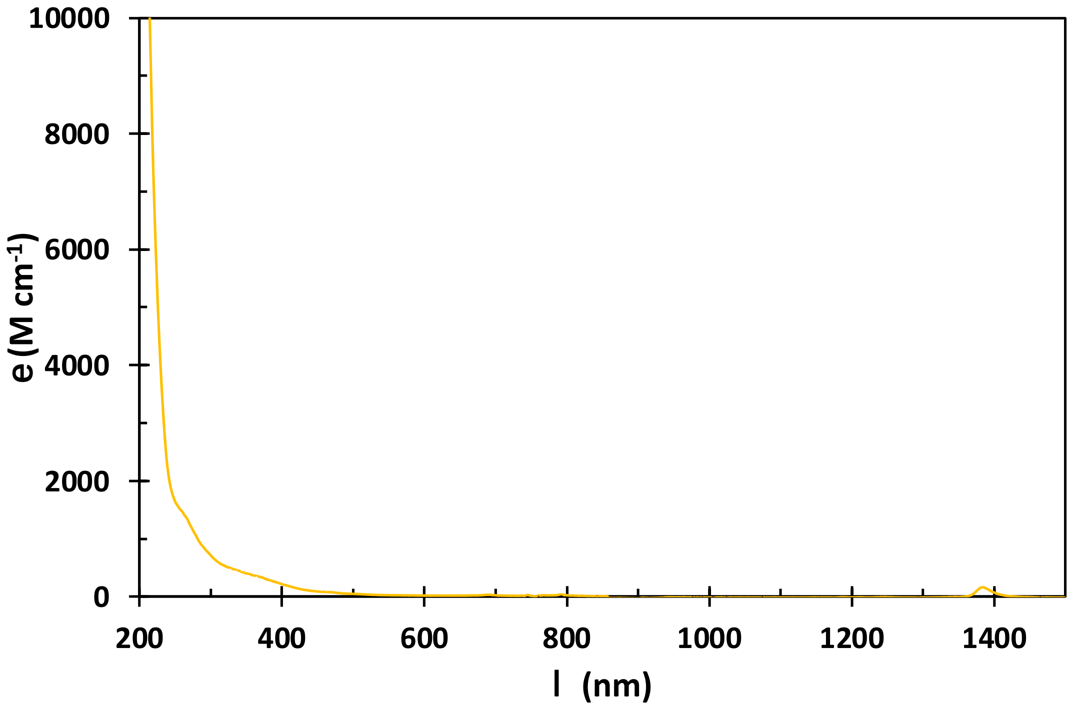

Figure S62. UV/Vis/NIR spectrum of **1** in diethyl ether (0.5 mM).

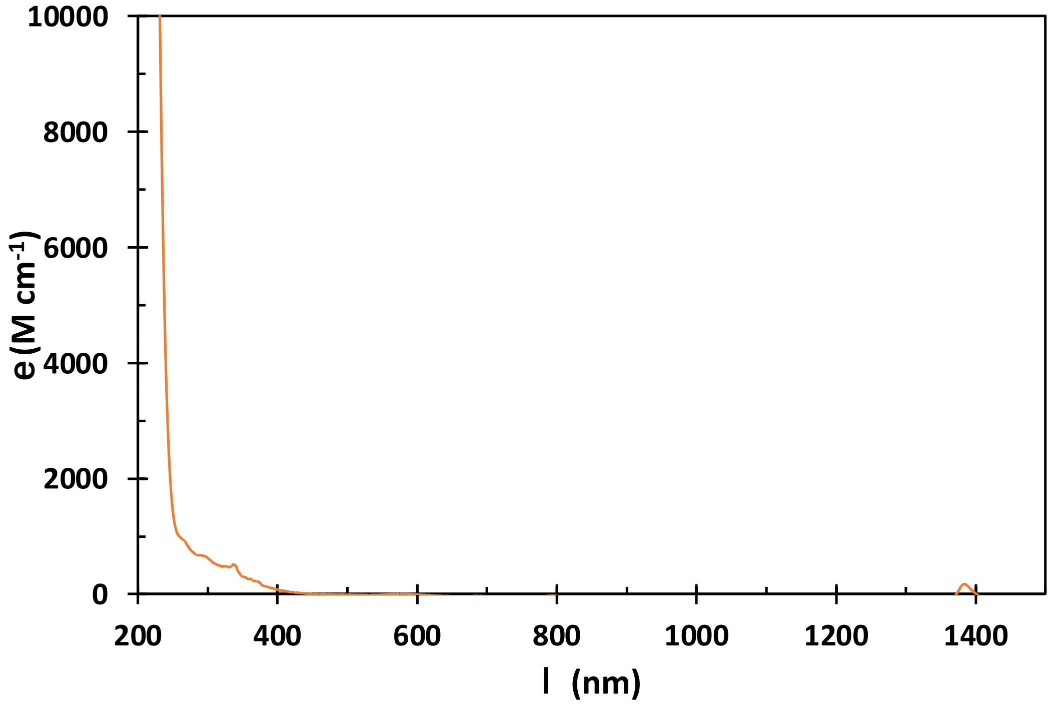

Figure S63. UV/Vis/NIR spectrum of **1-crypt** in diethyl ether (0.25 mM).

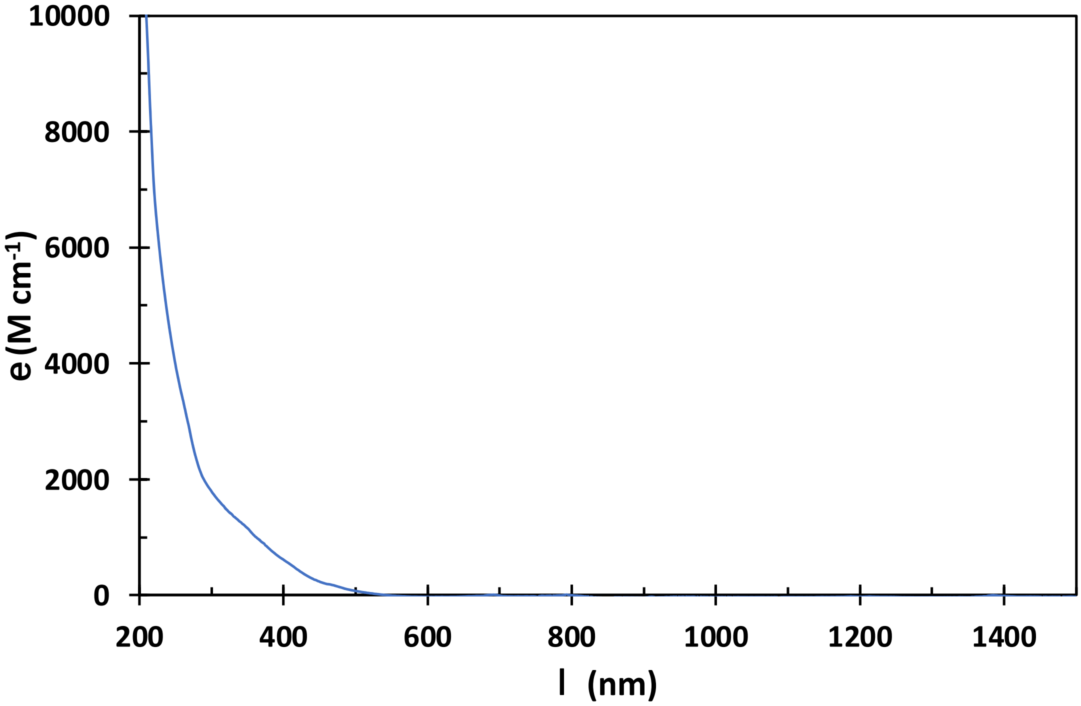

Figure S64. UV/Vis/NIR spectrum of **2** in diethyl ether (0.5 mM).

G. IR Spectra

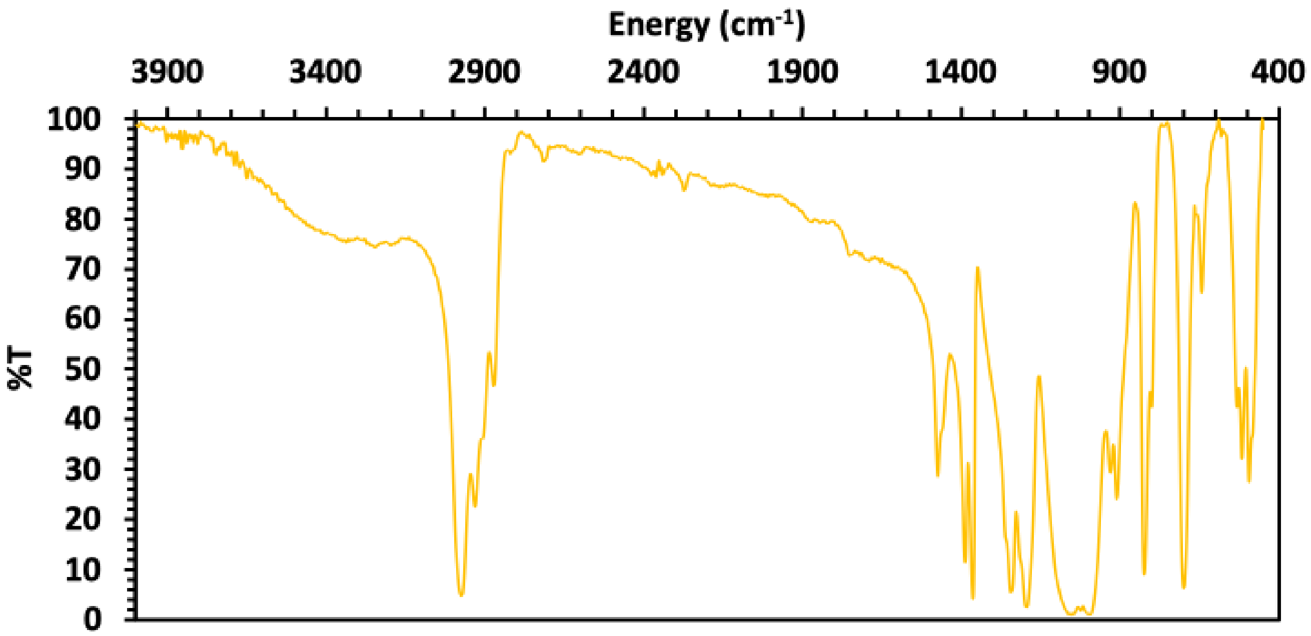

Figure S65. IR spectrum of isolated complex **1** in KBr pill measured at 25 °C.

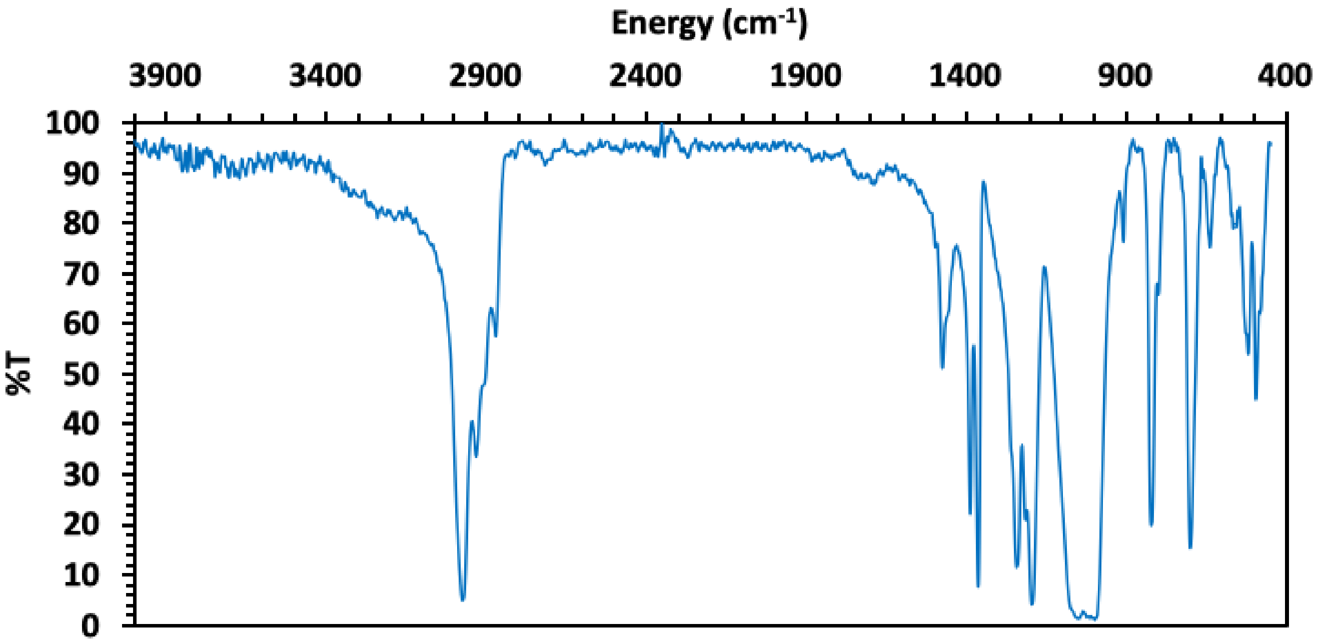

Figure S66. IR spectrum of isolated complex **2** in KBr pill measured at 25 °C.

H. Raman Spectra

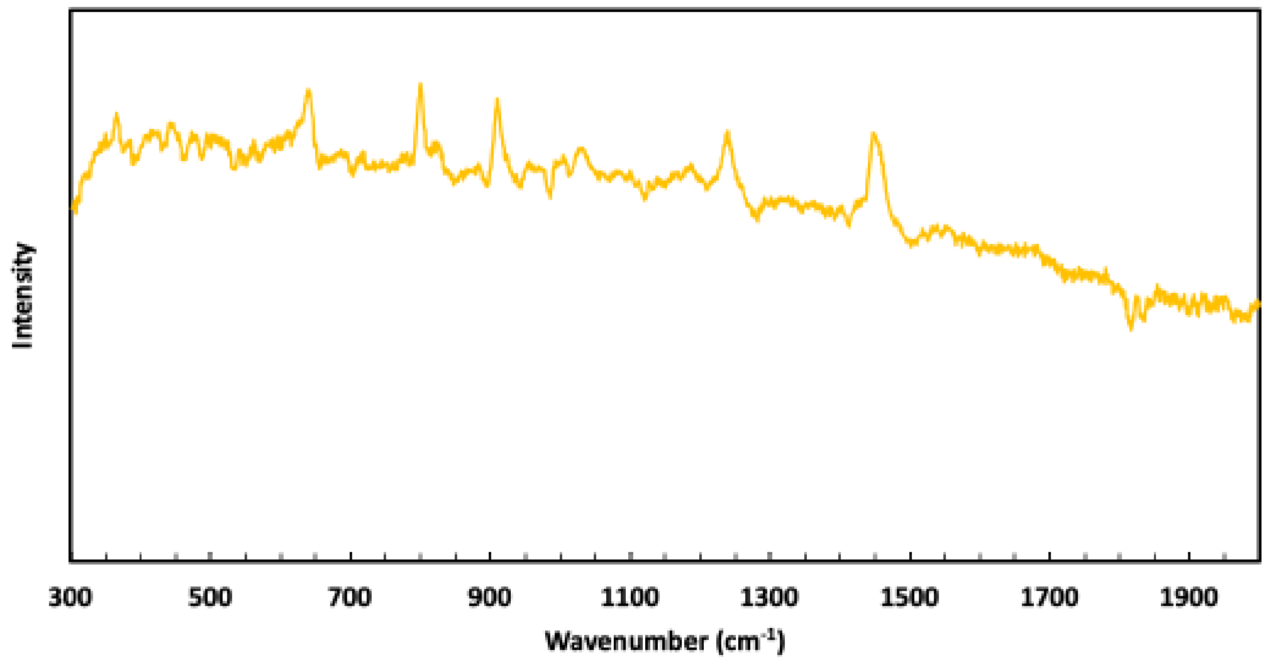

Figure S67. Raman spectrum of isolated complex **1** measured at 25 °C .

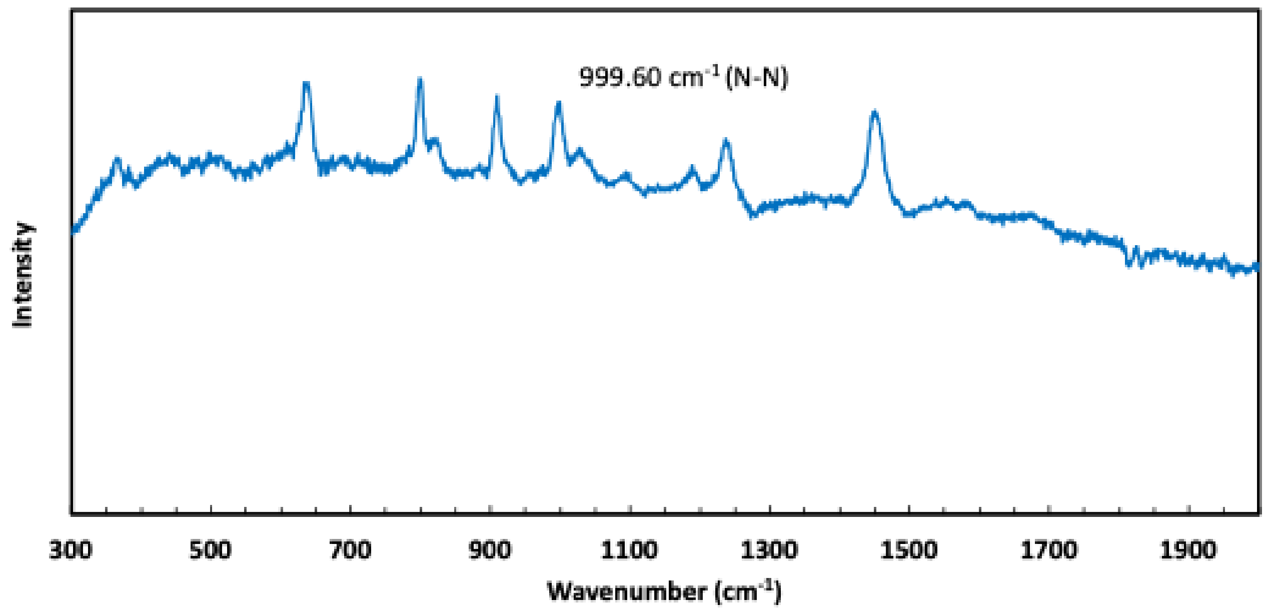

Figure S68. Raman spectrum of isolated complex **2** measured at 25 °C.

I. Computational Details

The optimization of three different spin states for uranium complexes were carried out by employing DFT hybrid functional (B3PW91) along with small core pseudopotential Stuggart basis set for thulium and silicon atoms with additional polarization functions for silicon atoms.[17–22] Pople basis sets (6-31G**) were employed for the rest of the atoms.[23,24] Dispersion corrections were included in our calculations by employing D3 version of Grimme's dispersion with Becke-Johnson damping.[25] All the calculations were performed using Gaussian 16 suite of programs.[26]

Table S4. Computed natural charges for selected atoms in complex **1** (S=2)

Atom label	Natural charges
Tm1	1.95306
K2	0.96207
O6	-1.35304
O7	-1.36930
O8	-1.37175
N135	-0.60247
N136	-0.60783
Tm137	1.95608
K138	0.96291
O142	-1.35297
O143	-1.37085
O144	-1.36961

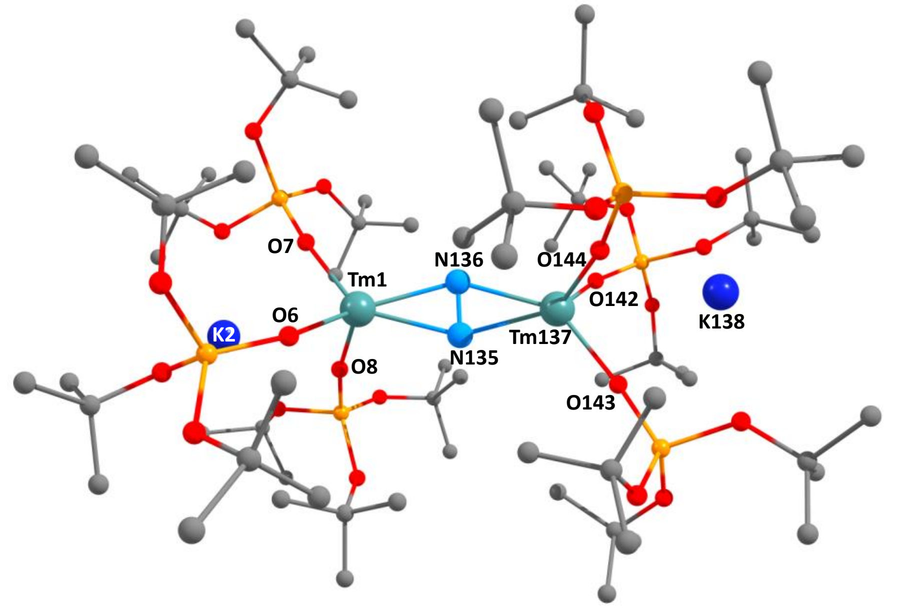

Table S5. Computed Wiberg bond indices between selected atoms in complex **1** (S=2)

Atom label	Wiberg bond index	Atom label	Wiberg bond index	Atom label	Wiberg bond index	Atom label	Wiberg bond index
Tm1	0.0000	Tm1	0.0000	Tm1	0.0000	Tm1	0.0000
O6	0.3520	O7	0.3297	O8	0.3493	N135	0.3146
Atom label	Wiberg bond index	Atom label	Wiberg bond index	Atom label	Wiberg bond index	Atom label	Wiberg bond index
Tm1	0.0000	N135	0.0000	Tm137	0.0000	Tm137	0.0000
N136	0.3187	N136	1.9593	N135	0.3094	N136	0.3058
Atom label	Wiberg bond index	Atom label	Wiberg bond index	Atom label	Wiberg bond index		
Tm137	0.0000	Tm137	0.0000	Tm137	0.0000		
N142	0.3525	N143	0.3326	N144	0.3580		

NBO analysis (Alpha Molecular orbitals, AMO) of occupancy, bond orbital/coefficients/hybrids for nitrogen atoms in complex **1** (S=2)
(0.93953) LP (1) N135
s(68.99%)p 0.45(30.95%)d 0.00(0.06%)
(0.91175) LP (2) N135
s(0.22%)p99.99(99.65%)d 0.60(0.13%)
(0.93946) LP (1) N136
s(69.14%)p 0.45(30.80%)d 0.00(0.06%)
(0.91210) LP (2) N136
s(0.11%)p99.99(99.76%)d 1.21(0.13%)
(0.98357) BD (1) N135- N136
(50.01%) 0.7072* N135 s(31.45%)p 2.17(68.21%)d 0.01(0.34%)
(49.99%) 0.7070* N136 s(31.42%)p 2.17(68.24%)d 0.01(0.34%)

(0.96511) BD (2) N135- N136
(49.85%) 0.7060* N135 s(0.00%)p 1.00(99.63%)d 0.00(0.37%
(50.15%) 0.7082* N136 s(0.00%)p 1.00(99.64%)d 0.00(0.36%)

Table S6. NBO Second order perturbation analysis (AMO) for complex **1** for S=2

Donor NBO	Acceptor NBO	E(2) kcal/mol
(0.95998) LP (1) O 7<br>s(35.85%)p 1.79(64.15%)d 0.00(0.01%)	(0.12978) LV (1)Tm 1<br>s(0.01%)p 1.00(0.65%)d99.99(99.20%)f 0.16(0.11%)g 0.05(0.03%)	15.19
(0.95998) LP (1) O 7<br>s(35.85%)p 1.79(64.15%)d 0.00(0.01%)	(0.06477) LV (5)Tm 1<br>s(0.55%)p 0.26(0.14%)d99.99(99.09%)f 0.34(0.19%)g 0.05(0.03%)	3.75
(0.93044) LP (2) O 7<br>s(0.19%)p99.99(99.78%)d 0.13(0.02%)	(0.09084) LV (2)Tm 1<br>s(4.93%)p 0.07(0.35%)d19.19(94.62%)f 0.01(0.06%)g 0.01(0.03%	3.34
(0.92816) LP (3) O 7<br>s(0.12%)p99.99(99.85%)d 0.20(0.03%)	(0.06477) LV (5)Tm 1<br>s(0.55%)p 0.26(0.14%)d99.99(99.09%)f 0.34(0.19%)g 0.05(0.03%)	4.49
(0.97918) BD (1)Si 3- O 7<br>(12.06%) 0.3473*Si 3 s(24.56%)p 3.03(74.46%)d 0.04(0.98%)<br>(87.94%) 0.9378* O 7 s(63.77%)p 0.57(36.10%)d 0.00(0.13%)	(0.12978) LV (1)Tm 1<br>s(0.01%)p 1.00(0.65%)d99.99(99.20%)f 0.16(0.11%)g 0.05(0.03%)	7.58
(0.95998) LP (1) O 7<br>s(35.85%)p 1.79(64.15%)d 0.00(0.01%)	(0.02902) LV (1) K 2<br>s(99.87%)p 0.00(0.08%)d 0.00(0.05%)	1.44
(0.95817) LP (1) O 6<br>s(37.02%)p 1.70(62.97%)d 0.00(0.01%)	(0.12978) LV (1)Tm 1<br>s(0.01%)p 1.00(0.65%)d99.99(99.20%)f 0.16(0.11%)g 0.05(0.03%)	5.79
(0.95817) LP (1) O 6<br>s(37.02%)p 1.70(62.97%)d 0.00(0.01%)	(0.07143) LV (3)Tm 1<br>s(0.03%)p 8.61(0.24%)d99.99(99.63%)f 2.00(0.06%)g 1.30(0.04%)	3.86
(0.95817) LP (1) O 6<br>s(37.02%)p 1.70(62.97%)d 0.00(0.01%)	(0.06791) LV (4)Tm 1<br>s(0.01%)p 1.00(0.73%)d99.99(98.96%)f 0.34(0.25%)g 0.06(0.05%)	11.96
(0.92852) LP (2) O 6<br>s(1.36%)p72.73(98.61%)d 0.02(0.03%)	(0.09084) LV (2)Tm 1<br>s(4.93%)p 0.07(0.35%)d19.19(94.62%)f 0.01(0.06%)g 0.01(0.03%	5.17
(0.92609) LP (3) O 6<br>s(0.83%)p99.99(99.14%)d 0.04(0.03%)	(0.07143) LV (3)Tm 1<br>s(0.03%)p 8.61(0.24%)d99.99(99.63%)f 2.00(0.06%)g 1.30(0.04%)	5.46
(0.97766) BD (1)Si 4- O 6<br>(12.25%) 0.3501*Si 4 s(24.54%)p 3.03(74.45%)d 0.04(1.01%)<br>(87.75%) 0.9367* O 6 s(60.72%)p 0.64(39.14%)d 0.00(0.13%)	(0.06791) LV (4)Tm 1<br>s(0.01%)p 1.00(0.73%)d99.99(98.96%)f 0.34(0.25%)g 0.06(0.05%)	5.62
(0.95817) LP (1) O 6<br>s(37.02%)p 1.70(62.97%)d 0.00(0.01%)	(0.02902) LV (1) K 2<br>s(99.87%)p 0.00(0.08%)d 0.00(0.05%)	1.32
(0.95403) LP (1) O 8<br>s(34.71%)p 1.88(65.29%)d 0.00(0.00%)	(0.09084) LV (2)Tm 1<br>s(4.93%)p 0.07(0.35%)d19.19(94.62%)f 0.01(0.06%)g 0.01(0.03%	4.99
(0.95403) LP (1) O 8<br>s(34.71%)p 1.88(65.29%)d 0.00(0.00%)	(0.06791) LV (4)Tm 1<br>s(0.01%)p 1.00(0.73%)d99.99(98.96%)f 0.34(0.25%)g 0.06(0.05%)	16.73
(0.93420) LP (2) O 8<br>s(0.99%)p99.67(98.99%)d 0.02(0.02%)	(0.06477) LV (5)Tm 1<br>s(0.55%)p 0.26(0.14%)d99.99(99.09%)f 0.34(0.19%)g 0.05(0.03%)	3.47
(0.92904) LP (3) O 8<br>s(0.06%)p99.99(99.92%)d 0.32(0.02%)	(0.06477) LV (5)Tm 1	4.20

	s(0.55%)p 0.26(0.14%)d99.99(99.09%)f 0.34(0.19%)g 0.05(0.03%)	
(0.97969) BD (1)Si 5- O 8<br>(12.01%) 0.3465*Si 5 s(24.25%)p 3.08(74.76%)d 0.04(1.00%)<br>(87.99%) 0.9380* O 8 s(64.18%)p 0.56(35.71%)d 0.00(0.12%)	(0.06791) LV (4)Tm 1<br>s(0.01%)p 1.00(0.73%)d99.99(98.96%)f 0.34(0.25%)g 0.06(0.05%)	8.34
(0.95403) LP (1) O 8<br>s(34.71%)p 1.88(65.29%)d 0.00(0.00%)	(0.02902) LV (1) K 2<br>s(99.87%)p 0.00(0.08%)d 0.00(0.05%)	1.37
(0.93953) LP (1) N135<br>s(68.99%)p 0.45(30.95%)d 0.00(0.06%)	(0.12978) LV (1)Tm 1<br>s(0.01%)p 1.00(0.65%)d99.99(99.20%)f 0.16(0.11%)g 0.05(0.03%)	8.38
(0.93953) LP (1) N135<br>s(68.99%)p 0.45(30.95%)d 0.00(0.06%)	(0.09084) LV (2)Tm 1<br>s(4.93%)p 0.07(0.35%)d19.19(94.62%)f 0.01(0.06%)g 0.01(0.03%	11.26
(0.91175) LP (2) N135<br>s(0.22%)p99.99(99.65%)d 0.60(0.13%)	(0.12978) LV (1)Tm 1<br>s(0.01%)p 1.00(0.65%)d99.99(99.20%)f 0.16(0.11%)g 0.05(0.03%)	5.73
(0.93946) LP (1) N136<br>s(69.14%)p 0.45(30.80%)d 0.00(0.06%)	(0.12978) LV (1)Tm 1<br>s(0.01%)p 1.00(0.65%)d99.99(99.20%)f 0.16(0.11%)g 0.05(0.03%)	6.73
(0.93946) LP (1) N136<br>s(69.14%)p 0.45(30.80%)d 0.00(0.06%)	(0.09084) LV (2)Tm 1<br>s(4.93%)p 0.07(0.35%)d19.19(94.62%)f 0.01(0.06%)g 0.01(0.03%	9.87
(0.91210) LP (2) N136<br>s(0.11%)p99.99(99.76%)d 1.21(0.13%)	(0.12978) LV (1)Tm 1<br>s(0.01%)p 1.00(0.65%)d99.99(99.20%)f 0.16(0.11%)g 0.05(0.03%)	7.78
(0.98357) BD (1) N135- N136<br>(50.01%) 0.7072* N135 s(31.45%)p 2.17(68.21%)d 0.01(0.34%)<br>(49.99%) 0.7070* N136 s(31.42%)p 2.17(68.24%)d 0.01(0.34%)	(0.09084) LV (2)Tm 1<br>s(4.93%)p 0.07(0.35%)d19.19(94.62%)f 0.01(0.06%)g 0.01(0.03%	12.44
(0.96511) BD (2) N135- N136<br>(49.85%) 0.7060* N135 s(0.00%)p 1.00(99.63%)d 0.00(0.37%)<br>(50.15%) 0.7082* N136 s(0.00%)p 1.00(99.64%)d 0.00(0.36%)	(0.07143) LV (3)Tm 1<br>s(0.03%)p 8.61(0.24%)d99.99(99.63%)f 2.00(0.06%)g 1.30(0.04%)	4.29
(0.93953) LP (1) N135<br>s(68.99%)p 0.45(30.95%)d 0.00(0.06%)	(0.13175) LV (1)Tm137<br>s(0.01%)p 1.00(0.77%)d99.99(99.09%)f 0.13(0.10%)g 0.05(0.04%)	6.96
(0.93953) LP (1) N135<br>s(68.99%)p 0.45(30.95%)d 0.00(0.06%)	(0.09136) LV (2)Tm137<br>s(5.27%)p 0.07(0.35%)d17.88(94.27%)f 0.01(0.07%)g 0.01(0.03%)	9.94
(0.91175) LP (2) N135<br>s(0.22%)p99.99(99.65%)d 0.60(0.13%)	(0.13175) LV (1)Tm137<br>s(0.01%)p 1.00(0.77%)d99.99(99.09%)f 0.13(0.10%)g 0.05(0.04%)	8.18
(0.93946) LP (1) N136<br>s(69.14%)p 0.45(30.80%)d 0.00(0.06%)	(0.13175) LV (1)Tm137<br>s(0.01%)p 1.00(0.77%)d99.99(99.09%)f 0.13(0.10%)g 0.05(0.04%)	8.00
(0.93946) LP (1) N136<br>s(69.14%)p 0.45(30.80%)d 0.00(0.06%)	(0.09136) LV (2)Tm137<br>s(5.27%)p 0.07(0.35%)d17.88(94.27%)f 0.01(0.07%)g 0.01(0.03%)	11.47
(0.91210) LP (2) N136<br>s(0.11%)p99.99(99.76%)d 1.21(0.13%)	(0.13175) LV (1)Tm137<br>s(0.01%)p 1.00(0.77%)d99.99(99.09%)f 0.13(0.10%)g 0.05(0.04%)	6.15
(0.98357) BD (1) N135- N136<br>(50.01%) 0.7072* N135 s(31.45%)p 2.17(68.21%)d 0.01(0.34%)<br>(49.99%) 0.7070* N136 s(31.42%)p 2.17(68.24%)d 0.01(0.34%)	(0.09136) LV (2)Tm137<br>s(5.27%)p 0.07(0.35%)d17.88(94.27%)f 0.01(0.07%)g 0.01(0.03%)	12.55

(0.96511) BD (2) N135- N136<br>(49.85%) 0.7060* N135 s(0.00%)p 1.00(99.63%)d 0.00(0.37%)<br>(50.15%) 0.7082* N136 s(0.00%)p 1.00(99.64%)d 0.00(0.36%)	(0.07204) LV (3)Tm137<br>s(0.05%)p 2.53(0.12%)d99.99(99.77%)f 0.67(0.03%)g 0.62(0.03%)	4.59
(0.96008) LP (1) O143<br>s(35.47%)p 1.82(64.52%)d 0.00(0.01%)	(0.13175) LV (1)Tm137<br>s(0.01%)p 1.00(0.77%)d99.99(99.09%)f 0.13(0.10%)g 0.05(0.04%)	15.74
(0.96008) LP (1) O143<br>s(35.47%)p 1.82(64.52%)d 0.00(0.01%)	(0.06608) LV (5)Tm137<br>s(0.52%)p 0.33(0.17%)d99.99(99.07%)f 0.38(0.20%)g 0.05(0.03%)	4.35
(0.93080) LP (2) O143<br>s(0.19%)p99.99(99.79%)d 0.13(0.02%)	(0.09136) LV (2)Tm137<br>s(5.27%)p 0.07(0.35%)d17.88(94.27%)f 0.01(0.07%)g 0.01(0.03%)	3.36
(0.92831) LP (3) O143<br>s(0.10%)p99.99(99.88%)d 0.24(0.02%)	(0.07204) LV (3)Tm137<br>s(0.05%)p 2.53(0.12%)d99.99(99.77%)f 0.67(0.03%)g 0.62(0.03%)	4.16
346. BD (1)Si139- O143	(0.13175) LV (1)Tm137<br>s(0.01%)p 1.00(0.77%)d99.99(99.09%)f 0.13(0.10%)g 0.05(0.04%)	7.97
(0.96008) LP (1) O143<br>s(35.47%)p 1.82(64.52%)d 0.00(0.01%)	(0.02872) LV (1) K13<br>s(99.88%)p 0.00(0.08%)d 0.00(0.05%)	1.40
(0.95872) LP (1) O142<br>s(36.75%)p 1.72(63.24%)d 0.00(0.01%)	(0.13175) LV (1)Tm137<br>s(0.01%)p 1.00(0.77%)d99.99(99.09%)f 0.13(0.10%)g 0.05(0.04%)	7.71
(0.95872) LP (1) O142<br>s(36.75%)p 1.72(63.24%)d 0.00(0.01%)	(0.06786) LV (4)Tm137<br>s(0.13%)p 5.84(0.76%)d99.99(98.81%)f 1.87(0.25%)g 0.40(0.05%)	8.78
(0.95872) LP (1) O142<br>s(36.75%)p 1.72(63.24%)d 0.00(0.01%)	(0.06608) LV (5)Tm137<br>s(0.52%)p 0.33(0.17%)d99.99(99.07%)f 0.38(0.20%)g 0.05(0.03%)	5.45
(0.92899) LP (2) O142<br>s(1.68%)p58.55(98.29%)d 0.02(0.03%)	(0.09136) LV (2)Tm137<br>s(5.27%)p 0.07(0.35%)d17.88(94.27%)f 0.01(0.07%)g 0.01(0.03%)	4.78
(0.92597) LP (3) O142<br>s(0.46%)p99.99(99.51%)d 0.07(0.03%)	(0.07204) LV (3)Tm137<br>s(0.05%)p 2.53(0.12%)d99.99(99.77%)f 0.67(0.03%)g 0.62(0.03%)	5.21
(0.97770) BD (1)Si140- O142<br>(12.26%) 0.3501*Si140 s(24.56%)p 3.03(74.46%)d 0.04(0.99%)<br>(87.74%) 0.9367* O142 s(61.04%)p 0.64(38.82%)d 0.00(0.13%)	(0.13175) LV (1)Tm137<br>s(0.01%)p 1.00(0.77%)d99.99(99.09%)f 0.13(0.10%)g 0.05(0.04%)	5.05
(0.97770) BD (1)Si140- O142<br>(12.26%) 0.3501*Si140 s(24.56%)p 3.03(74.46%)d 0.04(0.99%)<br>(87.74%) 0.9367* O142 s(61.04%)p 0.64(38.82%)d 0.00(0.13%)	(0.06786) LV (4)Tm137<br>s(0.13%)p 5.84(0.76%)d99.99(98.81%)f 1.87(0.25%)g 0.40(0.05%)	4.18
(0.95384) LP (1) O144<br>s(34.11%)p 1.93(65.89%)d 0.00(0.00%)	(0.09136) LV (2)Tm137<br>s(5.27%)p 0.07(0.35%)d17.88(94.27%)f 0.01(0.07%)g 0.01(0.03%)	6.22
(0.95384) LP (1) O144<br>s(34.11%)p 1.93(65.89%)d 0.00(0.00%)	(0.06786) LV (4)Tm137<br>s(0.13%)p 5.84(0.76%)d99.99(98.81%)f 1.87(0.25%)g 0.40(0.05%)	16.62
(0.93369) LP (2) O144<br>s(1.19%)p82.96(98.79%)d 0.01(0.02%)	(0.07204) LV (3)Tm137<br>s(0.05%)p 2.53(0.12%)d99.99(99.77%)f 0.67(0.03%)g 0.62(0.03%)	5.07
(0.92897) LP (3) O144<br>s(0.16%)p99.99(99.82%)d 0.11(0.02%)	(0.06608) LV (5)Tm137<br>s(0.52%)p 0.33(0.17%)d99.99(99.07%)f 0.38(0.20%)g 0.05(0.03%)	5.70
(0.97956) BD (1)Si141- O144	(0.06786) LV (4)Tm137	8.30

(12.01%) 0.3465*Si141 s(24.13%)p 3.10(74.87%)d 0.04(1.01%) (87.99%) 0.9381* O144 s(64.47%)p 0.55(35.42%)d 0.00(0.11%)	s(0.13%)p 5.84(0.76%)d99.99(98.81%)f 1.87(0.25%)g 0.40(0.05%)	
(0.95384) LP (1) O144 s(34.11%)p 1.93(65.89%)d 0.00(0.00%)	(0.02872) LV (1) K13 s(99.88%)p 0.00(0.08%)d 0.00(0.05%)	1.27

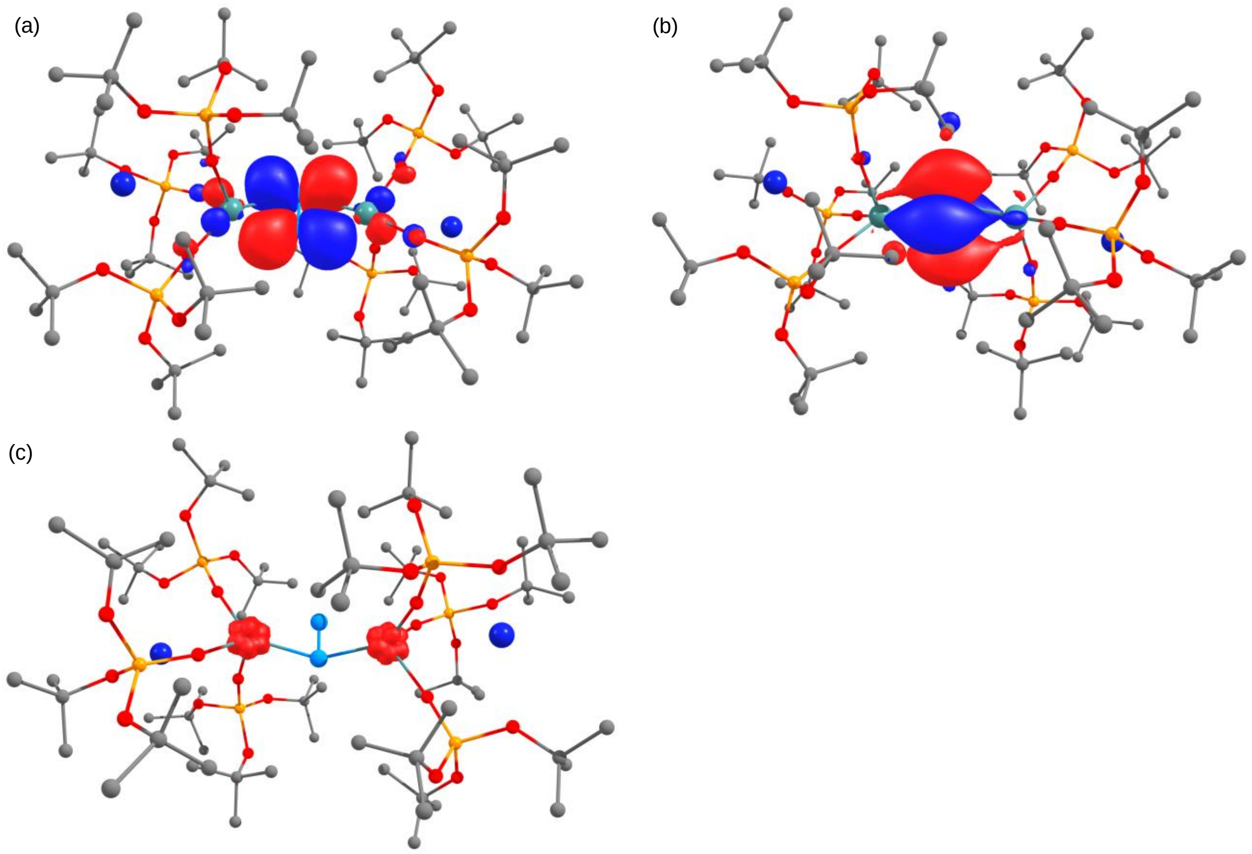

Figure S69. Computed MOs (AMO) for complex **1** (s=2). (a)HOMO (b)LUMO (c)spin density plot (1.93 spin density on each thulium atoms).

Table S7. Computed natural charges for selected atoms in complex **2** (S=5/2)

Atom labels	Natural charges	
Tm1	1.87353	
Tm2	1.88374	
K3	0.95334	
K4	0.95127	
K5	0.95856	
O12	-1.35314	
O16	-1.34795	
O17	-1.35132	
O19	-1.36077	
O22	-1.35289	
O24	-1.35978	
N31	-0.97793	

N35	-0.97477	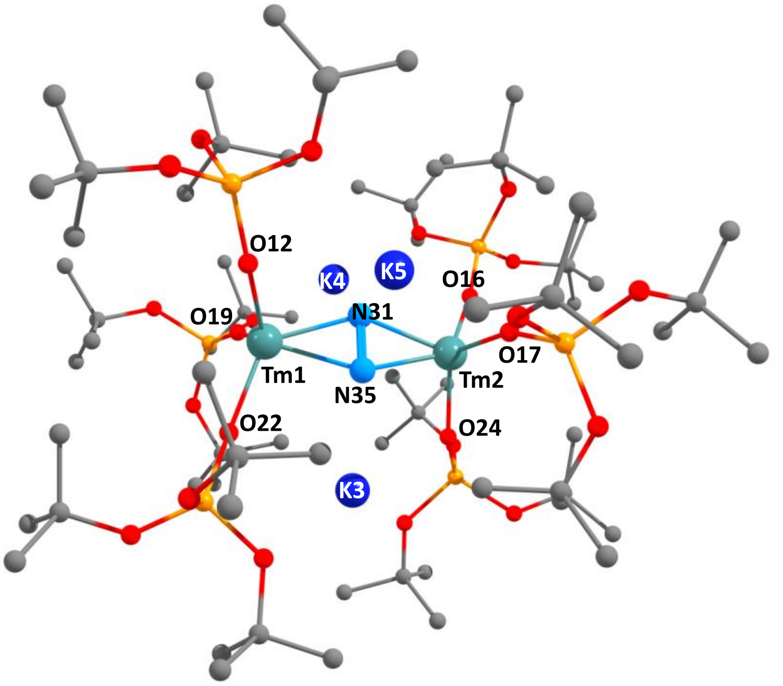

Table S8. Computed Wiberg bond indices between selected atoms in complex **2** (S=5/2)

Atom label	Wiberg bond index	Atom label	Wiberg bond index	Atom label	Wiberg bond index	Atom label	Wiberg bond index
Tm1	0.0000	Tm1	0.0000	Tm1	0.0000	Tm1	0.0000
O12	0.3320	O19	0.3175	O22	0.3211	N31	0.3790
Atom label	Wiberg bond index	Atom label	Wiberg bond index	Atom label	Wiberg bond index	Atom label	Wiberg bond index
Tm1	0.0000	N31	0.0000	Tm2	0.0000	Tm2	0.0000
N35	0.4271	N35	1.4091	O16	0.3230	O17	0.3368
Atom label	Wiberg bond index	Atom label	Wiberg bond index	Atom label	Wiberg bond index		
Tm2	0.0000	Tm2	0.0000	Tm2	0.0000		
O24	0.3186	N31	0.4157	N35	0.3721		

NBO analysis (AMO) of occupancy, bond orbital/coefficients/hybrids for nitrogen and thulium atoms in complex **2** (S=5/2)
(0.93194) LP (1) N 31
s(0.84%)p99.99(99.13%)d 0.05(0.04%)
(0.93098) LP (1) N 35
s(0.58%)p99.99(99.38%)d 0.07(0.04%)
(0.97308) BD (1)Tm 1- N 31
(5.88%) 0.2426*Tm 1 s(4.80%)p 0.08(0.37%)d19.68(94.46%) f 0.06(0.28%)g 0.02(0.09%)
(94.12%) 0.9701* N 31 s(41.52%)p 1.41(58.42%)d 0.00(0.05%)
(0.97192) BD (1)Tm 1- N 35
(6.57%) 0.2564*Tm 1 s(4.54%)p 0.03(0.12%)d20.94(94.96%) f 0.06(0.28%)g 0.02(0.10%)
(93.43%) 0.9666* N 35 s(40.37%)p 1.48(59.57%)d 0.00(0.06%)
(0.97203) BD (1)Tm 2- N 31
(6.48%) 0.2546*Tm 2 s(5.76%)p 0.02(0.11%)d16.27(93.77%) f 0.05(0.26%)g 0.02(0.10%)
(93.52%) 0.9670* N 31 s(40.81%)p 1.45(59.13%)d 0.00(0.06%)
(0.97392) BD (1)Tm 2- N 35
(5.80%) 0.2408*Tm 2 s(5.83%)p 0.07(0.38%)d16.01(93.39%) f 0.05(0.29%)g 0.02(0.10%)
(94.20%) 0.9706* N 35 s(42.54%)p 1.35(57.41%)d 0.00(0.06%)

Table S9. NBO Second order perturbation analysis (AMO) for complex **2** (S=5/2)

Donor NBO	Acceptor NBO	E(2) kcal/mol
(0.93194) LP (1) N 31 s(0.84%)p99.99(99.13%)d 0.05(0.04%)	(0.07286) LV (3)Tm 1 s(0.05%)p 5.06(0.27%)d99.99(99.53%)f 1.98(0.11%)g 0.76(0.04%)	3.58
(0.93194) LP (1) N 31 s(0.84%)p99.99(99.13%)d 0.05(0.04%)	(0.10414) LV (1)Tm 2 s(0.15%)p 3.22(0.49%)d99.99(99.31%)f 0.23(0.03%)g 0.10(0.02%)	3.84
(0.93098) LP (1) N 35 s(0.58%)p99.99(99.38%)d 0.07(0.04%)	(0.10580) LV (1)Tm 1 s(0.02%)p29.83(0.49%)d99.99(99.45%) f 2.03(0.03%)g 0.89(0.01%)	4.30
(0.93098) LP (1) N 35 s(0.58%)p99.99(99.38%)d 0.07(0.04%)	(0.07286) LV (3)Tm 1 s(0.05%)p 5.06(0.27%)d99.99(99.53%)f 1.98(0.11%)g 0.76(0.04%)	3.20
(0.93098) LP (1) N 35 s(0.58%)p99.99(99.38%)d 0.07(0.04%)	(0.07777) LV (2)Tm 2 s(0.17%)p 0.63(0.10%)d99.99(99.65%)f 0.18(0.03%)g 0.27(0.04%)	3.95
(0.97308) BD (1)Tm 1- N 31 (5.88%) 0.2426*Tm 1 s(4.80%)p 0.08(0.37%)d19.68(94.46%) f 0.06(0.28%)g 0.02(0.09%)	(0.03231) LV (1) K 3 s(99.59%)p 0.00(0.34%)d 0.00(0.08%)	1.00
(0.97192) BD (1)Tm 1- N 35 (6.57%) 0.2564*Tm 1 s(4.54%)p 0.03(0.12%)d20.94(94.96%) f 0.06(0.28%)g 0.02(0.10%) (93.43%) 0.9666* N 35 s(40.37%)p 1.48(59.57%)d 0.00(0.06%)	(0.03231) LV (1) K 3 s(99.59%)p 0.00(0.34%)d 0.00(0.08%)	1.20
(0.97203) BD (1)Tm 2- N 31 (6.48%) 0.2546*Tm 2 s(5.76%)p 0.02(0.11%)d16.27(93.77%) f 0.05(0.26%)g 0.02(0.10%) (93.52%) 0.9670* N 31 s(40.81%)p 1.45(59.13%)d 0.00(0.06%)	(0.03231) LV (1) K 3 s(99.59%)p 0.00(0.34%)d 0.00(0.08%)	1.29
(0.97392) BD (1)Tm 2- N 35 (5.80%) 0.2408*Tm 2 s(5.83%)p 0.07(0.38%)d16.01(93.39%) f 0.05(0.29%)g 0.02(0.10%) (94.20%) 0.9706* N 35 s(42.54%)p 1.35(57.41%)d 0.00(0.06%)	(0.03231) LV (1) K 3 s(99.59%)p 0.00(0.34%)d 0.00(0.08%)	1.10
(0.97308) BD (1)Tm 1- N 31 (5.88%) 0.2426*Tm 1 s(4.80%)p 0.08(0.37%)d19.68(94.46%) f 0.06(0.28%)g 0.02(0.09%)	(0.03313) LV (1) K 4 s(99.46%)p 0.00(0.45%)d 0.00(0.09%)	1.42
(0.97192) BD (1)Tm 1- N 35 (6.57%) 0.2564*Tm 1 s(4.54%)p 0.03(0.12%)d20.94(94.96%) f 0.06(0.28%)g 0.02(0.10%) (93.43%) 0.9666* N 35 s(40.37%)p 1.48(59.57%)d 0.00(0.06%)	(0.03313) LV (1) K 4 s(99.46%)p 0.00(0.45%)d 0.00(0.09%)	1.32
(0.97203) BD (1)Tm 2- N 31 (6.48%) 0.2546*Tm 2 s(5.76%)p 0.02(0.11%)d16.27(93.77%) f 0.05(0.26%)g 0.02(0.10%) (93.52%) 0.9670* N 31 s(40.81%)p 1.45(59.13%)d 0.00(0.06%)	(0.03313) LV (1) K 4 s(99.46%)p 0.00(0.45%)d 0.00(0.09%)	1.43
(0.97308) BD (1)Tm 1- N 31	(0.03200) LV (1) K 5 s(99.55%)p 0.00(0.40%)d 0.00(0.05%)	1.15

(5.88%) 0.2426*Tm 1 s(4.80%)p 0.08(0.37%)d19.68(94.46%) f 0.06(0.28%)g 0.02(0.09%)		
(0.97192) BD (1)Tm 1- N 35 (6.57%) 0.2564*Tm 1 s(4.54%)p 0.03(0.12%)d20.94(94.96%) f 0.06(0.28%)g 0.02(0.10%) (93.43%) 0.9666* N 35 s(40.37%)p 1.48(59.57%)d 0.00(0.06%)	(0.03200) LV (1) K 5 s(99.55%)p 0.00(0.40%)d 0.00(0.05%)	1.22
(0.97203) BD (1)Tm 2- N 31 (6.48%) 0.2546*Tm 2 s(5.76%)p 0.02(0.11%)d16.27(93.77%) f 0.05(0.26%)g 0.02(0.10%) (93.52%) 0.9670* N 31 s(40.81%)p 1.45(59.13%)d 0.00(0.06%)	(0.03200) LV (1) K 5 s(99.55%)p 0.00(0.40%)d 0.00(0.05%)	1.23
(0.97392) BD (1)Tm 2- N 35 (5.80%) 0.2408*Tm 2 s(5.83%)p 0.07(0.38%)d16.01(93.39%) f 0.05(0.29%)g 0.02(0.10%) (94.20%) 0.9706* N 35 s(42.54%)p 1.35(57.41%)d 0.00(0.06%)	(0.03200) LV (1) K 5 s(99.55%)p 0.00(0.40%)d 0.00(0.05%)	1.14
(0.95660) LP (1) O 17 s(36.23%)p 1.76(63.76%)d 0.00(0.01%)	(0.07268) LV (3)Tm 2 s(0.15%)p 2.41(0.35%)d99.99(99.33%)f 0.82(0.12%)g 0.36(0.05%)	10.64
(0.95660) LP (1) O 17 s(36.23%)p 1.76(63.76%)d 0.00(0.01%)	(0.05151) BD*(1)Tm 2- N 35 (94.20%) 0.9706*Tm 2 s(5.83%)p 0.07(0.38%)d16.01(93.39%)f 0.05(0.29%)g 0.02(0.10%) (5.80%) -0.2408* N 35 s(42.54%)p 1.35(57.41%)d 0.00(0.06%)	4.19
(0.92911) LP (2) O 17 s(0.59%)p99.99(99.38%)d 0.05(0.03%)	(0.07777) LV (2)Tm 2 s(0.17%)p 0.63(0.10%)d99.99(99.65%)f 0.18(0.03%)g 0.27(0.04%)	4.97
(0.92360) LP (3) O 17 s(1.19%)p82.87(98.78%)d 0.03(0.03%)	(0.10414) LV (1)Tm 2 s(0.15%)p 3.22(0.49%)d99.99(99.31%)f 0.23(0.03%)g 0.10(0.02%)	6.86
(0.95660) LP (1) O 17 s(36.23%)p 1.76(63.76%)d 0.00(0.01%)	(0.03200) LV (1) K 5 s(99.55%)p 0.00(0.40%)d 0.00(0.05%)	1.01
(0.98005) BD (1)Si 6- O 17 (12.23%) 0.3497*Si 6 s(24.74%)p 3.00(74.28%)d 0.04(0.98%) (87.77%) 0.9369* O 17 s(61.93%)p 0.61(37.94%)d 0.00(0.13%)	(0.07268) LV (3)Tm 2 s(0.15%)p 2.41(0.35%)d99.99(99.33%)f 0.82(0.12%)g 0.36(0.05%)	5.70
(0.95796) LP (1) O 19 s(35.37%)p 1.83(64.62%)d 0.00(0.01%)	(0.10580) LV (1)Tm 1 s(0.02%)p29.83(0.49%)d99.99(99.45%) f 2.03(0.03%)g 0.89(0.01%)	16.53
(0.95796) LP (1) O 19 s(35.37%)p 1.83(64.62%)d 0.00(0.01%)	(0.04998) BD*(1)Tm 1- N 31 (94.12%) 0.9701*Tm 1 s(4.80%)p 0.08(0.37%)d19.68(94.46%)f 0.06(0.28%)g 0.02(0.09%) (5.88%) -0.2426* N 31 s(41.52%)p 1.41(58.42%)d 0.00(0.05%)	4.08
(0.93205) LP (2) O 19 s(0.49%)p99.99(99.48%)d 0.06(0.03%)	(0.07810) LV (2)Tm 1 s(0.22%)p 1.01(0.23%)d99.99(99.46%)f 0.20(0.05%)g 0.21(0.05%)	6.05
231. BD (1)Si 7- O 19	(0.10580) LV (1)Tm 1 s(0.02%)p29.83(0.49%)d99.99(99.45%) f 2.03(0.03%)g 0.89(0.01%)	9.82
(0.95796) LP (1) O 19 s(35.37%)p 1.83(64.62%)d 0.00(0.01%)	(0.03313) LV (1) K 4 s(99.46%)p 0.00(0.45%)d 0.00(0.09%)	1.27

(0.95813) LP (1) O 22<br>s(34.71%)p 1.88(65.28%)d 0.00(0.01%)	(0.07810) LV (2)Tm 1<br>s(0.22%)p 1.01(0.23%)d99.99(99.46%)f 0.20(0.05%)g 0.21(0.05%)	3.68
(0.95813) LP (1) O 22<br>s(34.71%)p 1.88(65.28%)d 0.00(0.01%)	(0.07286) LV (3)Tm 1<br>s(0.05%)p 5.06(0.27%)d99.99(99.53%)f 1.98(0.11%)g 0.76(0.04%)	7.70
(0.95813) LP (1) O 22<br>s(34.71%)p 1.88(65.28%)d 0.00(0.01%)	(0.04998) BD*(1)Tm 1- N 31<br>(94.12%) 0.9701*Tm 1 s(4.80%)p 0.08(0.37%)d19.68(94.46%)f 0.06(0.28%)g 0.02(0.09%)<br>(5.88%) -0.2426* N 31 s(41.52%)p 1.41(58.42%)d 0.00(0.05%)	4.36
(0.95813) LP (1) O 22<br>s(34.71%)p 1.88(65.28%)d 0.00(0.01%)	(0.04508) BD*(1)Tm 1- N 35<br>(93.43%) 0.9666*Tm 1 s(4.54%)p 0.03(0.12%)d20.94(94.96%)f 0.06(0.28%)g 0.02(0.10%)<br>(6.57%) -0.2564* N 35 s(40.37%)p 1.48(59.57%)d 0.00(0.06%)	3.88
(0.92879) LP (2) O 22<br>s(1.86%)p52.83(98.11%)d 0.02(0.03%)	(0.10580) LV (1)Tm 1<br>s(0.02%)p29.83(0.49%)d99.99(99.45%) f 2.03(0.03%)g 0.89(0.01%)	5.23
(0.92311) LP (3) O 22<br>s(2.01%)p48.65(97.95%)d 0.02(0.03%)	(0.07810) LV (2)Tm 1<br>s(0.22%)p 1.01(0.23%)d99.99(99.46%)f 0.20(0.05%)g 0.21(0.05%)	6.65
(0.97945) BD (1)Si 8- O 22<br>(12.05%) 0.3472*Si 8 s(25.06%)p 2.95(73.97%)d 0.04(0.96%)<br>(87.95%) 0.9378* O 22 s(61.35%)p 0.63(38.51%)d 0.00(0.14%)	(0.07286) LV (3)Tm 1<br>s(0.05%)p 5.06(0.27%)d99.99(99.53%)f 1.98(0.11%)g 0.76(0.04%)	4.35
(0.95813) LP (1) O 22<br>s(34.71%)p 1.88(65.28%)d 0.00(0.01%)	(0.03231) LV (1) K 3<br>s(99.59%)p 0.00(0.34%)d 0.00(0.08%)	1.23
(0.95950) LP (1) O 16<br>s(35.52%)p 1.82(64.47%)d 0.00(0.01%)	(0.07268) LV (3)Tm 2<br>s(0.15%)p 2.41(0.35%)d99.99(99.33%)f 0.82(0.12%)g 0.36(0.05%)	7.96
(0.95950) LP (1) O 16<br>s(35.52%)p 1.82(64.47%)d 0.00(0.01%)	(0.04445) BD*(1)Tm 2- N 31<br>(93.52%) 0.9670*Tm 2 s(5.76%)p 0.02(0.11%)d16.27(93.77%)f 0.05(0.26%)g 0.02(0.10%)(6.48%)<br>-0.2546* N 31 s(40.81%)p 1.45(59.13%)d 0.00(0.06%)	3.74
(0.95950) LP (1) O 16<br>s(35.52%)p 1.82(64.47%)d 0.00(0.01%)	(0.05151) BD*(1)Tm 2- N 35<br>(94.20%) 0.9706*Tm 2 s(5.83%)p 0.07(0.38%)d16.01(93.39%)f 0.05(0.29%)g 0.02(0.10%)<br>(5.80%) -0.2408* N 35 s(42.54%)p 1.35(57.41%)d 0.00(0.06%)	7.13
(0.92694) LP (2) O 16<br>s(0.82%)p99.99(99.15%)d 0.04(0.03%)	(0.10414) LV (1)Tm 2<br>s(0.15%)p 3.22(0.49%)d99.99(99.31%)f 0.23(0.03%)g 0.10(0.02%)	5.77
(0.92088) LP (3) O 16<br>s(3.61%)p26.68(96.35%)d 0.01(0.03%)	(0.07777) LV (2)Tm 2<br>s(0.17%)p 0.63(0.10%)d99.99(99.65%)f 0.18(0.03%)g 0.27(0.04%)	4.45
(0.97957) BD (1)Si 10- O 16<br>(12.06%) 0.3473*Si 10 s(25.24%)p 2.92(73.74%)d 0.04(1.02%)<br>(87.94%) 0.9377* O 16 s(59.98%)p 0.66(39.88%)d 0.00(0.14%)	(0.07268) LV (3)Tm 2<br>s(0.15%)p 2.41(0.35%)d99.99(99.33%)f 0.82(0.12%)g 0.36(0.05%)	4.53
(0.97957) BD (1)Si 10- O 16<br>(12.06%) 0.3473*Si 10 s(25.24%)p 2.92(73.74%)d 0.04(1.02%)	(0.05151) BD*(1)Tm 2- N 35	3.79

(87.94%) 0.9377* O 16 s(59.98%)p 0.66(39.88%)d 0.00(0.14%)	(94.20%) 0.9706*Tm 2 s(5.83%)p 0.07(0.38%)d16.01(93.39%)f 0.05(0.29%)g 0.02(0.10%)<br>(5.80%) -0.2408* N 35 s(42.54%)p 1.35(57.41%)d 0.00(0.06%)	
(0.95827) LP (1) O 24<br>s(36.29%)p 1.76(63.70%)d 0.00(0.01%)	(0.10414) LV (1)Tm 2<br>s(0.15%)p 3.22(0.49%)d99.99(99.31%)f 0.23(0.03%)g 0.10(0.02%)	16.95
(0.95827) LP (1) O 24<br>s(36.29%)p 1.76(63.70%)d 0.00(0.01%)	(0.05151) BD*(1)Tm 2- N 35<br>(94.20%) 0.9706*Tm 2 s(5.83%)p 0.07(0.38%)d16.01(93.39%)f 0.05(0.29%)g 0.02(0.10%)<br>(5.80%) -0.2408* N 35 s(42.54%)p 1.35(57.41%)d 0.00(0.06%)	3.96
(0.93149) LP (2) O 24<br>s(0.03%)p99.99(99.94%)d 1.25(0.03%)	(0.07777) LV (2)Tm 2<br>s(0.17%)p 0.63(0.10%)d99.99(99.65%)f 0.18(0.03%)g 0.27(0.04%)	7.01
(0.92422) LP (3) O 24<br>s(0.11%)p99.99(99.85%)d 0.31(0.03%)	(0.07268) LV (3)Tm 2<br>s(0.15%)p 2.41(0.35%)d99.99(99.33%)f 0.82(0.12%)g 0.36(0.05%)	4.66
(0.97984) BD (1)Si 11- O 24<br>(12.28%) 0.3504*Si 11 s(24.87%)p 2.98(74.20%)d 0.04(0.93%)<br>(87.72%) 0.9366* O 24 s(63.51%)p 0.57(36.35%)d 0.00(0.14%)	(0.10414) LV (1)Tm 2<br>s(0.15%)p 3.22(0.49%)d99.99(99.31%)f 0.23(0.03%)g 0.10(0.02%)	10.05
(0.95827) LP (1) O 24<br>s(36.29%)p 1.76(63.70%)d 0.00(0.01%)	(0.03231) LV (1) K 3<br>s(99.59%)p 0.00(0.34%)d 0.00(0.08%)	1.08
(0.95683) LP (1) O 12<br>s(36.10%)p 1.77(63.89%)d 0.00(0.01%)	(0.07286) LV (3)Tm 1<br>s(0.05%)p 5.06(0.27%)d99.99(99.53%)f 1.98(0.11%)g 0.76(0.04%)	8.85
(0.95683) LP (1) O 12<br>s(36.10%)p 1.77(63.89%)d 0.00(0.01%)	(0.04998) BD*(1)Tm 1- N 31<br>(94.12%) 0.9701*Tm 1 s(4.80%)p 0.08(0.37%)d19.68(94.46%)f 0.06(0.28%)g 0.02(0.09%)<br>(5.88%) -0.2426* N 31 s(41.52%)p 1.41(58.42%)d 0.00(0.05%)	4.39
(0.92922) LP (2) O 12<br>s(0.54%)p99.99(99.43%)d 0.05(0.03%)	(0.07810) LV (2)Tm 1<br>s(0.22%)p 1.01(0.23%)d99.99(99.46%)f 0.20(0.05%)g 0.21(0.05%)	4.30
(0.92408) LP (3) O 12<br>s(0.81%)p99.99(99.15%)d 0.04(0.03%)	(0.10580) LV (1)Tm 1<br>s(0.02%)p29.83(0.49%)d99.99(99.45%) f 2.03(0.03%)g 0.89(0.01%)	5.51
238. BD (1)Si 9- O 12	(0.07286) LV (3)Tm 1<br>s(0.05%)p 5.06(0.27%)d99.99(99.53%)f 1.98(0.11%)g 0.76(0.04%)	4.66
(0.95683) LP (1) O 12<br>s(36.10%)p 1.77(63.89%)d 0.00(0.01%)	(0.03200) LV (1) K 5<br>s(99.55%)p 0.00(0.40%)d 0.00(0.05%)	1.04

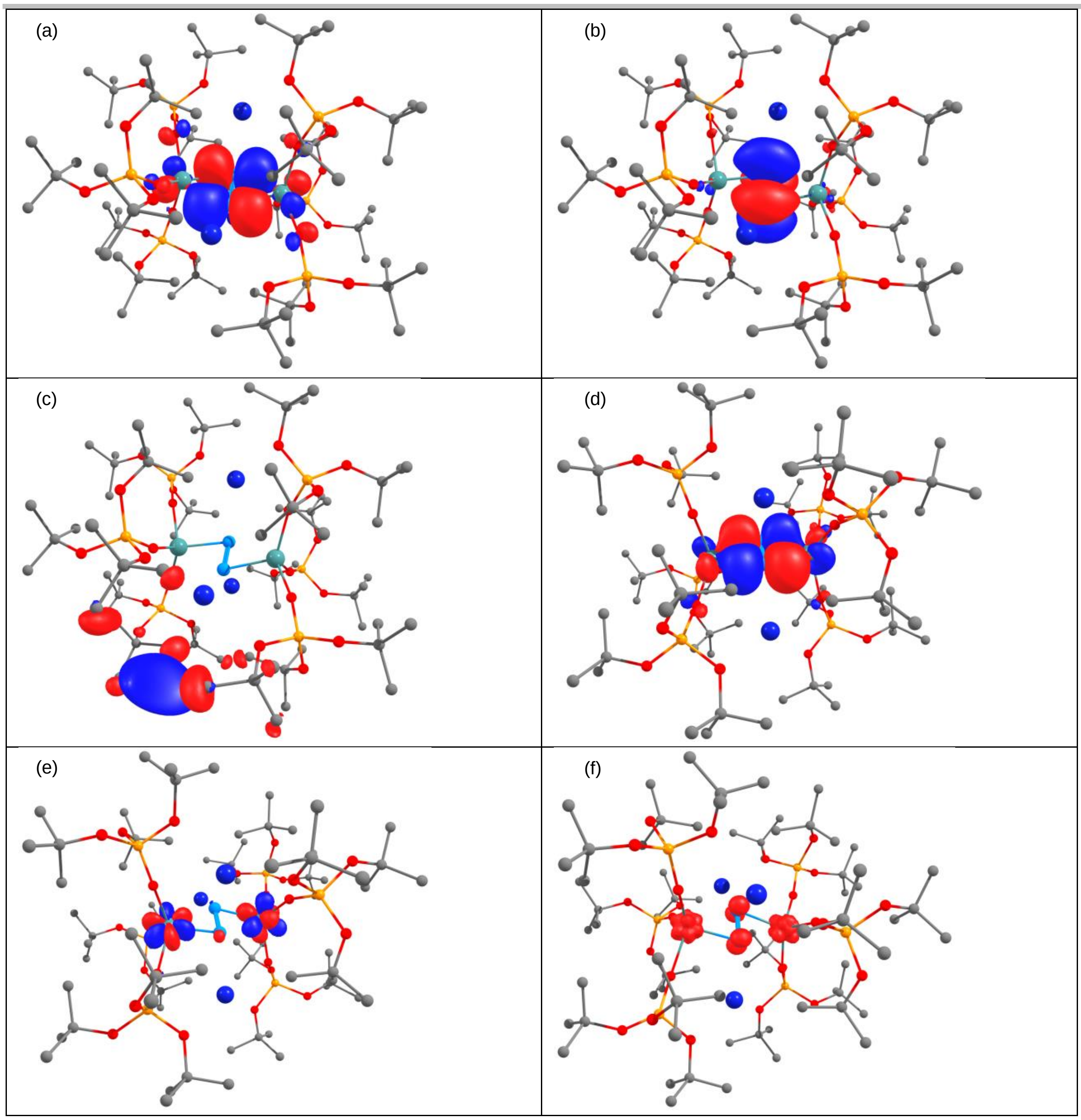

Figure S70. Computed MOs for complex **2**. (a)AMO-HOMO-1 (b)AMO-HOMO (c)AMO-LUMO (d)BMO-HOMO (e)BMO-LUMO (f) spin density plot (spin densities on thulium atoms: 1.92, 1.95 and nitrogen atoms: 0.52, 0.53).

Optimized coordinates

Complex **1**

69	-0.333107967	0.564443854	13.001541589
19	-0.679294000	0.583934000	9.544174000
14	0.192813033	3.652797854	11.020894589
14	-3.669158967	-0.124483146	11.718715589
14	1.714686033	-1.542442146	10.791237589
8	-2.327583967	0.047717854	12.596382589
8	-0.224883967	2.338840854	11.856344589
8	0.730366033	-0.553233146	11.600527589
8	-3.199372967	-0.210356146	10.092936589
8	-0.078640967	3.209030854	9.412918589
8	1.357801033	-1.176314146	9.184312589
8	-4.777530967	1.104177854	11.927590589

8	-4.530156967	-1.520133146	12.003576589
8	-0.736308967	5.012349854	11.255422589
8	1.765453033	4.149491854	11.263459589
8	3.350240033	-1.289304146	10.965024589
8	1.467127033	-3.151264146	11.134700589
6	-4.508010967	2.508740854	12.001767589
6	-3.496228967	2.930931854	10.940318589
1	-2.521954967	2.476952854	11.135017589
1	-3.344339967	4.012714854	10.949095589
1	-3.841694967	2.634060854	9.946039589
6	-4.029005967	-0.635007146	8.994197589
6	0.228696033	-3.784042146	11.494936589
6	-0.107871967	4.058800854	8.256134589
6	2.087195033	-1.559853146	8.008409589
6	4.129145033	-1.554480146	12.145304589
6	-0.949382967	-3.176773146	10.735564589
1	-1.855746967	-3.765651146	10.899235589
1	-0.735715967	-3.162900146	9.662195589
1	-1.157296967	-2.158524146	11.070069589
6	0.039875033	-3.644964146	13.002244589
1	-0.027302967	-2.592831146	13.290494589
1	0.888925033	-4.087910146	13.528025589
1	-0.872538967	-4.150332146	13.330426589
6	-4.763464967	-2.098228146	13.297971589
6	3.030769033	2.904019854	12.882497589
1	2.163143033	2.315427854	13.189232589
1	3.070529033	3.792527854	13.518396589
1	3.933527033	2.312661854	13.064639589
6	-0.711356967	5.865040854	12.411373589
6	2.923452033	3.314105854	11.416281589
6	-5.851366967	3.191865854	11.761739589
1	-6.230580967	2.939551854	10.767085589
1	-5.753786967	4.279632854	11.832809589
1	-6.583265967	2.854890854	12.501418589
6	-5.466420967	-0.147738146	9.159937589
1	-5.914719967	-0.570410146	10.061871589
1	-6.064641967	-0.457233146	8.297134589
1	-5.501918967	0.941118854	9.239856589
6	-3.985480967	2.834455854	13.400378589
1	-4.689259967	2.475046854	14.156983589
1	-3.850247967	3.912181854	13.531314589
1	-3.024520967	2.341818854	13.560651589
6	2.839900033	2.091843854	10.506909589
1	3.761383033	1.507307854	10.547619589
1	2.671407033	2.400896854	9.471556589
1	2.028881033	1.429305854	10.816219589
6	0.403625033	-5.250651146	11.110265589
1	-0.473610967	-5.836975146	11.400420589
1	1.283382033	-5.667211146	11.609043589
1	0.545889033	-5.345611146	10.029604589
6	-3.979897967	-2.158478146	8.913608589
1	-2.946738967	-2.495382146	8.790340589
1	-4.568761967	-2.522258146	8.065193589
1	-4.375703967	-2.586813146	9.836358589
6	-3.412790967	-0.014775146	7.739522589
1	-3.350280967	1.074146854	7.841009589
1	-4.019083967	-0.239198146	6.857413589
1	-2.411911967	-0.421607146	7.550622589
6	4.109542033	4.189412854	11.021207589
1	4.137287033	5.089668854	11.641964589
1	4.023297033	4.497555854	9.975095589

1	5.051552033	3.646967854	11.148737589
6	-1.493152967	4.693239854	8.160044589
1	-1.675128967	5.314822854	9.038837589
1	-2.261039967	3.913842854	8.126257589
1	-1.580583967	5.307370854	7.257881589
6	0.978521033	5.127825854	8.335305589
1	0.966570033	5.745287854	7.431731589
1	1.965285033	4.668690854	8.433575589
1	0.817123033	5.774955854	9.200342589
6	-4.973651967	-1.015999146	14.356485589
1	-5.789269967	-0.348975146	14.066669589
1	-4.064025967	-0.420719146	14.476393589
1	-5.212336967	-1.473427146	15.321654589
6	0.146269033	3.133612854	7.066624589
1	-0.650482967	2.384939854	6.981864589
1	1.104589033	2.617582854	7.184650589
1	0.169636033	3.696003854	6.128684589
6	1.087774033	-1.448243146	6.859002589
1	0.727451033	-0.417459146	6.763405589
1	0.230710033	-2.105723146	7.036663589
1	1.549066033	-1.729259146	5.907721589
6	4.484964033	-3.038105146	12.184850589
1	5.097438033	-3.262835146	13.063889589
1	5.048588033	-3.313142146	11.288148589
1	3.574390033	-3.639568146	12.217325589
6	-6.020716967	-2.947529146	13.143667589
1	-6.258707967	-3.466372146	14.077459589
1	-5.875908967	-3.694417146	12.357430589
1	-6.869901967	-2.316233146	12.866680589
6	3.241493033	-0.578711146	7.819627589
1	3.797646033	-0.800549146	6.903074589
1	3.918669033	-0.635828146	8.674244589
1	2.857010033	0.443898854	7.754531589
6	3.353066033	-1.147378146	13.394711589
1	2.446069033	-1.747299146	13.512486589
1	3.067767033	-0.094887146	13.331810589
1	3.978441033	-1.289696146	14.281126589
6	-3.556863967	-2.962439146	13.653518589
1	-2.660363967	-2.341174146	13.709836589
1	-3.402106967	-3.724286146	12.884018589
1	-3.698852967	-3.463147146	14.616386589
6	5.385902033	-0.700176146	12.014307589
1	6.060651033	-0.876102146	12.857435589
1	5.125005033	0.361585854	11.998028589
1	5.913027033	-0.942108146	11.086945589
6	2.600676033	-2.991672146	8.133076589
1	1.773540033	-3.683071146	8.312563589
1	3.301551033	-3.075264146	8.967442589
1	3.117040033	-3.290373146	7.215520589
6	-2.071316967	6.556224854	12.435846589
1	-2.134329967	7.264512854	13.267620589
1	-2.870165967	5.817628854	12.546462589
1	-2.233630967	7.101044854	11.501182589
6	0.411293033	6.885844854	12.237681589
1	0.252668033	7.463910854	11.321985589
1	1.371894033	6.372136854	12.161397589
1	0.440210033	7.580058854	13.083866589
6	-0.507877967	5.046905854	13.685397589
1	-0.546629967	5.699516854	14.562692589
1	0.464295033	4.545907854	13.679460589
1	-1.286985967	4.286313854	13.780634589

7	0.588459033	-0.356270146	14.870986589
7	0.935046967	0.783767146	14.863592411
69	1.931366033	-0.108232146	16.696120589
19	2.301643000	-0.460647000	20.127222000
14	1.834084033	-3.404812146	18.389641589
14	5.153542033	0.925751854	18.063604589
14	-0.335727967	1.537674854	19.070501589
8	3.874206033	0.558202854	17.153422589
8	2.049227033	-1.964612146	17.698347589
8	0.761692033	0.765463854	18.174955589
8	4.648529033	0.881795854	19.680703589
8	2.072405033	-3.100075146	20.034411589
8	0.121909033	1.121506854	20.639263589
8	6.457227033	-0.095399146	17.856944589
8	5.777405033	2.450291854	17.829419589
8	2.939009033	-4.587447146	18.002874589
8	0.339828033	-4.082927146	18.100942589
8	-1.917765967	1.048777854	18.913245589
8	-0.344298967	3.184162854	18.834585589
6	6.419222033	-1.517188146	17.696572589
6	5.385871033	-2.151354146	18.623879589
1	4.375003033	-1.845002146	18.343858589
1	5.413912033	-3.241626146	18.559024589
1	5.578883033	-1.859801146	19.660528589
6	5.386882033	1.381588854	20.812604589
6	0.761605033	4.012287854	18.441385589
6	2.238721033	-4.055446146	21.093134589
6	-0.620544967	1.311525854	21.853619589
6	-2.785350967	1.301026854	17.792820589
6	2.045832033	3.583557854	19.146853589
1	2.849532033	4.299301854	18.955139589
1	1.877224033	3.534836854	20.227024589
1	2.386752033	2.606026854	18.800171589
6	0.906837033	3.934306854	16.924418589
1	1.157580033	2.919719854	16.606169589
1	-0.035668967	4.215043854	16.446905589
1	1.690171033	4.608976854	16.567324589
6	5.926406033	3.093538854	16.552882589
6	-1.142297967	-2.882840146	16.640237589
1	-0.365778967	-2.166841146	16.361917589
1	-1.107090967	-3.710727146	15.926442589
1	-2.114743967	-2.389809146	16.545351589
6	3.084113033	-5.220266146	16.721000589
6	-0.924276967	-3.401599146	18.058842589
6	7.821750033	-2.007800146	18.043452589
1	8.056075033	-1.779535146	19.087430589
1	7.903242033	-3.089365146	17.896666589
1	8.561884033	-1.509667146	17.410820589
6	6.881324033	1.104504854	20.669955589
1	7.287190033	1.616399854	19.794501589
1	7.410946033	1.462843854	21.558341589
1	7.069863033	0.034667854	20.555456589
6	6.087013033	-1.821385146	16.237414589
1	6.821063033	-1.350801146	15.576930589
1	6.078495033	-2.898430146	16.044654589
1	5.102276033	-1.411623146	16.004730589
6	-0.970300967	-2.261507146	19.072745589
1	-1.965017967	-1.812921146	19.113626589
1	-0.715668967	-2.630552146	20.070246589
1	-0.274022967	-1.466196146	18.798745589
6	0.372076033	5.424860854	18.868689589

1	1.138432033	6.147086854	18.571042589
1	-0.576716967	5.710177854	18.405179589
1	0.249914033	5.472074854	19.954891589
6	5.125119033	2.879789854	20.939306589
1	4.054137033	3.065795854	21.055314589
1	5.647225033	3.292660854	21.808703589
1	5.469366033	3.389587854	20.037503589
6	4.835407033	0.640648854	22.031162589
1	4.942675033	-0.442343146	21.903141589
1	5.375107033	0.928277854	22.937858589
1	3.779521033	0.887203854	22.194498589
6	-1.967383967	-4.460408146	18.405230589
1	-1.894398967	-5.302414146	17.710933589
1	-1.805563967	-4.837997146	19.419156589
1	-2.977666967	-4.043526146	18.345737589
6	3.702010033	-4.489687146	21.123006589
1	3.960062033	-4.978405146	20.181456589
1	4.350409033	-3.616444146	21.245507589
1	3.888269033	-5.180562146	21.951661589
6	1.317806033	-5.256380146	20.895740589
1	1.432756033	-5.960209146	21.725962589
1	0.273513033	-4.938341146	20.847378589
1	1.558918033	-5.774744146	19.964742589
6	6.272899033	2.085013854	15.456838589
1	7.176095033	1.529046854	15.720345589
1	5.454861033	1.372019854	15.319260589
1	6.439781033	2.603447854	14.507274589
6	1.869792033	-3.309018146	22.374331589
1	2.547142033	-2.461978146	22.536007589
1	0.843705033	-2.932484146	22.312734589
1	1.946348033	-3.963695146	23.247346589
6	0.420336033	1.293469854	22.971041589
1	0.941870033	0.329572854	22.994907589
1	1.157386033	2.087331854	22.814477589
1	-0.048615967	1.441882854	23.948180589
6	-3.346211967	2.716621854	17.903140589
1	-4.032742967	2.922635854	17.075603589
1	-3.892995967	2.831781854	18.844066589
1	-2.533554967	3.445139854	17.885857589
6	7.061421033	4.096327854	16.732950589
1	7.223780033	4.671716854	15.816209589
1	6.823892033	4.791780854	17.543453589
1	7.987821033	3.573885854	16.988193589
6	-1.597625967	0.147918854	22.002841589
1	-2.154453967	0.221877854	22.942556589
1	-2.300353967	0.146726854	21.167145589
1	-1.053930967	-0.802145146	21.995286589
6	-2.029447967	1.116372854	16.479074589
1	-1.223023967	1.849952854	16.383707589
1	-1.592916967	0.116131854	16.429303589
1	-2.710657967	1.240884854	15.631713589
6	4.614667033	3.803789854	16.229934589
1	3.806989033	3.073257854	16.144114589
1	4.362798033	4.506754854	17.029010589
1	4.689336033	4.357234854	15.288792589
6	-3.905050967	0.271016854	17.901837589
1	-4.652248967	0.429431854	17.119007589
1	-3.504996967	-0.741164146	17.796292589
1	-4.395832967	0.350506854	18.876157589
6	-1.358865967	2.647161854	21.834184589
1	-0.658699967	3.471499854	21.677456589

1	-2.093625967	2.665863854	21.025453589
1	-1.882901967	2.804388854	22.782031589
6	4.500097033	-5.787989146	16.707912589
1	4.697766033	-6.321821146	15.773353589
1	5.232184033	-4.981992146	16.810375589
1	4.634897033	-6.482987146	17.541880589
6	2.051041033	-6.338674146	16.603186589
1	2.185974033	-7.060347146	17.414644589
1	1.042939033	-5.925390146	16.675299589
1	2.157449033	-6.865497146	15.649262589
6	2.911609033	-4.201425146	15.596417589
1	3.087524033	-4.671194146	14.624167589
1	1.900471033	-3.784437146	15.597351589
1	3.619820033	-3.379069146	15.716884589

Complex **2**

69	35.583312658	3.708618756	17.697340969
69	32.251712342	2.940931244	20.113278031
19	34.276691000	5.998920000	20.499816000
19	32.294217000	3.184256000	16.189726000
19	35.192927000	0.834946000	19.864972000
14	33.364317342	0.812264244	22.893923031
14	34.327331658	5.673840756	14.826078969
14	37.760708658	5.999372756	19.631958969
14	37.181262658	0.484820756	16.945421969
14	29.640759342	1.327934244	18.044184031
14	30.794754342	6.200509244	20.923484031
8	36.665316658	1.887908756	17.541219969
8	36.413709658	-0.684892244	17.892929969
8	34.955739342	0.334742244	22.582734031
8	32.706688658	5.348711756	14.469686969
8	30.616601342	2.173378244	18.993199031
8	32.866964342	1.467398244	21.509761031
8	36.806227658	6.763467756	20.804571969
8	34.685347658	4.611810756	15.981942969
8	35.143398658	5.484125756	13.385414969
8	32.569445342	-0.571496756	23.367115031
8	36.725680658	5.215688756	18.687106969
8	30.012769342	1.785346244	16.454398031
8	31.750751342	4.918615244	20.742804031
8	31.862415342	7.505551244	20.784899031
8	34.557647658	7.274014756	15.242007969
8	33.279712342	1.821773244	24.220717031
8	38.810924658	0.185800756	17.101842969
8	36.863594658	0.254740756	15.321668969
8	29.521020342	6.286581244	19.848948031
7	33.736598342	2.851769244	18.490529031
8	30.066185342	6.345025244	22.416186031
8	38.923791658	5.037777756	20.351081969
8	38.612762658	7.229230756	18.902453969
7	34.253911658	3.463591756	19.414694969
6	36.733841658	-2.084022244	17.983232969
6	36.495049658	5.895487756	13.119912969
6	31.723962342	8.822712244	21.345718031
8	28.016831342	1.655098244	18.220170031
6	35.765314342	-0.560242756	23.365058031
6	30.992497342	-1.072576756	18.408620031
6	31.269188342	-0.627828756	23.980460031
6	31.970225658	5.804626756	13.320919969
6	35.452357658	-2.773038244	18.446670969
1	35.617175658	-3.844265244	18.595854969

1	35.101798658	-2.354015244	19.395982969
1	34.661601658	-2.645838244	17.701694969
6	37.165865658	-2.641177244	16.629583969
1	37.356487658	-3.715888244	16.709863969
1	36.385994658	-2.479270244	15.881649969
1	38.078200658	-2.150382244	16.284739969
6	29.467491342	6.235273244	18.422312031
6	36.493421658	7.374327756	12.736332969
1	37.506177658	7.712134756	12.493535969
1	35.859107658	7.532446756	11.858532969
1	36.102936658	7.973857756	13.560809969
6	32.221914658	4.822273756	12.178523969
1	31.651623658	5.102386756	11.286984969
1	33.286495658	4.809259756	11.934921969
1	31.920118658	3.811660756	12.473123969
6	35.444162342	-1.989433756	22.932409031
1	36.051680342	-2.712853756	23.485658031
1	34.386628342	-2.197432756	23.108994031
1	35.652021342	-2.118397756	21.864961031
6	38.759196658	3.723367756	20.897342969
6	39.732520658	7.071354756	18.016378969
6	34.294387658	7.931333756	16.488838969
6	37.218559658	7.799387756	21.711997969
6	32.718369342	9.696980244	20.585956031
1	32.716116342	10.719778244	20.974252031
1	33.735359342	9.301915244	20.684552031
1	32.463610342	9.730480244	19.522594031
6	27.172762342	1.198841244	19.290878031
6	32.085226342	8.756434244	22.828984031
1	32.041305342	9.749971244	23.286552031
1	31.393452342	8.090389244	23.348606031
1	33.101827342	8.369853244	22.955823031
6	36.960862658	5.039599756	11.946469969
1	37.978718658	5.309832756	11.648809969
1	36.944357658	3.981129756	12.216627969
1	36.297290658	5.186221756	11.089170969
6	29.216118342	1.525183244	15.285785031
6	37.845796658	-2.248758244	19.017206969
1	38.115100658	-3.302760244	19.139663969
1	38.727125658	-1.686365244	18.701111969
1	37.518668658	-1.867718244	19.990355969
6	28.583367342	0.136998244	15.340143031
1	28.018907342	-0.052598756	14.421697031
1	29.351362342	-0.632791756	15.446764031
1	27.901775342	0.057639244	16.188836031
6	37.381925658	5.658733756	14.341709969
1	38.416872658	5.938538756	14.123771969
1	37.040358658	6.262522756	15.187508969
1	37.365883658	4.604814756	14.632983969
6	28.833768342	4.239239244	22.310475031
1	27.939081342	3.720668244	22.667902031
1	28.811233342	4.240056244	21.216833031
1	29.709802342	3.677227244	22.644806031
6	38.638534658	7.561227756	22.218256969
1	38.914729658	8.338467756	22.937728969
1	38.713800658	6.587304756	22.707384969
1	39.351365658	7.583402756	21.391697969
6	28.871010342	5.670130244	22.847051031
8	29.782645342	-0.330752756	18.208846031
6	30.304322342	9.352302244	21.162364031
1	30.222135342	10.361843244	21.577141031

1	30.041129342	9.390779244	20.103482031
1	29.589497342	8.706829244	21.676557031
6	37.117458658	9.139849756	20.987471969
1	37.384474658	9.966523756	21.653860969
1	37.784436658	9.141610756	20.123138969
1	36.093576658	9.301074756	20.634369969
6	35.509165342	-0.375032756	24.858232031
1	36.171134342	-1.027377756	25.436257031
1	35.694067342	0.660746244	25.152868031
1	34.474676342	-0.621927756	25.105055031
6	30.730607342	-2.020035756	23.666964031
1	29.749451342	-2.170137756	24.127969031
1	30.635370342	-2.156314756	22.587272031
1	31.415569342	-2.781878756	24.050860031
6	35.686724658	0.574479756	14.575144969
6	32.384671658	7.217472756	12.918256969
1	31.779581658	7.557390756	12.071977969
1	32.243693658	7.911292756	13.749986969
1	33.436237658	7.243053756	12.625907969
6	30.503081658	5.793592756	13.740113969
1	29.855675658	6.092377756	12.910313969
1	30.197920658	4.792608756	14.060475969
1	30.342431658	6.485353756	14.572304969
6	39.854306658	0.755429756	16.291620969
6	28.145805342	2.609952244	15.187467031
1	27.555045342	2.497692244	14.272427031
1	27.485038342	2.552599244	16.054444031
1	28.614448342	3.598896244	15.179377031
6	37.213538342	-0.204954756	23.036575031
1	37.906117342	-0.881496756	23.546099031
1	37.402340342	-0.282178756	21.960397031
1	37.435718342	0.818339244	23.352649031
6	39.523593658	2.199599756	15.914084969
1	40.351058658	2.645343756	15.353510969
1	38.629031658	2.238608756	15.286005969
1	39.346977658	2.800457756	16.808379969
6	30.178161342	1.604985244	14.101095031
1	29.661644342	1.389396244	13.161401031
1	30.607501342	2.608463244	14.009561031
1	30.989556342	0.878832244	14.220127031
6	35.802818658	2.003621756	14.056517969
1	34.980659658	2.251550756	13.377697969
1	35.779473658	2.734562756	14.866467969
1	36.742814658	2.119763756	13.510264969
6	30.348390342	0.446315244	23.401543031
1	29.342606342	0.353948244	23.822754031
1	30.724715342	1.445006244	23.640256031
1	30.279676342	0.350939244	22.316219031
6	27.970432342	0.988715244	20.576774031
1	27.300168342	0.725002244	21.400802031
1	28.692996342	0.179211244	20.450800031
1	28.511757342	1.898117244	20.844396031
6	39.669665658	5.737663756	17.273067969
1	40.479464658	5.671292756	16.539983969
1	39.771303658	4.904780756	17.972519969
1	38.716981658	5.637958756	16.749315969
6	41.005307658	7.156650756	18.855473969
1	41.897681658	7.071131756	18.226756969
1	41.044505658	8.114785756	19.383044969
1	41.011431658	6.350041756	19.593010969
6	39.056683658	2.697444756	19.809146969

1	39.030532658	1.678866756	20.211075969
1	38.330217658	2.748955756	18.995940969
1	40.055136658	2.873636756	19.398762969
6	31.434287342	-0.443373756	25.487861031
1	30.466384342	-0.513414756	25.994933031
1	32.090306342	-1.221093756	25.891344031
1	31.879022342	0.531474244	25.697616031
6	41.112652658	0.702767756	17.152097969
1	41.976992658	1.086439756	16.601268969
1	40.979205658	1.300839756	18.056581969
1	41.319950658	-0.329405244	17.449677969
6	33.778461658	9.323209756	16.133402969
1	34.501461658	9.840822756	15.496896969
1	32.829710658	9.256009756	15.592991969
1	33.620967658	9.919357756	17.038025969
6	35.662570658	-0.409094244	13.407171969
1	34.803428658	-0.220967244	12.755304969
1	36.576755658	-0.311307244	12.814950969
1	35.602754658	-1.436765244	13.776747969
6	39.785118658	3.613833756	22.022917969
1	39.766141658	2.615144756	22.471048969
1	40.790109658	3.803408756	21.635215969
1	39.575175658	4.350191756	22.804190969
6	37.352920658	3.529086756	21.451537969
1	37.259122658	2.542440756	21.913377969
1	37.140066658	4.283798756	22.212484969
1	36.592701658	3.611378756	20.667610969
6	36.233173658	7.739609756	22.877280969
1	36.473667658	8.490909756	23.635129969
1	35.212373658	7.938173756	22.532675969
1	36.260639658	6.751822756	23.348345969
6	34.435267658	0.400698756	15.429464969
1	33.539531658	0.603516756	14.831283969
1	34.366516658	-0.624460244	15.798367969
1	34.444879658	1.066355756	16.300196969
6	26.522083342	-0.108741756	18.845435031
1	25.844106342	-0.492263756	19.614938031
1	25.947914342	0.049512244	17.927140031
1	27.296579342	-0.855126756	18.650885031
6	31.289488342	-1.146625756	19.902923031
1	31.494546342	-0.159045756	20.320242031
1	30.432498342	-1.577743756	20.428118031
1	32.161456342	-1.779433756	20.099934031
6	28.927784342	5.671831244	24.371447031
1	28.034273342	5.201054244	24.792963031
1	29.808147342	5.128917244	24.721997031
1	28.986684342	6.699358244	24.742574031
6	39.642900658	8.229956756	17.028094969
1	40.501682658	8.231351756	16.349441969
1	38.728794658	8.147564756	16.433785969
1	39.620780658	9.182842756	17.564937969
6	28.081798342	5.700716244	18.069963031
1	27.935596342	5.694457244	16.985090031
1	27.970329342	4.675513244	18.432473031
1	27.306229342	6.325531244	18.522377031
6	40.021776658	-0.105285244	15.040838969
1	40.832475658	0.277638756	14.412368969
1	40.263395658	-1.134661244	15.323593969
1	39.093971658	-0.110547244	14.465228969
6	27.654848342	6.457385244	22.361455031
1	26.728704342	5.988120244	22.709011031

1	27.688981342	7.478934244	22.752986031
1	27.647346342	6.500338244	21.271160031
6	33.236677658	7.175259756	17.282596969
1	32.979188658	7.696093756	18.209150969
1	32.327753658	7.073460756	16.686679969
1	33.585239658	6.166098756	17.522196969
6	29.655714342	7.654347244	17.891482031
1	30.628601342	8.045781244	18.196937031
1	29.598683342	7.675887244	16.798256031
1	28.876468342	8.309416244	18.291265031
6	35.601610658	8.024296756	17.265145969
1	35.458719658	8.556660756	18.211696969
1	35.998900658	7.032797756	17.492948969
1	36.349173658	8.571491756	16.684601969
6	32.162166342	-0.437941756	17.662974031
1	32.391177342	0.561465244	18.047782031
1	33.059193342	-1.052997756	17.774761031
1	31.933465342	-0.356143756	16.597046031
6	30.712289342	-2.466275756	17.851889031
1	29.845249342	-2.903878756	18.354829031
1	30.495164342	-2.412212756	16.780899031
1	31.572795342	-3.126373756	18.001165031
6	26.129673342	2.294228244	19.489498031
1	25.391155342	1.996938244	20.240689031
1	26.609171342	3.219422244	19.821648031
1	25.608605342	2.494343244	18.548659031
6	33.588685342	3.214739244	24.326010031
6	34.002749342	3.432666244	25.779668031
1	33.194323342	3.128518244	26.450592031
1	34.887215342	2.834413244	26.016604031
1	34.232914342	4.486445244	25.966508031
6	34.733147342	3.592044244	23.393204031
1	35.622026342	3.004364244	23.629998031
1	34.477791342	3.405408244	22.344173031
1	34.984314342	4.651893244	23.511149031
6	32.335963342	4.022506244	24.008816031
1	32.048341342	3.931645244	22.960087031
1	31.508078342	3.673680244	24.632053031
1	32.488551342	5.088414244	24.204973031
6	30.522422342	5.292547244	17.854935031
1	31.522679342	5.581141244	18.183471031
1	30.322393342	4.268450244	18.180788031
1	30.491640342	5.336277244	16.761676031

J. References

[1] G. A. Bain, J. F. Berry, *J. Chem. Educ.* **2008**, *85*, 532.
[2] F. T. Edelmann, W. A. Herrmann, *Synthetic Methods of Organometallic and Inorganic Chemistry. 6. Lanthanides and Actinides: 28 Tables*, Thieme, **1997**.
[3] J. Andrez, J. Pécaut, P.-A. Bayle, M. Mazzanti, *Angew. Chem. Int. Ed. Engl.* ***2014**, 53, 10448-10452.*
[4] D. E. Bergbreiter, J. M. Killough, *J. Am. Chem. Soc.* **1978**, *100*, 2126–2134.
[5] J. Friedrich, C. Maichle-Mössmer, R. Anwander, *Chem. Commun.* **2017**, *53*, 12044–12047.
[6] G. Lapadula, M. P. Conley, C. Coperet, R. A. Andersen, *Organometallics* **2015**, *34*, 2271–2277.
[7] M. Nishiura, Z. Hou, Y. Wakatsuki, *Organometallics* **2004**, *23*, 1359–1368.
[8] A. B. Chung, D. Rappoport, J. W. Ziller, R. E. Cramer, F. Furche, W. J. Evans, *J. Am. Chem. Soc.* **2022**, *144*, 17064–17074.
[9] J. Y. W. Mak, *Aust. J. Chem.* **2021**, *75*, 160–164.
[10] G. Wider, L. Dreier, *J. Am. Chem. Soc.* **2006**, *128*, 2571–2576.
[11] **CrysAlisPro** (Rigaku, V1.171.43.107a, 2024).
[12] G. M. Sheldrick, *Acta Cryst A* **2015**, *71*, 3–8.
[13] G. M. Sheldrick, *Acta Cryst C* **2015**, *71*, 3–8.
[14] O. V. Dolomanov, L. J. Bourhis, R. J. Gildea, J. a. K. Howard, H. Puschmann, *J Appl Cryst* **2009**, *42*, 339–341.
[15] S. Cotton, *Lanthanide and Actinide Chemistry*, John Wiley & Sons, **2013**.
[16] H. M. Nicholas, M. Vonci, C. A. P. Goodwin, S. W. Loo, S. R. Murphy, D. Cassim, R. E. P. Winpenny, E. J. L. McInnes, N. F. Chilton, D. P. Mills, *Chem. Sci.* **2019**, *10*, 10493–10502.
[17] A. D. Becke, *J. Chem. Phys.* **1993**, *98*, 5648–5652.
[18] K. Burke, J. P. Perdew, Y. Wang, in *Electronic Density Functional Theory: Recent Progress and New Directions* (Eds.: J.F. Dobson, G. Vignale, M.P. Das), Springer US, Boston, MA, **1998**, pp. 81–111.
[19] M. Dolg, H. Stoll, A. Savin, H. Preuss, *Theoret. Chim. Acta* **1989**, *75*, 173–194.
[20] A. Höllwarth, M. Böhme, S. Dapprich, A. W. Ehlers, A. Gobbi, V. Jonas, K. F. Köhler, R. Stegmann, A. Veldkamp, G. Frenking, *Chem.Phys. Lett.* **1993**, *208*, 237–240.
[21] M. Dolg, H. Stoll, H. Preuss, *Theoret. Chim. Acta* **1993**, *85*, 441–450.
[22] A. Bergner, M. Dolg, W. Küchle, H. Stoll, H. Preuß, *Mol. Phys.***1993**, *80*, 1431–1441.
[23] P. C. Hariharan, J. A. Pople, *Theoret. Chim. Acta* **1973**, *28*, 213–222.
[24] W. J. Hehre, R. Ditchfield, J. A. Pople, *J. Chem. Phys.* **1972**, *56*, 2257–2261.
[25] S. Grimme, S. Ehrlich, L. Goerigk, *Journal of Computational Chemistry* **2011**, *32*, 1456–1465.
[26] Gaussian 16, Revision B.01, M. J. Frisch, G. W. Trucks, H. B. Schlegel, G. E. Scuseria, M. A. Robb, J. R. Cheeseman, G. Scalmani, V. Barone, G. A. Petersson, H. Nakatsuji, X. Li, M. Caricato, A. V. Marenich, J. Bloino, B. G. Janesko, R. Gomperts, B. Mennucci, H. P. Hratchian, J. V. Ortiz, A. F. Izmaylov, J. L. Sonnenberg, D. Williams-Young, F. Ding, F. Lipparini, F. Egidi, J. Goings, B. Peng, A. Petrone, T. Henderson, D. Ranasinghe, V. G. Zakrzewski, J. Gao, N. Rega, G. Zheng, W. Liang, M. Hada, M. Ehara, K. Toyota, R. Fukuda, J. Hasegawa, M. Ishida, T. Nakajima, Y. Honda, O. Kitao, H. Nakai, T. Vreven, K. Throssell, J. A. Montgomery, Jr., J. E. Peralta, F. Ogliaro, M. J. Bearpark, J. J. Heyd, E. N. Brothers, K. N. Kudin, V. N. Staroverov, T. A. Keith, R. Kobayashi, J. Normand, K. Raghavachari, A. P. Rendell, J. C. Burant, S. S. Iyengar, J. Tomasi, M. Cossi, J. M. Millam, M. Klene, C. Adamo, R. Cammi, J. W. Ochterski, R. L. Martin, K. Morokuma, O. Farkas, J. B. Foresman, and D. J. Fox, Gaussian, Inc., Wallingford CT, 2016.

Author Contributions

R. A. K. Shivaraam performed and designed all the experiments, analyzed the data, prepared all the figures and wrote the manuscript; T. Rajeshkumar and L. Maron performed the computational studies, analyzed them and wrote the computational section; R. Scopelliti measured and analyzed the XRD data; I. Zivçovic measured the magnetic data; M. Mazzanti conceived and supervised the project, analyzed the data, wrote the manuscript.
